# Structure of Protein Interaction Networks and Their Implications on Drug Design

**DOI:** 10.1371/journal.pcbi.1000550

**Published:** 2009-10-30

**Authors:** Takeshi Hase, Hiroshi Tanaka, Yasuhiro Suzuki, So Nakagawa, Hiroaki Kitano

**Affiliations:** 1 Department of Bioinformatics, Medical Research Institute, Tokyo Medical and Dental University, Bunkyo-ku, Tokyo, Japan; 2 Department of Bioinformatics, Graduate School of Biomedical Science, Tokyo Medical and Dental University, Bunkyo-ku, Tokyo, Japan; 3 Department of Complex Systems Science, Graduate School of Information Science, Nagoya University, Nagoya, Aichi, Japan; 4 Center for Information Biology and DNA Databank of Japan, National Institute of Genetics, Mishima, Shizuoka, Japan; 5 Sony Computer Science Laboratories, Shinagawa, Tokyo, Japan; 6 The Systems Biology Institute, Minato, Tokyo, Japan; 7 Okinawa Institute of Science and Technology, Kunigami, Okinawa, Japan; University of Chicago, United States of America

## Abstract

Protein-protein interaction networks (PINs) are rich sources of information that enable the network properties of biological systems to be understood. A study of the topological and statistical properties of budding yeast and human PINs revealed that they are scale-rich and configured as highly optimized tolerance (HOT) networks that are similar to the router-level topology of the Internet. This is different from claims that such networks are scale-free and configured through simple preferential-attachment processes. Further analysis revealed that there are extensive interconnections among middle-degree nodes that form the backbone of the networks. Degree distributions of essential genes, synthetic lethal genes, synthetic sick genes, and human drug-target genes indicate that there are advantageous drug targets among nodes with middle- to low-degree nodes. Such network properties provide the rationale for combinatorial drugs that target less prominent nodes to increase synergetic efficacy and create fewer side effects.

## Author Summary

Genome-wide data on interactions between proteins are now available, and networks of protein interactions are the keys to understanding diseases and finding accurate drug targets. This study revealed that the architectural properties of the backbones of protein interaction networks (PINs) were similar to those of the Internet router-level topology by using statistical analyses of genome-wide budding yeast and human PINs. This type of network is known as a highly optimized tolerance (HOT) network that is robust against failures in its components and that ensures high levels of communication. Moreover, we also found that a large number of the most successful drug-target proteins are on the backbone of the human PIN. We made a list of proteins on the backbone of the human PIN, which may help drug companies to search more efficiently for new drug targets.

## Introduction

There is a growing awareness that networks of protein interactions and gene regulations are the keys to understanding diseases and finding accurate drug targets [1]. With the increasing availability of genome-wide data including those on protein interactions and gene expressions, numbers of studies have been done on the structure and statistics of protein interactions and how diseased genes and drug targets are distributed over the network [2],[3]. Understanding the topological and statistical properties of interaction networks and their relationships with lethal genes as well as currently identified drug targets should provide us with insights into robust and fragile properties of networks and possible drug targets for the future. We studied budding-yeast and human protein-protein interaction networks (PINs) to identify the architectural properties of network structures.

PINs have often been argued to be “scale-free” [4],[5], which mostly means they have power-law frequency-degree distributions. However, this definition diverges from the original meaning of being scale-free in terms of the self-similarity of geometric properties of subject systems and there have been reports that claim such distributions are “more normal than normal”; thus, they are not considered to be particularly exotic by themselves [6]. In addition, there are different network topologies with different robustness and performance properties that maintain power-law distributions [7]. Therefore, it is very important to identify the architectural features of the network bearing the specific utilization of analysis results in mind. Our goal in this study was to identify the network topology of PINs and their relationship with lethal genes and possible drug targets so that the statistical likelihood of novel drug targets could be inferred.

A particularly interesting issue in the field of systems engineering, physics, and systems biology is the trade-off between the properties of robustness, fragility, and efficiency. Highly optimized tolerance (HOT) theory is a conceptual framework that can be used to explain this issue. Although a system conforming to HOT theory is optimized for specific perturbations and has highly efficient properties, such a system is extremely fragile against unexpected perturbations [8],[9]. Doyle et al. [8] demonstrated that the Abline Internet2 router-level topology network conformed to HOT theory. Nodes in the Abline network with extremely high-degree nodes connect to a large number of low-degree nodes, while links between these high-degree nodes are suppressed and thus they do not form a core backbone for the whole network. A network having similar structures to the Abline network is defined as a HOTnet [8]. It would be very interesting to clarify whether PINs are HOTnets or not.

The two questions addressed in this paper are: (1) what is the global architecture of PINs? Do they follow the possible architectural features of scale-free networks created by preferential attachments or conform to HOT theory, and (2) are there specific statistical features for proteins that are likely to be drug targets? To answer these questions, budding yeast and human PINs were used to analyze their structural properties using a series of analysis methods.

## Results

Scale-free Network vs. Highly Optimized Tolerance Network: A series of analyses was carried out using budding yeast and human PIN data to identify the topological features of PINs.

In this study, we defined low-degree nodes as nodes with degrees of less than 5 because Han et al. [10] and Partil and Nakamura [11] defined hubs as nodes with degrees of more than 6. We then developed a method called moving stratification by degrees (MSD) to extract sub-networks consisting of hubs with specific degree distributions where indices such as average cluster coefficients would be computed (see Materials and Methods for details). The analyses revealed that the average cluster coefficient was very high for sub-networks consisting of hubs with degrees from 6 to 38, while it was very low for hubs with degrees of more than 39 in the yeast PIN (see Figure S1 and Table S1). Notably, for hubs with degrees of less than 38, the difference in cluster coefficients was generally significant between the yeast PIN and random network, while there were no significant differences in cluster coefficients for hubs with degrees of more than 39 (see Figure S1). Therefore, we defined middle-degree nodes as those with degrees from 6 to 38 and those with degrees of more than 39 as high. In the same manner, we defined middle- (from 6 to 30) and high-degree (more than 31) nodes in the human PIN (see Figure S2 and Table S2). Note that, when we used more stringent thresholds for middle- (from 10 to 50) and high-degree (more than 51) nodes, the results did not change essentially, i.e., the average cluster coefficient for middle-degree nodes was much higher than that for high-degree nodes (see Tables S3 and S4).

**Figure 1:**
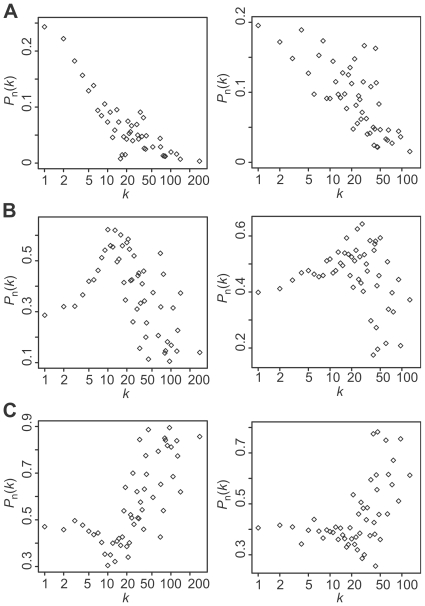
Degree dependent connectivity chart. *P*_n_(*k*) gives the probability that a link of a *k*-degree node is a link to a node in each sub-network of the yeast (left) and human (right) PINs. The value of *P*_n_(*k*) is calculated for a sub-network consisting of high-degree nodes, that consisting of middle-degree nodes, and that consisting of low-degree nodes. (A) Distribution of *P*_n_(*k*) for the high-degree sub-network. (B) Distribution of *P*_n_(*k*) for the middle-degree sub-network. (C) Distribution of *P*_n_(*k*) for the low-degree sub-network.

**Figure 2:**
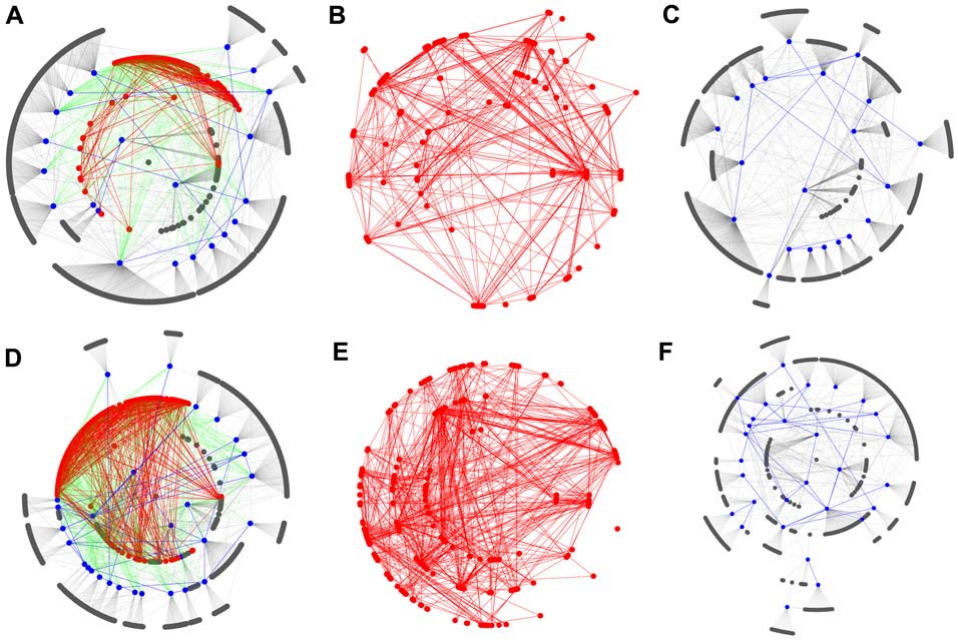
Cloud topology in yeast and human PINs. Grey, red, and blue nodes correspond to low-, middle-, and high-degree nodes. Grey, red, green, and blue links correspond to links between low- and high-degree nodes, those between middle-degree nodes, those between middle- and high-degree nodes, and those between high-degree nodes. For clarity, low- and middle-degree nodes that have no links to high-degree nodes have been omitted. (A) Altocumulus and stratus structures in the yeast PIN. (B) Stratus structure in the yeast PIN. (C) Altocumulus structure in the yeast PIN. (D) Altocumulus and stratus structure in the human PIN. (E) Stratus structure in the human PIN. (F) Altocumulus structure in the human PIN.

The analyses revealed three findings: (1) the network structure for middle-degree nodes (from 6 to 38 for yeast and from 6 to 30 for human PINs), and high-degree nodes (more than 39 for yeast and more than 31 for human PINs) has different structures, (2) middle-degree nodes are tightly connected and form a structure often called a “stratus”, and (3) high-degree nodes do not connect, but connect with low-degree nodes, and form an “altocumulus” structure (Figures 1 and 2). Notably, we used more stringent thresholds for middle- (degrees from 10 to 50) and high-degree nodes (degrees more than 51), and found that changing the thresholds did not essentially affect the results (see Figure S3 and S4). These results suggests that PINs have an architecture where highly interconnected middle-degree nodes form a core backbone for the whole network and large numbers of low-degree nodes connect to high-degree nodes (see Figure 2). This architecture is a type of network that is suggested as a HOTnet, i.e., a network with HOT properties, also seen in the Internet router-level topology [8]. To further confirm this observation, we calculated a graph-theoretic quantity, *s*(*g*), that defines the likelihood high-degree nodes will be connected to one another (see Materials and Methods for details). *S*(*g*), a value normalized against *s*_max_, indicates that networks with tightly interconnected high-degree nodes tend to be closer to 1.0, whereas networks with only sparsely interconnected high-degree nodes tend to be closer to 0.0 (see Materials and Methods for details). Doyle et al. reported randomly generated preferential-attachment-type scale-free networks had relatively high values such as 0.61, whereas a HOTnet exemplified by a network abstracted from an actual Abilene Internet2 router topology network had a value as low as 0.34 [8]. We found that the value of *S*(*g*) for the yeast PIN was 0.25 and that of the human PIN was 0.38. Thus, we could conclude that PINs are HOTnets.

PINs are networks with a modular structure [12]–[14]. Here, modularity is defined as characteristics where there are fewer links between nodes with similar degrees. This only means there are limited links between high-degree nodes (hubs), whereas there are links between hubs and low-degree nodes. This is a feature that was also confirmed in this study (see Figure 2). Modularity in PINs implies that networks have two features [13]: First, functional units may be composed of many low-degree nodes that are directly connected to a hub node. Second, confusion between modules is avoided by avoiding direct connection between hubs. While there are arguments against this claim that hubs are tightly connected because they need to influence one another to achieve an integrated function for the whole system [15], analysis results indicate that such integration is most likely to take place via middle-degree nodes instead of high-degree nodes (see Figure 2).

**Figure 3:**
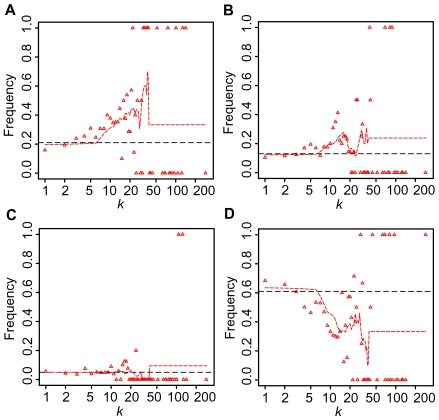
Degree distribution of essential proteins, synthetic lethal proteins, synthetic sick proteins, and proteins that do not belong to any of these (normal proteins). (A) Fraction of essential proteins to all proteins with degree *k* (red triangles). (B) Fraction of synthetic lethal proteins to all proteins with degree *k* (red triangles). (C) Fraction of synthetic sick proteins to all proteins with degree *k* (red triangles). (D) Fraction of normal proteins to all proteins with degree *k* (red triangles). Dashed lines in black give the probability that a randomly selected protein is essential, synthetic lethal, synthetic sick, or normal. Dashed lines in red represent fraction of essential, synthetic lethal, synthetic sick, or normal proteins to all proteins with degree from *k*−5 to *k*+5, when *k*≤38. When *k*>38, dashed lines in red represent fraction of essential, synthetic lethal, synthetic sick, or normal proteins to all proteins with degrees more than 38.

The distribution of essential genes, synthetic genes, and other genes are shown in Figure 3. It is interesting to note that both essential genes and synthetic lethal genes have similar distributions. The average degree of essential proteins is 4.95 and that of synthetic lethal proteins is 4.40. However, the Wilcoxon rank sum test demonstrated that there is no statistical significance between them (*P* = 0.334). In either case, essential and synthetic lethal proteins are concentrated on middle-degree nodes and high-degree nodes. However, the average degree among synthetic sick genes is 4.07 and this is significantly lower than that among synthetic lethal genes (*P* = 0.0015). This means genes that have less severe impact are distributed toward regions with a lower-degree distribution.

**Figure 4:**
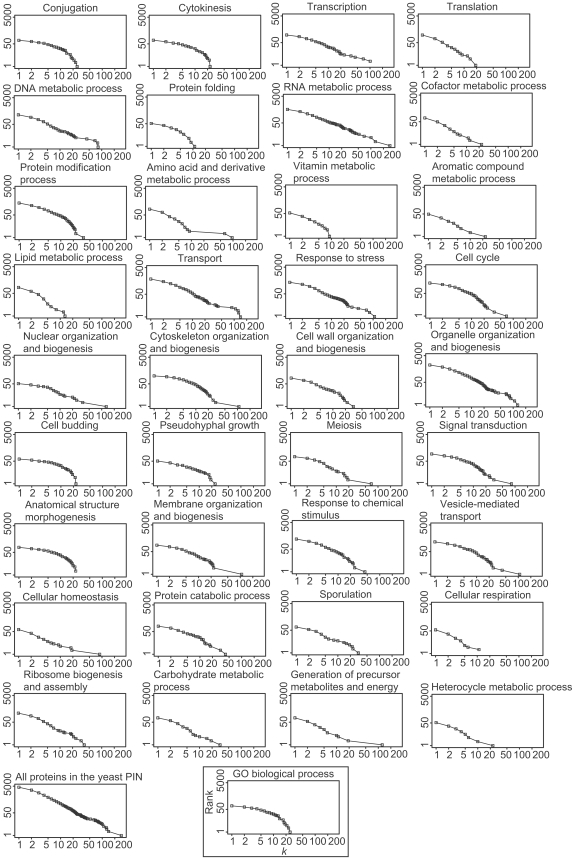
Scale-richness in yeast PIN. Each diagram shows cumulative degree distributions of proteins in each functional group. The name above each diagram denotes the name of the functional category with which the cumulative degree distribution was examined.

Scale-richness: The power law distribution often characterized for scale-free networks only means that local frequency-degree distributions are independent of location along the degree axis, rather than self-similarity of network structures. However, Tanaka demonstrated that bacterial metabolic networks are scale rich in the sense there are different categories of metabolites and enzymes depending on the degree of nodes [16]. A group of nodes with high degree tends to be composed of currency molecules such as ATP and a group of nodes with low degree mostly consists of enzymes involved in specific cellular functions. In this study, we investigated if the frequency-degree distribution of proteins for each functional category exhibited the scale-rich characteristics reported by Tanaka. Figures 4 and S5 correspond to frequency-degree plots for proteins in different functional categories in the yeast PIN and the human PIN. The functional categories were assigned based on the GO slim ontology. As shown in the figures, the degree distribution patterns differ among functional categories. Moreover, proteins with different GO slim annotations have different average degrees (See Tables S5 and S6). Note that many functional categories have significantly higher (or lower) average degrees than the whole PINs (See Tables S5 and S6). These results suggest that the yeast and human PINs are scale-rich.

Drug Targets: Drug-target molecules are distributed over low- to middle-level degree nodes with higher probability on middle-degree nodes. Consistent with reports already published, the average degree among drug-target nodes (4.74) is higher than the average degree among all nodes (4.06).

**Figure 5:**
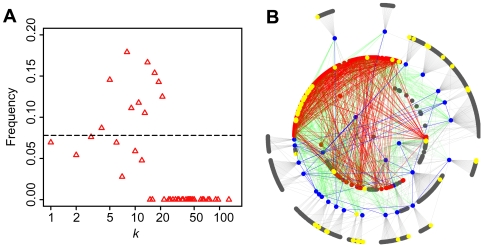
Distribution of drug targets. (A) Degree distribution. Red triangles represent fraction of drug-target proteins to all proteins with degree *k*. The dashed line in black gives the probability that a randomly selected protein is a drug target. (B) Distribution on network topology. Drugs targets (yellow circles) are mapped on human PIN network topology shown in Figure 2D.

**Figure 6:**
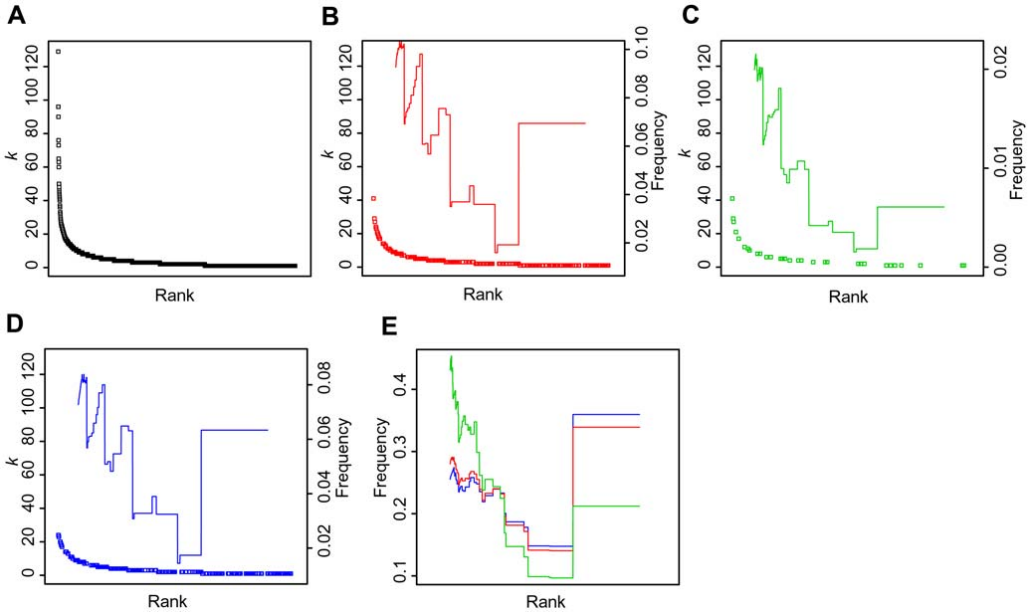
The long tails in degree distribution of drug targets, targets for cancer, and those for non-cancerous diseases. Proteins were ranked in decreasing order of their degree *k*. (A) Rank of a protein with degree *k*. (B) Rank of a drug target with degree *k*. (C) Rank of a target for cancer diseases with degree *k*. (D) Rank of a target for non-cancerous diseases with degree *k*. Red, green, and blue lines represent fraction of drug targets, targets for cancer diseases, and those for non-cancerous diseases to all proteins with rank from rank−0.1*N* to rank+0.1*N* (*N* represents number of proteins in the human PIN). (E) Red, green, and blue lines represent fraction of drug targets, targets for cancer disease, and those for non-cancerous disease with rank from rank−0.1*N* to rank+0.1*N* to all drug targets, all targets for cancer diseases, and those for non-cancerous diseases, respectively.

The distribution of known drug targets is shown in Figure 5 and this is predominantly distributed to middle-degree nodes and mostly on backbone of the network. There are almost no drug targets for high-degree nodes. The distribution of drug targets for cancer and non-cancerous diseases are in sharp contrast. While the average degree of target nodes for cancer drugs was 7.82, the targets for non-cancerous diseases scored only 4.24 (*P* = 0.01). Moreover, we found that the proportion of drug targets among low-degree proteins were similar to random expectation. Figure 6 shows distribution of drug targets marked on degree-rank plot. The drug target molecule that has highest degree is Src with 41 which is the target for drugs such as Dasatinib. Target molecules for anti-cancer drugs are shifted toward high degree nodes compare against average and non-anti-cancer drugs.

## Discussion

A series of analyses revealed that both the budding yeast and human PINs are scale-rich and have HOT networks. There are extensive interconnections among middle-degree nodes that form the backbone of the network (see Figure 2). Most drug-target genes concentrate on middle-degree nodes and parts of low-degree nodes, but not on high-degree nodes. Interestingly, Feldman et al. (2008) [17] reported that genes harboring inherited disease mutations also concentrated on middle-degree nodes. Because of the potential lethality observed in budding yeast (Figure 3A) and reported high lethality in mouse knockout [2], high-degree nodes are unlikely to be preferred drug targets or genes with disease mutations. Since oncogenes tend to be high-degree nodes, they are less likely to be drug targets, or one has to accept major potential side effects. The fact that the degree distribution of cancer-drug targets is higher than that of non-cancer-drug targets is consistent with the report by Yao and Rzhetsky [18]. Since high-degree nodes are predominantly connected with low-degree nodes (Figures 1, 2, S3, and S4), the elimination of high-degree nodes is likely to affect large numbers of low-degree nodes. This may result in unacceptable side effects since a group of genes that bear certain functions may be made collectively dysfunctional. Detailed case studies are warranted to test and verify this possible interpretation. However, the average degree distribution of synthetic sick genes (4.07) is less than that of essential genes (4.95) and synthetic lethal genes (4.40). This implies that a drug design strategy to generate synergetic effects by targeting less important targets can be a reasonable option because each compound in such drugs can select targets that have less impact on the overall system alone.

We found that middle-level degree nodes are the optimal targets for therapeutic drugs. A similar observation was reported by Yao and Rzhetsky [18], although they measured the mean degree among drug targets. In this study, we investigated the degree distribution of drug targets in greater detail, because we measured a fraction of drug targets to all nodes with degree *k* as well as mapping drug targets on the network structure. It was clearly identified most of drug targets for drugs that are currently on the market are concentrated on middle degree nodes that are back bone of the network and low-degree nodes that tends to have specific function specific effects. One of novel findings here is that the distribution of drug targets for low-degree nodes is similar to random expectation, indicating that there are a certain number of low-degree drug targets. From these results, we can expect that the most advantageous targets for combinatorial drugs could be among low-degree nodes because these could have less severe impact on the overall system of the human body. This is consistent with the idea of “long-tail drugs”[19].

Are there any relationships between structures in molecular networks (i.e., scale-richness in PINs) and the properties of their underlying genome? Rzhetsky and Gomez [20] proposed a stochastic model describing the evolutionary growth of molecular networks. Their model predicts that, in a molecular network, the shape of the degree distribution will be similar to the shape of the distribution of domains in the genome. Actually, they showed that, in the case of the entire yeast PIN, both the degree distribution and the distribution of the domain followed a power law. Therefore, it might be interesting to see whether, for each functional category, the shape of the degree distribution was similar to that of the domain distribution, when the entire architecture of domains in genomes becomes available.

In this study, we assumed that the PINs represented all functions of genes. However, the PINs are just composed of binary protein-protein binding and proteins have other types of functions, such as catalyzing reactions with non-protein substrates. Therefore, PINs reflect a subset of the entire cellular function. This indicates that, if the complete picture for cellular protein functions could be considered, our conclusions from the PINs may diverge from what we presented here. Moreover, at present, the yeast and human PINs represent incomplete pictures of the actual entire PINs of these organisms. When data on all the actual entire PINs become available, we intend to examine all the actual entire PINs to see whether similar observations to those in this study can be made or not.

It is interesting to note that both PINs and the Internet topology are HOTnets. Many of the observed properties in Internet router topology may be applied to PINs as well. Such properties include robustness against node failure and optimized performance [21]. It has been reported that analysis using several possible router topologies found that a HOTnet configuration was most efficient, providing more maximum overall bandwidth to users than that with other network-configuration approaches such as random and preferential attachment [21]. The implication is that biological PINs have evolved to become efficient and error tolerant. The series of analyses presented in this report indicate that there are changes whereby we can rationally design drugs by taking into account network properties, and additional insights from engineering and physics may further extend our opportunities for exploring network-based biology.

## Materials and Methods

### 

#### PINs, GO data, and essential genes

Yeast PIN data were obtained from the Munich Information Center for Protein Sequences (MIPS) database (http://mips.gsf.de) [22] and human PIN data were obtained from Rual et al. [23]. The yeast (or human) PIN contained 4,153 (or 3,023) proteins and 7,417 (or 6,149) non-redundant interactions. The GO slim dataset for the yeast PIN was from the ftp site of the Saccharomyces Genome Database (SGD) (ftp://genome-ftp.stanford.edu/pub/yeast/literature_curation/) [22] and that for the human PIN was from the European Bioinformatics Institute (EBI) (ftp://ftp.ebi.ac.uk/pub/databases/GO/goa/HUMAN/). The list of essential genes from SGD [22] contained 889 essential genes that were mapped to the yeast PIN.

#### Synthetic lethal and synthetic sick proteins

We obtained a list of synthetic lethal and sick interactions from Tong et al. [24]. There were 735 proteins having at least one synthetic lethal interaction and we defined these proteins as synthetic lethal proteins. However, there were 816 proteins having at least one synthetic sick interaction, of which 310 proteins had no synthetic lethal interactions. We defined these 310 proteins as synthetic sick proteins. 538 synthetic lethal proteins and 209 synthetic sick proteins were mapped to the yeast PIN.

#### Drug-target proteins

To analyze the statistical features of drug-target genes, we obtained a list of proteins that were targets of FDA-approved and experimental drugs from Yildirim et al. [3]. This list contained 1,013 drug-target proteins, of which 236 were mapped to the human PIN. To generate a list of drug-disease associations, we mapped drugs to diseases by investigating information on drugs obtained from the DrugBank database [25] (information on drugs is contained in the “indications” field in the DrugBank database). Then, by using the list of drug-disease associations, we divided drug-target proteins into two groups: target proteins for cancer drugs and those for non-cancerous diseases. The human PIN contained 33 target proteins for cancer and 203 for non-cancerous diseases.

#### Random network

We generated a random network by using the method proposed by Maslov and Sneppen [13], where the following procedures were performed. First, two links in a network were chosen randomly. Assume that one link connects nodes A and B, and the other connects nodes C and D. Second, these links were rewired by exchanging their connecting partners. That is, nodes A and D were connected, and nodes B and C were connected. We repeated these two procedures 1,000*E* times (*E* is the number of interactions in the original network) to generate a random network. Note that the method did not alter the degree distribution.

#### Cluster coefficient

The cluster coefficient of node i is defined as *C*_i_ = 2*e*_i_/*k*_i_(*k*_i_−1), where *k*_i_ is the degree of node i and *e*_i_ is the number of links connecting *k*_i_ neighbors of node i to one another [26]. When *k*_i_ is zero or one, *C*_i_ is defined as zero. *C*_i_ is equal to one when all neighbors of node i are fully connected to one another, while *C*_i_ is zero when none of the neighbors are connected to one another.

#### Moving stratification by degree

A method of analysis termed moving stratification by degree (MSD) was developed and used to compare three networks, the budding yeast PIN, the human PIN, and a randomly generated network with exactly the same degree distribution as the PINs. Hubs were defined as nodes with degrees of more than six [10],[11]. MSD was used to extract sub-networks consisting of hubs with degrees from *k*_c_−*μ* to *k*_c_+*μ*. In this study, we used *μ* = 1, 3, 5, and 7. Since a hub is defined as a node with degrees of more than 6, we used initial values of *k*_c_ = 7, 9, 11, and 13. Then, *k*_c_ was scanned up to 300 with step size 1. For each initial value (*k*_c_ = 7, 9, 11, and 13), MSD extracted 293, 291, 289, and 287 sub-networks, respectively. For these sub-networks, only hub nodes were included. In the following analysis, data from *μ* = 5 were used because changing *μ* did not significantly alter the results.

The average cluster coefficient <*C*(*k*_c_)>, average shortest path length <*L*(*k*_c_)>, betweeness centrality *B*_t_(*k*_c_), and node ratio included in largest components *G*_c_(*k*_c_) in each sub-network from the PINs were compared with each value from random networks. The sub-networks were tightly connected when the average cluster coefficient was high. While there were no significant differences in the average cluster coefficient between the PINs and random networks for high *k*_c_ (*k*_c_>38 for the yeast PIN and *k*_c_>30 for the human PIN) (Figures S1A and S2A), the average cluster coefficient for PINs was significantly higher than that for the random networks. There were no significant differences in <*L*(*k*_c_)> and *G*_c_(*k*_c_) between the PINs and random networks (Figures S1B, S1C, S2B, and S2C). It is interesting to note that there were no significant differences in global properties (i.e., betweeness centrality *B*_t_(*k*_c_)) between PINs and random networks (see Figures S1D and S2D), although difference in local properties (i.e., average cluster coefficient <*C*(*k*_c_)>) were significant between PINs and random networks (see Figures S1A and S2A).

The fraction of essential proteins to all proteins in each sub-network (*F*_LC_(*k*_c_)) was investigated for the budding yeast PIN (Figure S1E). The fraction of drug targets to all proteins in each sub-network (*F*_DT_(*k*_c_)) was investigated for the human PIN (Figure S2E).

There were no known drug-target proteins when *k*_c_ was over 50 (*F*_DT_(*k*_c_) = 0 for *k*_c_>50. See Table S4). This means that high-degree proteins were unlikely to be drug targets. However, *F*_DT_(*k*_c_) is significantly higher than random expectation when *k*_c_ is between 11 and 32. Thus, middle-degree proteins are biologically important and can be drug-target proteins. Table S7 lists middle-degree proteins and their functions categorized by GO annotation. We can expect novel drug targets to be included in the list.

Further analyses were carried out by partitioning a network into three sub-networks, a sub-network consisting of low-degree nodes (degrees from 1 to 5), that consisting of middle-level degree nodes (degrees from 6 to 38 for the yeast PIN and from 6 to 30 for the human PIN), and that consisting of high-degree nodes (degrees more than 39 for the yeast PIN and more than 31 for the human PIN). Middle-level nodes formed a tightly coupled stratus structure whereas high-degree nodes formed a modularized altocumulus structure.

Tables S1 and S2 show that middle-degree nodes formed a high-density tightly coupled structure and a middle-degree sub-network had higher average cluster coefficients than other sub-networks. The average cluster coefficient of PINs without nodes in the middle-degree sub-network was substantially lower than that of the original PIN. In addition, the average shortest distance in the middle-degree sub-network was almost equal to that of the entire PINs. Most nodes in the entire PINs or middle-degree sub-network (over 95% of nodes) were included in the largest component. However, this is not a case for low-degree or high-degree sub-networks. Thus, the characteristics of middle-degree sub-networks strongly influence the statistical characteristics of the whole PIN. The whole network architecture seems to have tightly connected middle-degree nodes that are connected to high-degree nodes, and a large number of low-degree nodes are mostly connected to high-degree nodes (see Figure 2). Moreover, we used more stringent thresholds for middle- and high-degree nodes and found that changing the thresholds did not essentially affect the results (i.e., the average cluster coefficient, average shortest path length, or *G*_C_) (see Tables S3 and S4).

The series of analyses thus far indicates that the functional role for proteins included in low-degree, middle-degree, and high-degree sub-networks are totally different. This means that the yeast and human PINs are not scale-free in terms of the composition of the functional role of proteins. Proteins with each functional group have a characteristic degree distribution. To investigate the degree distribution of proteins in each functional category, we annotated proteins in the yeast and human PINs by using the GO slim biological process ontology. As shown in Figures 4 and S5, there are different degree-distribution patterns for proteins from different functional categories. This suggests that a scale-free distribution emerges from the composition of different functional protein groups each of which has scale-dependent degree distributions. Thus, from the functional distribution, the yeast and human PINs are scale-rich.

#### *S*(*g*) value

Before giving a definition for the *S*(*g*) value, let us first define some notations. Let *n* be the number of nodes in a network and *k*_i_ be the degree of node i. *D* = {*k*_1_,*k*_2_,…,*k*_n_} represents a given degree distribution and *G*(*D*) denotes the set of all connected networks having the same degree distribution, *D*. For a network, *g*, having degree distribution *D*, graph-theoretic quantity *s*(*g*) is defined as *s*(*g*) = Σ_(i,j)∈*E*(*g*)_ *k*_i_ *k*_j_, where *E*(*g*) is the set of links in the network. *s*_max_ is defined as *s*_max_ = max{*s*(*g*): *g*∈*G*(*D*)} and we calculated the value of *s*_max_ by using the algorithm devised by Alderson et al. [7]. *S*(*g*), the value normalized against *s*_max_, is defined as *S*(*g*) = *s*(*g*)/*s*_max_ [8]. In this paper, we calculated the value of *S*(*g*) in the yeast and human PINs.

## Supporting Information

Figure S1 Statistics of sub-networks generated by MSD (yeast PIN). Red triangles and black squares show the values for the yeast PIN and random network, respectively. The results for random network were obtained by taking the average among 100 random networks. (A) Distribution of <C(kC)>. (B) Distribution of <L(kC)>. (C) Distribution of GC(kC). (D) Distribution of PLC(kC). The dashed line represents the probability that a randomly selected protein is a lethal protein.
(1.37 MB TIF)

Figure S2 Statistics of sub-networks generated by MSD (human PIN). Red triangles and black squares show the values for the human PIN and random network, respectively. The results for random network were obtained by taking the average among 100 random networks. (A) Distribution of <C(kC)>. (B) Distribution of <L(kC)>. (C) Distribution of GC(kC). (D) Distribution of PDT(kC). The dashed line in black represents the probability that a randomly selected protein is a drug target.
(1.35 MB TIF)

Figure S3 Degree Dependent Connectivity Chart with stringent thresholds. Pn(k) gives the probability that a link of a k-degree node is a link to a node in each sub-network of the yeast (left) and human (right) PINs. The value of Pn(k) is calculated for a sub-network consisting of high-degree nodes, that consisting of middle-degree nodes, and that consisting of low-degree nodes. (A) Distribution of Pn(k) for the high-degree sub-network. (B) Distribution of Pn(k) for the middle-degree sub-network. (C) Distribution of Pn(k) for the low-degree sub-network.
(0.26 MB TIF)

Figure S4 Cloud topologies in yeast and human PINs with stringent thresholds. Grey, red, and blue nodes correspond to low-, middle-, and high-degree nodes. Grey, red, green, and blue links correspond to links between low- and high-degree nodes, those between middle-degree nodes, those between middle- and high-degree nodes, and those between high-degree nodes. For clarity, low- and middle-degree nodes that have no links to high-degree nodes have been omitted. (A) Altocumulus and stratus structures in the yeast PIN. (B) Stratus structure in the yeast PIN. (C) Altocumulus structure in the yeast PIN. (D) Altocumulus and stratus structure in the human PIN. (E) Stratus structure in the human PIN. (F) Altocumulus structure in the human PIN.
(2.83 MB TIF)

Figure S5 Scale-richness in human PIN. Each diagram shows cumulative degree distributions of proteins in each functional group. The name above each diagram denotes the name of the functional category with which the cumulative degree distribution was examined.
(0.45 MB TIF)

Table S1 Statistics of sub-networks in the yeast PIN. a. number of nodes b. average shortest path legth c. fraction of nodes contained in a largest component to all nodes contained in a sub-network d. average cluster coefficient e. betweeness centrality f. fraction of essential nodes to all nodes contained in a sub-network g. a sub-network consist of low-degree nodes h. a sub-network consist of middle-degree nodes i. a sub-network consist of high-degree nodes j. a sub-network consist of low- and middle-degree nodes k. a sub-network consist of low- and high-degree nodes.
(0.04 MB DOC)

Table S2 Statistics of sub-networks in the human PIN. a. See Table S1. b. fraction of drug-target nodes contained in a sub-network to all nodes contained in the sub-network.
(0.04 MB DOC)

Table S3 Statistics of sub-networks in yeast PIN with stringent thresholds for middle- and high-degree nodes. a. See Table S1.
(0.04 MB DOC)

Table S4 Statistics of sub-networks in human PIN with stringent thresholds for middle- and high-degree nodes. a. See Table S1.
(0.04 MB DOC)

Table S5 Degrees of the genes in yeast PIN belonging to each functional category. a. Mean degree among the proteins contained in each functional category. b. Number of proteins in each functional category. c. ***, **, and * represents that a given value is significantly higher (or lower) than average degree among proteins belonging other functional categories with P<0.001, P<0.01, and P<0.05, respectively, by the Wilcoxon rank-sum two-sample test with the Bonferronni correction.
(0.06 MB DOC)

Table S6 Degrees of the genes in human PIN belonging to each functional category. a. See Table S5.
(0.04 MB DOC)

Table S7 Middle degree proteins in human PIN and their functions.
(2.79 MB DOC)
