# Supplementary figures and images for: Structure of Protein Interaction Networks and Their Implications on Drug Design
Source: PLoS Comput Biol. 2009 Oct 30;5(10):e1000550. doi: 10.1371/journal.pcbi.1000550 (PMC2760708; doi:10.1371/journal.pcbi.1000550)

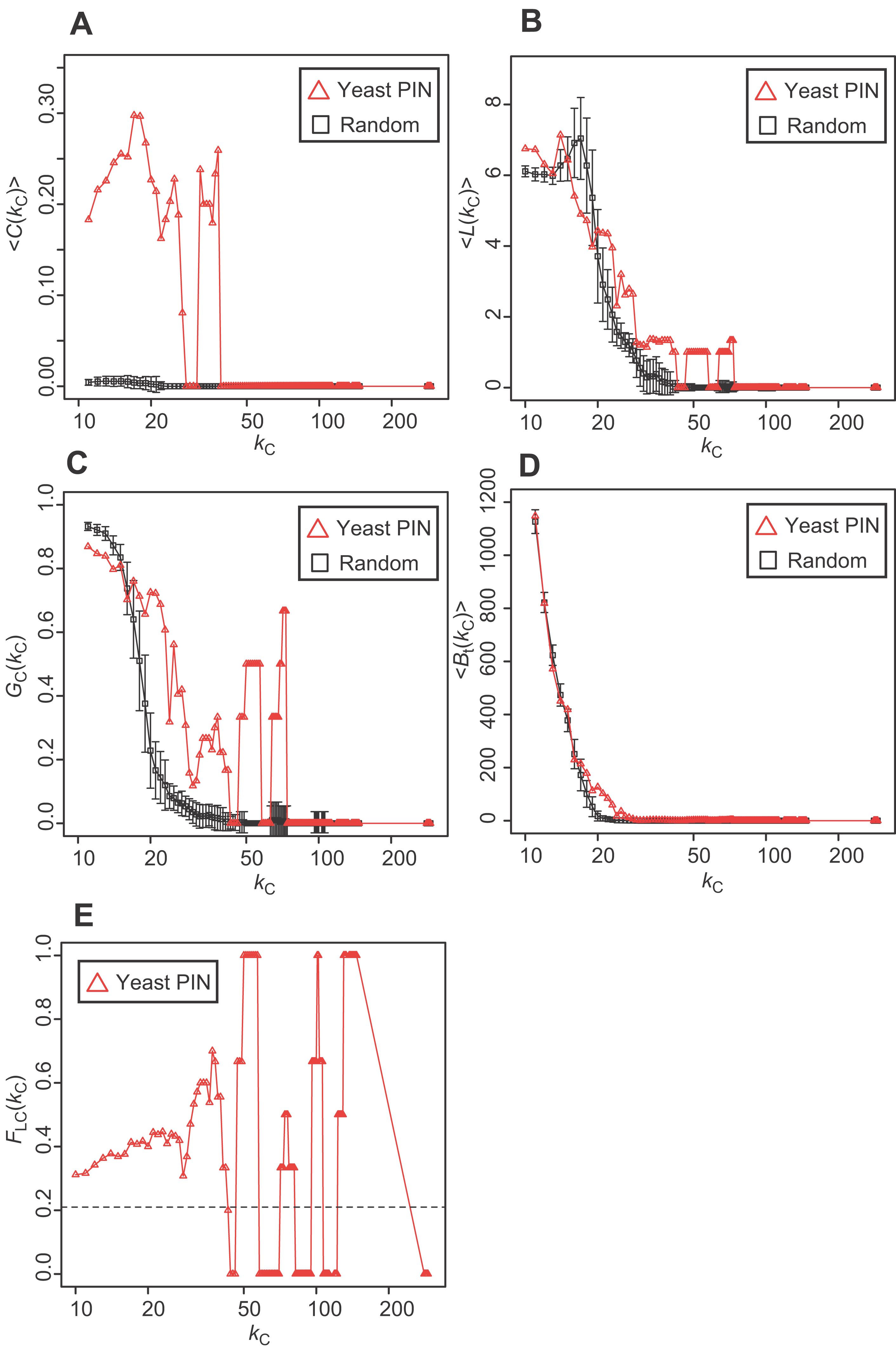

Supplement: Figure S1 — Statistics of sub-networks generated by MSD (yeast PIN). Red triangles and black squares show the values for the yeast PIN and random network, respectively. The results for random network were obtained by taking the average among 100 random networks. (A) Distribution of <C(kC)>. (B) Distribution of <L(kC)>. (C) Distribution of GC(kC). (D) Distribution of PLC(kC). The dashed line represents the probability that a randomly selected protein is a lethal protein. (1.37 MB TIF) [file pcbi.1000550.s001.tif]

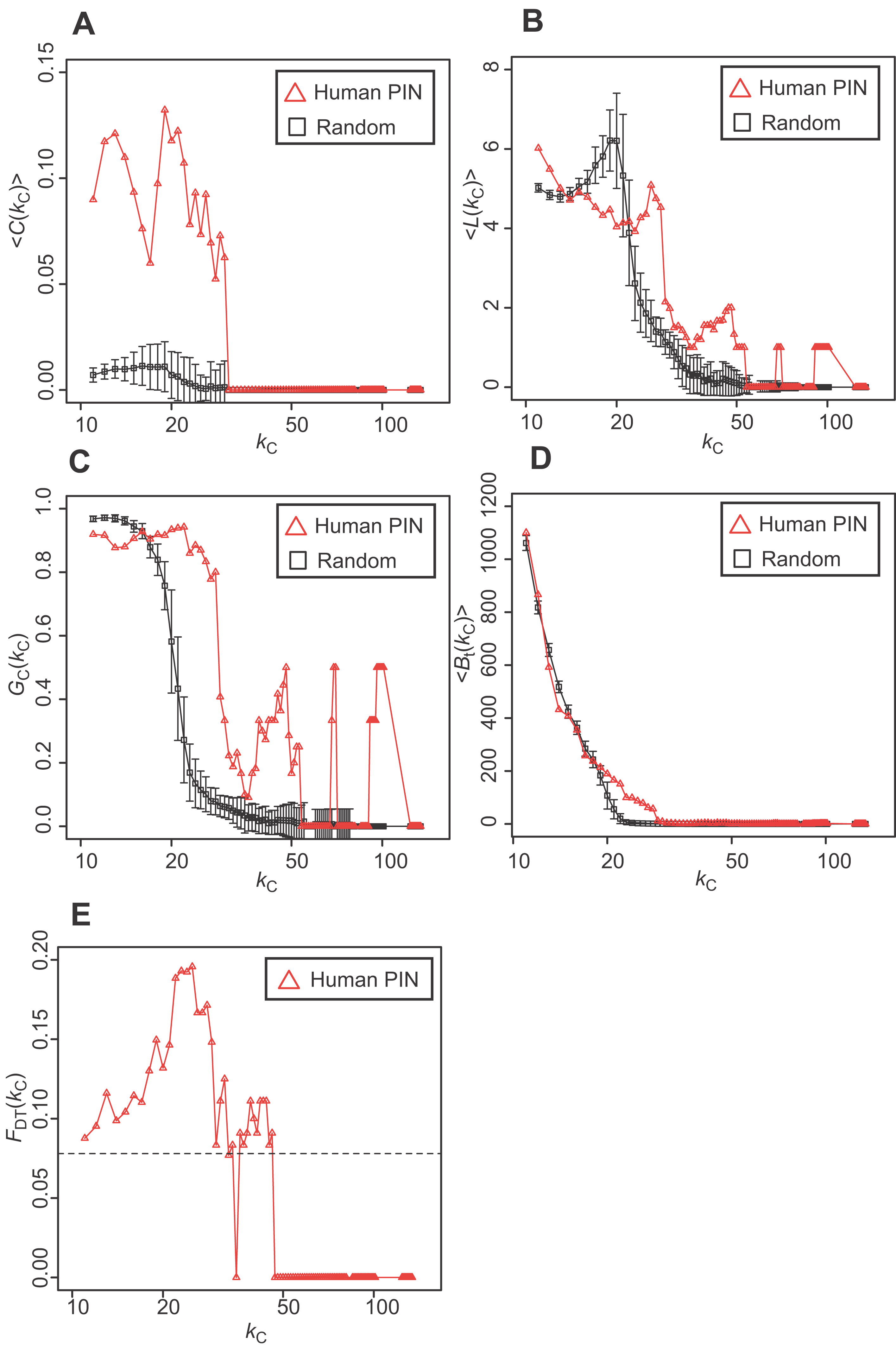

Supplement: Figure S2 — Statistics of sub-networks generated by MSD (human PIN). Red triangles and black squares show the values for the human PIN and random network, respectively. The results for random network were obtained by taking the average among 100 random networks. (A) Distribution of <C(kC)>. (B) Distribution of <L(kC)>. (C) Distribution of GC(kC). (D) Distribution of PDT(kC). The dashed line in black represents the probability that a randomly selected protein is a drug target. (1.35 MB TIF) [file pcbi.1000550.s002.tif]

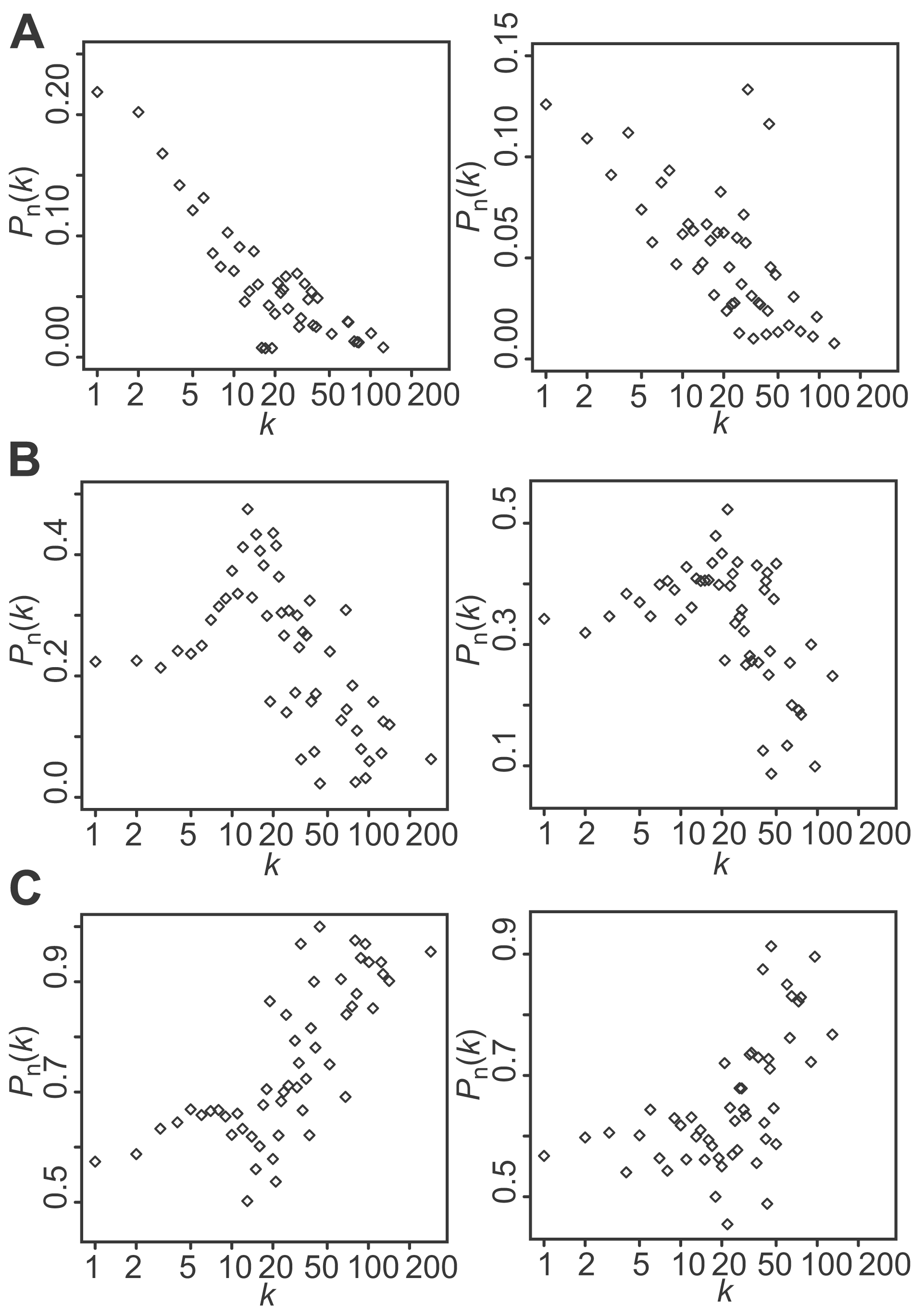

Supplement: Figure S3 — Degree Dependent Connectivity Chart with stringent thresholds. Pn(k) gives the probability that a link of a k-degree node is a link to a node in each sub-network of the yeast (left) and human (right) PINs. The value of Pn(k) is calculated for a sub-network consisting of high-degree nodes, that consisting of middle-degree nodes, and that consisting of low-degree nodes. (A) Distribution of Pn(k) for the high-degree sub-network. (B) Distribution of Pn(k) for the middle-degree sub-network. (C) Distribution of Pn(k) for the low-degree sub-network. (0.26 MB TIF) [file pcbi.1000550.s003.tif]

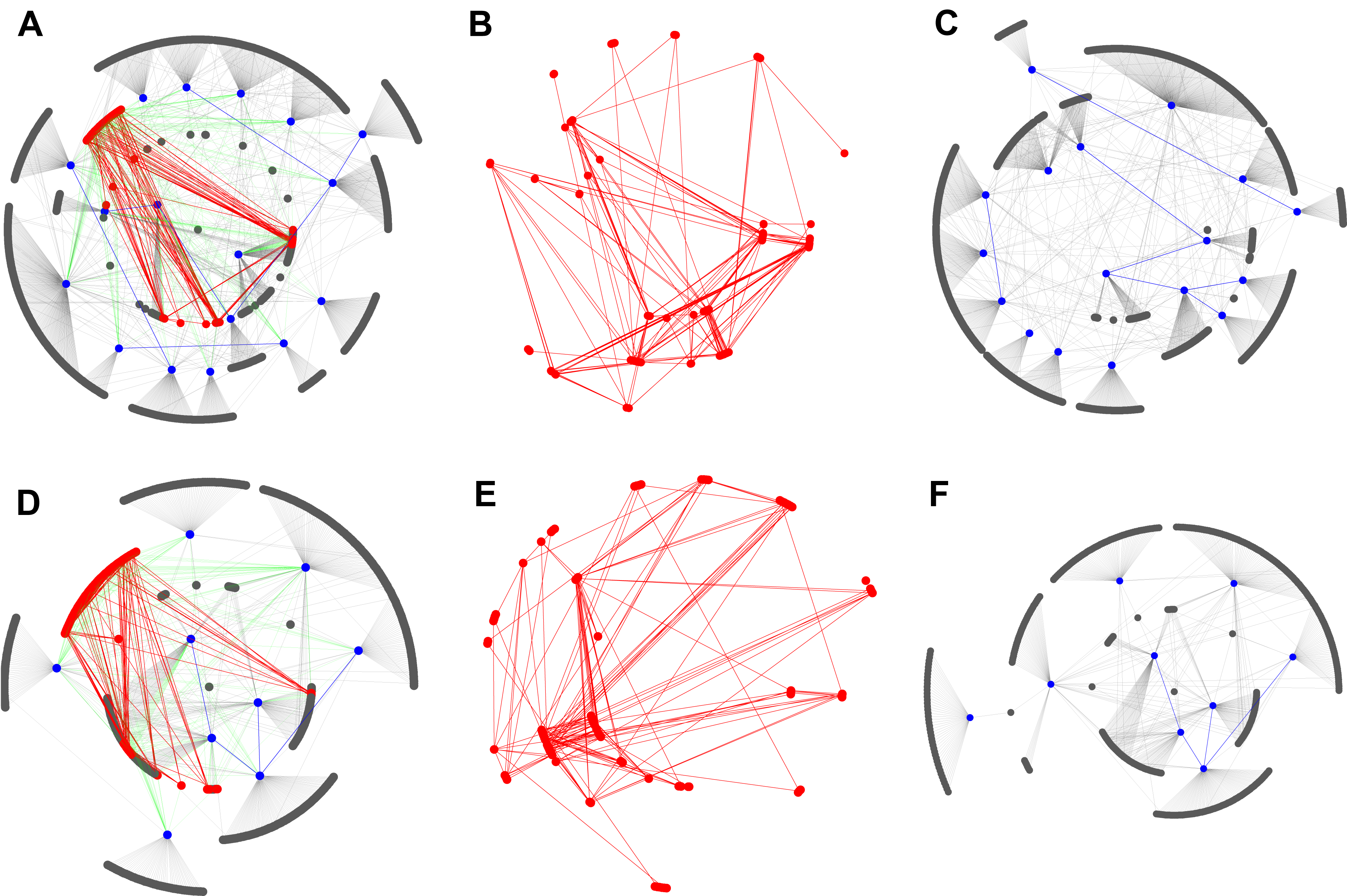

Supplement: Figure S4 — Cloud topologies in yeast and human PINs with stringent thresholds. Grey, red, and blue nodes correspond to low-, middle-, and high-degree nodes. Grey, red, green, and blue links correspond to links between low- and high-degree nodes, those between middle-degree nodes, those between middle- and high-degree nodes, and those between high-degree nodes. For clarity, low- and middle-degree nodes that have no links to high-degree nodes have been omitted. (A) Altocumulus and stratus structures in the yeast PIN. (B) Stratus structure in the yeast PIN. (C) Altocumulus structure in the yeast PIN. (D) Altocumulus and stratus structure in the human PIN. (E) Stratus structure in the human PIN. (F) Altocumulus structure in the human PIN. (2.83 MB TIF) [file pcbi.1000550.s004.tif]

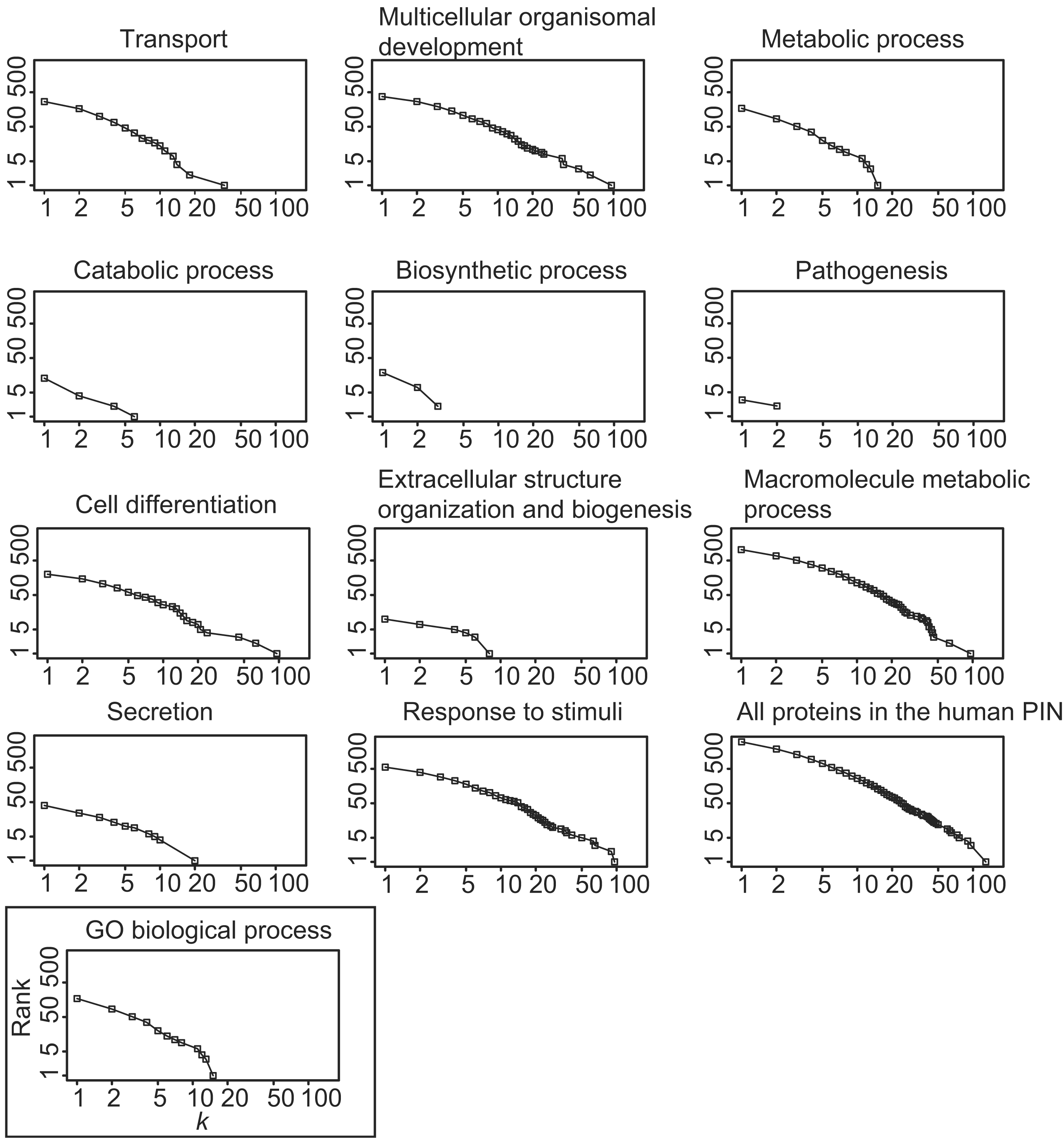

Supplement: Figure S5 — Scale-richness in human PIN. Each diagram shows cumulative degree distributions of proteins in each functional group. The name above each diagram denotes the name of the functional category with which the cumulative degree distribution was examined. (0.45 MB TIF) [file pcbi.1000550.s005.tif]
