# Supplementary material for: Structure of Protein Interaction Networks and Their Implications on Drug Design
Source: PLoS Comput Biol. 2009 Oct 30;5(10):e1000550. doi: 10.1371/journal.pcbi.1000550 (PMC2760708; doi:10.1371/journal.pcbi.1000550)
Supplement: Table S1 — Statistics of sub-networks in the yeast PIN. a. number of nodes b. average shortest path legth c. fraction of nodes contained in a largest component to all nodes contained in a sub-network d. average cluster coefficient e. betweeness centrality f. fraction of essential nodes to all nodes contained in a sub-network g. a sub-network consist of low-degree nodes h. a sub-network consist of middle-degree nodes i. a sub-network consist of high-degree nodes j. a sub-network consist of low- and middle-degree nodes k. a sub-network consist of low- and high-degree nodes. (0.04 MB DOC) [file pcbi.1000550.s006.doc]

**Table S1. Statistics of sub-networks in yeast PIN.**

| Sub-networks | *N*^a^ | <*L*>^b^ | *G*_C_^c^ | <*C*>^d^ | *B*t^e^ | *P*_LC_^f^ |
| --- | --- | --- | --- | --- | --- | --- |
| Low degree nodes^g^ | 3604 | 3.42 | 0.01 | 0.019 | 8.44 | 0.20 |
| Middle degree nodes^h^ | 528 | 4.45 | 0.97 | 0.210 | 1320.47 | 0.34 |
| High degree nodes^i^ | 21 | 2.76 | 0.43 | 0.000 | 10.05 | 0.33 |
| Low+middle^j^ | 4132 | 6.27 | 0.76 | 0.059 | 8865.01 | 0.21 |
| Low+high^k^ | 3625 | 5.37 | 0.61 | 0.022 | 5430.58 | 0.20 |
| yeast PIN | 4153 | 4.85 | 0.94 | 0.062 | 13419.11 | 0.21 |

1. number of nodes
2. average shortest path legth
3. fraction of nodes contained in a largest component to all nodes contained in a sub-network
4. average cluster coefficient
5. betweeness centrality
6. fraction of essential nodes to all nodes contained in a sub-network
7. a sub-network consist of low-degree nodes
8. a sub-network consist of middle-degree nodes
9. a sub-network consist of high-degree nodes
10. a sub-network consist of low- and middle-degree nodes
11. a sub-network consist of low- and high-degree nodes
