# Supplementary material for: Structure of Protein Interaction Networks and Their Implications on Drug Design
Source: PLoS Comput Biol. 2009 Oct 30;5(10):e1000550. doi: 10.1371/journal.pcbi.1000550 (PMC2760708; doi:10.1371/journal.pcbi.1000550)
Supplement: Table S2 — Statistics of sub-networks in the human PIN. a. See Table S1. b. fraction of drug-target nodes contained in a sub-network to all nodes contained in the sub-network. (0.04 MB DOC) [file pcbi.1000550.s007.doc]

**Table S2. Statistics of sub-networks in human PIN.**

| Sub-networks | *N*^a^ | <*L*>^a^ | *G*_C_^a^ | <*C*>^a^ | *B*t^a^ | *P*_DT_^b^ |
| --- | --- | --- | --- | --- | --- | --- |
| Low degree node^a^ | 2475 | 4.12 | 0.02 | 0.015 | 36.67 | 0.074 |
| Middle degree node^a^ | 519 | 4.18 | 0.97 | 0.104 | 1165.64 | 0.102 |
| High degree node^a^ | 29 | 2.75 | 0.72 | 0.030 | 13.90 | 0.034 |
| Low + middle ^a^ | 2994 | 5.67 | 0.81 | 0.054 | 7728.79 | 0.078 |
| Low + high ^a^ | 2504 | 5.73 | 0.47 | 0.028 | 2375.63 | 0.073 |
| human PIN | 3023 | 4.84 | 0.92 | 0.066 | 9475.26 | 0.078 |

1. See Table S1.
2. fraction of drug-target nodes contained in a sub-network to all nodes contained in the sub-network
