# Supplementary material for: Structure of Protein Interaction Networks and Their Implications on Drug Design
Source: PLoS Comput Biol. 2009 Oct 30;5(10):e1000550. doi: 10.1371/journal.pcbi.1000550 (PMC2760708; doi:10.1371/journal.pcbi.1000550)
Supplement: Table S3 — Statistics of sub-networks in yeast PIN with stringent thresholds for middle- and high-degree nodes. a. See Table S1. (0.04 MB DOC) [file pcbi.1000550.s008.doc]

**Table S3. Statistics of sub-networks in yeast PIN with stringent thresholds for middle- and high-degree nodes.**

| Sub-networks | *N*^a^ | <*L*>^a^ | *G*_C_^a^ | <*C*>^a^ | *B*t^a^ | *P*_LC_^a^ |
| --- | --- | --- | --- | --- | --- | --- |
| Low degree nodes^a^ | 3909 | 13.33 | 0.45 | 0.035 | 5466.88 | 0.20 |
| Middle degree nodes^a^ | 227 | 4.10 | 0.95 | 0.260 | 517.21 | 0.39 |
| High degree nodes^a^ | 17 | 2.00 | 0.29 | 0.000 | 3.59 | 0.41 |
| Low+middle^a^ | 4136 | 6.13 | 0.78 | 0.059 | 10410.88 | 0.21 |
| Low+high^a^ | 3926 | 5.52 | 0.76 | 0.037 | 8697.62 | 0.20 |
| yeast PIN | 4153 | 4.85 | 0.94 | 0.062 | 13419.11 | 0.21 |

1. See Table S1.
