# Supplementary material for: Structure of Protein Interaction Networks and Their Implications on Drug Design
Source: PLoS Comput Biol. 2009 Oct 30;5(10):e1000550. doi: 10.1371/journal.pcbi.1000550 (PMC2760708; doi:10.1371/journal.pcbi.1000550)
Supplement: Table S4 — Statistics of sub-networks in human PIN with stringent thresholds for middle- and high-degree nodes. a. See Table S1. (0.04 MB DOC) [file pcbi.1000550.s009.doc]

**Table S4. Statistics of sub-networks in human PIN with stringent thresholds for middle- and high-degree nodes.**

| Sub-networks | *N*^a^ | <*L*>^a^ | *G*_C_^a^ | <*C*>^a^ | *B*t^a^ | *P*_DT_^a^ |
| --- | --- | --- | --- | --- | --- | --- |
| Low degree node^a^ | 2760 | 12.72 | 0.46 | 0.034 | 4008.37 | 0.075 |
| Middle degree node^a^ | 254 | 3.42 | 0.98 | 0.142 | 489.81 | 0.110 |
| High degree node^a^ | 9 | 1.80 | 0.44 | 0.000 | 4.11 | 0.000 |
| Low + middle ^a^ | 3014 | 5.22 | 0.86 | 0.060 | 8481.49 | 0.078 |
| Low + high ^a^ | 2769 | 7.36 | 0.65 | 0.041 | 5449.79 | 0.075 |
| human PIN | 3023 | 4.84 | 0.92 | 0.066 | 9475.26 | 0.078 |

1. See Table S2.
