# Supplementary material for: Structure of Protein Interaction Networks and Their Implications on Drug Design
Source: PLoS Comput Biol. 2009 Oct 30;5(10):e1000550. doi: 10.1371/journal.pcbi.1000550 (PMC2760708; doi:10.1371/journal.pcbi.1000550)
Supplement: Table S5 — Degrees of the genes in yeast PIN belonging to each functional category. a. Mean degree among the proteins contained in each functional category. b. Number of proteins in each functional category. c. ***, **, and * represents that a given value is significantly higher (or lower) than average degree among proteins belonging other functional categories with P<0.001, P<0.01, and P<0.05, respectively, by the Wilcoxon rank-sum two-sample test with the Bonferronni correction. (0.06 MB DOC) [file pcbi.1000550.s010.doc]

**Table S5. Degrees of the genes in yeast PIN belonging to each functional category**

| GO biological process category | mean degree^a^ | # of proteins^b^ |
| --- | --- | --- |
| conjugation | 6.27 (***^c^) | 92 |
| cytokinesis | 5.92 (***) | 94 |
| carbohydrate metabolic process | 2.98 | 105 |
| generation of precursor metabolite energy | 3.63 | 105 |
| DNA metabolic process | 4.28 | 259 |
| transcription | 6.05 (***) | 187 |
| translation | 2.45 | 185 |
| protein folding | 3.15 | 157 |
| protein modification process | 3.96 (*) | 392 |
| amino acid and derivative metabolic process | 3.36 | 136 |
| lipid metabolic process | 2.14 | 139 |
| aromatic compound metabolic process | 2.67 | 48 |
| vitamin metabolic process | 2.48 | 56 |
| transport | 4.57 (***) | 690 |
| response to stress | 4.62 (***) | 364 |
| organelle organization and biogenesis | 4.76 (***) | 992 |
| nuclear organization and biogenesis | 8.50 (***) | 52 |
| cytoskeleton organization and biogenesis | 6.69 (***) | 198 |
| cell wall organization and biogenesis | 3.98 | 136 |
| cell cycle | 5.14 (***) | 301 |
| cell budding | 7.29 (***) | 72 |
| pseudohyphal growth | 5.23 | 51 |
| meiosis | 4.56 (***) | 108 |
| signal transduction | 5.49 (***) | 173 |
| anatomical structure morphogenesis | 6.59 (***) | 119 |
| membrane organization and biogenesis | 5.63 (***) | 152 |
| RNA metabolic process | 5.82 (***) | 516 |
| vesicle mediated transport | 5.18 (***) | 268 |
| cellular homeostasis | 3.82 | 71 |
| protein catabolic process | 4.56 (*) | 127 |
| sporulation | 3.85 | 87 |
| response to chemical stimulus | 3.99 (**) | 285 |
| ribosome biogenesis and assembly | 3.19 | 237 |
| cellular respiration | 2.15 | 60 |
| heterocycle metabolic process | 2.67 | 53 |
| cofactor metabolic process | 2.40 | 96 |
| All proteins in the yeast PIN | 3.57 | 4,153 |

1. Mean degree among the proteins contained in each functional category.
2. Number of proteins in each functional category.
3. ***, **, and * represents that a given value is significantly higher (or lower) than average degree among proteins belonging other functional categories with *P* < 0.001, *P* < 0.01, and *P* < 0.05, respectively, by the Wilcoxon rank-sum two-sample test with the Bonferronni correction.
