# Supplementary material for: Structure of Protein Interaction Networks and Their Implications on Drug Design
Source: PLoS Comput Biol. 2009 Oct 30;5(10):e1000550. doi: 10.1371/journal.pcbi.1000550 (PMC2760708; doi:10.1371/journal.pcbi.1000550)
Supplement: Table S6 — Degrees of the genes in human PIN belonging to each functional category. a. See Table S5. (0.04 MB DOC) [file pcbi.1000550.s011.doc]

**Table S6. Degrees of the genes in human PIN belonging to each functional category**

| GO biological process category | mean degree^a^ | # of proteins^a^ |
| --- | --- | --- |
| transport | 3.02 | 268 |
| multicellular organismal development | 4.80 (***^a^) | 376 |
| metabolic process | 2.47 (***) | 171 |
| catabolic process | 1.77 | 13 |
| biosyntheric process | 1.47 (*) | 19 |
| pathogenesis | 1.67 | 3 |
| cell differentiation | 5.30 (**) | 626 |
| extracellular structure organization and biogenesis | 3.60 | 10 |
| macromolecule metabolic process | 4.60 (***) | 1,034 |
| secretion | 3.63 | 40 |
| response to stimulus | 5.27 (***) | 558 |
| All proteins in the human PIN | 4.06 | 3,023 |

a. See Table S5.
