# Supplementary material for: Structure of Protein Interaction Networks and Their Implications on Drug Design
Source: PLoS Comput Biol. 2009 Oct 30;5(10):e1000550. doi: 10.1371/journal.pcbi.1000550 (PMC2760708; doi:10.1371/journal.pcbi.1000550)
Supplement: Table S7 — Middle degree proteins in human PIN and their functions. (2.79 MB DOC) [file pcbi.1000550.s012.doc]

**Table S7. Middle degree proteins in the human PIN and their functions.**

| ORF^a^ | Targets^b^ | Degree | GO Biological Process |
| --- | --- | --- | --- |
| ZNFN1A1 | NDT | 7 | transcription |
| ZNFN1A1 | NDT | 7 | regulation of transcription DNA-dependent |
| ZNFN1A1 | NDT | 7 | mesoderm development |
| ZNF655 | NDT | 6 | transcription |
| ZNF655 | NDT | 6 | regulation of transcription DNA-dependent |
| ZNF638 | NDT | 10 | RNA splicing |
| ZNF581 | NDT | 8 | transcription |
| ZNF581 | NDT | 8 | regulation of transcription DNA-dependent |
| ZNF451 | NDT | 7 | transcription |
| ZNF451 | NDT | 7 | regulation of transcription DNA-dependent |
| ZNF426 | NDT | 8 | transcription |
| ZNF426 | NDT | 8 | regulation of transcription DNA-dependent |
| ZNF417 | NDT | 7 | transcription |
| ZNF417 | NDT | 7 | regulation of transcription DNA-dependent |
| ZNF408 | NDT | 14 | transcription |
| ZNF408 | NDT | 14 | regulation of transcription DNA-dependent |
| ZNF297B | NDT | 7 | transcription |
| ZNF297B | NDT | 7 | regulation of transcription DNA-dependent |
| ZNF263 | NDT | 9 | transcription |
| ZNF263 | NDT | 9 | regulation of transcription DNA-dependent |
| ZNF250 | NDT | 14 | regulation of transcription DNA-dependent |
| ZNF250 | NDT | 14 | transcription |
| ZNF165 | NDT | 12 | transcription |
| ZNF165 | NDT | 12 | regulation of transcription DNA-dependent |
| ZFYVE9 | NDT | 17 | endocytosis |
| ZFYVE9 | NDT | 17 | transforming growth factor beta receptor complex assembly |
| ZFYVE9 | NDT | 17 | SMAD protein complex assembly |
| ZFYVE9 | NDT | 17 | SMAD protein nuclear translocation |
| ZBTB8 | NDT | 17 | transcription |
| ZBTB8 | NDT | 17 | regulation of transcription DNA-dependent |
| ZBTB16 | NDT | 25 | mesonephros development |
| ZBTB16 | NDT | 25 | transcription |
| ZBTB16 | NDT | 25 | ubiquitin cycle |
| ZBTB16 | NDT | 25 | apoptosis |
| ZBTB16 | NDT | 25 | central nervous system development |
| ZBTB16 | NDT | 25 | negative regulation of myeloid cell differentiation |
| ZBTB16 | NDT | 25 | negative regulation of transcription DNA-dependent |
| ZAP70 | DT | 14 | protein amino acid phosphorylation |
| ZAP70 | DT | 14 | immune response |
| ZAP70 | DT | 14 | signal transduction |
| ZAP70 | DT | 14 | protein kinase cascade |
| ZAP70 | DT | 14 | positive thymic T cell selection |
| ZAP70 | DT | 14 | positive regulation of T cell differentiation |
| YY1 | NDT | 6 | transcription |
| YY1 | NDT | 6 | regulation of transcription from RNA polymerase II promoter |
| YY1 | NDT | 6 | anterior |
| YY1 | NDT | 6 | camera-type eye morphogenesis |
| YWHAG | NDT | 9 | negative regulation of protein kinase activity |
| YWHAG | NDT | 9 | protein targeting |
| YWHAG | NDT | 9 | regulation of signal transduction |
| YWHAG | NDT | 9 | regulation of neuron differentiation |
| YWHAG | NDT | 9 | regulation of synaptic plasticity |
| YWHAE | NDT | 21 | intracellular signaling cascade |
| YWHAE | NDT | 21 | interspecies interaction between organisms |
| YWHAB | NDT | 18 | protein targeting |
| YWHAB | NDT | 18 | Ras protein signal transduction |
| YWHAB | NDT | 18 | activation of pro-apoptotic gene products |
| XRN2 | NDT | 12 | DNA catabolic process exonucleolytic |
| XRN2 | NDT | 12 | transcription |
| XRN2 | NDT | 12 | transcription termination |
| XRN2 | NDT | 12 | regulation of transcription DNA-dependent |
| XRN2 | NDT | 12 | mRNA processing |
| XRN2 | NDT | 12 | RNA catabolic process |
| XRN2 | NDT | 12 | spermatogenesis |
| XRN2 | NDT | 12 | cell growth |
| WWP2 | NDT | 7 | protein modification process |
| WWP2 | NDT | 7 | ubiquitin cycle |
| WWP2 | NDT | 7 | interspecies interaction between organisms |
| WWP2 | NDT | 7 | entry of virus into host cell |
| WWP1 | NDT | 7 | protein modification process |
| WWP1 | NDT | 7 | signal transduction |
| WWP1 | NDT | 7 | central nervous system development |
| WWP1 | NDT | 7 | negative regulation of transcription |
| WWP1 | NDT | 7 | protein ubiquitination |
| WWP1 | NDT | 7 | interspecies interaction between organisms |
| WWP1 | NDT | 7 | entry of virus into host cell |
| WBP11 | NDT | 7 | rRNA processing |
| WBP11 | NDT | 7 | mRNA processing |
| WBP11 | NDT | 7 | RNA splicing |
| WAS | NDT | 13 | protein complex assembly |
| WAS | NDT | 13 | defense response |
| WAS | NDT | 13 | immune response |
| WAS | NDT | 13 | blood coagulation |
| WAS | NDT | 13 | epidermis development |
| WAS | NDT | 13 | endosome transport |
| WAS | NDT | 13 | actin filament polymerization |
| WAS | NDT | 13 | actin filament-based movement |
| WAS | NDT | 13 | T cell activation |
| VTN | NDT | 7 | immune response |
| VTN | NDT | 7 | cell adhesion |
| VTN | NDT | 7 | cell-matrix adhesion |
| VIM | NDT | 22 | cell motility |
| VIM | NDT | 22 | interspecies interaction between organisms |
| VIM | NDT | 22 | intermediate filament-based process |
| VIL2 | NDT | 9 | cytoskeletal anchoring at plasma membrane |
| VIL2 | NDT | 9 | leukocyte adhesion |
| VIL2 | NDT | 9 | regulation of cell shape |
| VIL2 | NDT | 9 | membrane to membrane docking |
| VIL2 | NDT | 9 | actin filament bundle formation |
| VDP | NDT | 10 | intracellular protein transport |
| VDP | NDT | 10 | vesicle-mediated transport |
| VDP | NDT | 10 | vesicle fusion with Golgi apparatus |
| VDAC1 | NDT | 8 | anion transport |
| VDAC1 | NDT | 8 | apoptotic program |
| VDAC1 | NDT | 8 | interspecies interaction between organisms |
| VAV1 | NDT | 17 | phagocytosis |
| VAV1 | NDT | 17 | immune response |
| VAV1 | NDT | 17 | integrin-mediated signaling pathway |
| VAV1 | NDT | 17 | intracellular signaling cascade |
| VAV1 | NDT | 17 | regulation of Rho protein signal transduction |
| VAV1 | NDT | 17 | regulation of GTPase activity |
| VAV1 | NDT | 17 | positive regulation of cell adhesion |
| VAMP2 | NDT | 7 | vesicle-mediated transport |
| USP2 | NDT | 6 | ubiquitin-dependent protein catabolic process |
| USP2 | NDT | 6 | ubiquitin cycle |
| UNC119 | NDT | 6 | synaptic transmission |
| UNC119 | NDT | 6 | visual perception |
| UNC119 | NDT | 6 | phototransduction |
| UNC119 | NDT | 6 | response to stimulus |
| UBQLN1 | NDT | 25 | protein modification process |
| UBE3A | NDT | 8 | protein modification process |
| UBE3A | NDT | 8 | ubiquitin cycle |
| UBE3A | NDT | 8 | proteolysis |
| UBE3A | NDT | 8 | ubiquitin-dependent protein catabolic process |
| UBE3A | NDT | 8 | brain development |
| UBE3A | NDT | 8 | interspecies interaction between organisms |
| UBB | NDT | 7 | protein modification process |
| UBB | NDT | 7 | cell cycle |
| UBB | NDT | 7 | axon guidance |
| UBB | NDT | 7 | protein ubiquitination |
| UBB | NDT | 7 | ER-associated protein catabolic process |
| UBB | NDT | 7 | anaphase-promoting complex-dependent proteasomal ubiquitin-dependent protein catabolic process |
| UBB | NDT | 7 | long-term strengthening of neuromuscular junction |
| UBB | NDT | 7 | positive regulation of transcription |
| UBB | NDT | 7 | regulation of synaptic plasticity |
| UBB | NDT | 7 | negative regulation of ubiquitin-protein ligase activity during mitotic cell cycle |
| UBB | NDT | 7 | positive regulation of ubiquitin-protein ligase activity during mitotic cell cycle |
| U2AF2 | NDT | 16 | mRNA processing |
| U2AF2 | NDT | 16 | nuclear mRNA splicing via spliceosome |
| U2AF2 | NDT | 16 | RNA splicing |
| TYK2 | NDT | 8 | protein amino acid phosphorylation |
| TXNL2 | NDT | 6 | cell redox homeostasis |
| TUBG1 | NDT | 6 | meiotic spindle organization and biogenesis |
| TUBG1 | NDT | 6 | microtubule cytoskeleton organization and biogenesis |
| TUBG1 | NDT | 6 | protein polymerization |
| TUBB2 | DT | 12 | microtubule-based movement |
| TUBB2 | DT | 12 | mitosis |
| TUBB2 | DT | 12 | neuron differentiation |
| TUBB2 | DT | 12 | protein polymerization |
| TUBA1 | DT | 10 | microtubule-based movement |
| TUBA1 | DT | 10 | protein polymerization |
| TU3A | NDT | 15 | regulation of cell growth |
| TTRAP | NDT | 7 | cell surface receptor linked signal transduction |
| TSRC1 | NDT | 11 | positive regulation of apoptosis |
| TSG101 | NDT | 10 | regulation of cell growth |
| TSG101 | NDT | 10 | ubiquitin cycle |
| TSG101 | NDT | 10 | protein transport |
| TSG101 | NDT | 10 | ubiquitin-dependent protein catabolic process via the multivesicular body pathway |
| TSG101 | NDT | 10 | interspecies interaction between organisms |
| TSG101 | NDT | 10 | regulation of protein metabolic process |
| TSC22D4 | NDT | 27 | transcription |
| TSC22D4 | NDT | 27 | regulation of transcription DNA-dependent |
| TRIP6 | NDT | 19 | release of cytoplasmic sequestered NF-kappaB |
| TRIP6 | NDT | 19 | positive regulation of cell migration |
| TRIP6 | NDT | 19 | focal adhesion formation |
| TRIM39 | NDT | 8 | biological process |
| TRIM29 | NDT | 15 | transcription from RNA polymerase II promoter |
| TRIM23 | NDT | 11 | small GTPase mediated signal transduction |
| TRIM23 | NDT | 11 | protein ubiquitination |
| TRAF6 | NDT | 17 | protein polyubiquitination |
| TRAF6 | NDT | 17 | positive regulation of T cell cytokine production |
| TRAF6 | NDT | 17 | ubiquitin cycle |
| TRAF6 | NDT | 17 | signal transduction |
| TRAF6 | NDT | 17 | activation of NF-kappaB-inducing kinase activity |
| TRAF6 | NDT | 17 | positive regulation of interleukin-2 production |
| TRAF6 | NDT | 17 | regulation of apoptosis |
| TRAF6 | NDT | 17 | positive regulation of I-kappaB kinase |
| TRAF6 | NDT | 17 | T cell receptor signaling pathway |
| TRAF6 | NDT | 17 | positive regulation of T cell activation |
| TRAF4 | NDT | 8 | signal transduction |
| TRAF4 | NDT | 8 | multicellular organismal development |
| TRAF4 | NDT | 8 | regulation of apoptosis |
| TOLLIP | NDT | 7 | inflammatory response |
| TOLLIP | NDT | 7 | immune response |
| TOLLIP | NDT | 7 | intracellular signaling cascade |
| TOLLIP | NDT | 7 | cell-cell signaling |
| TOLLIP | NDT | 7 | phosphorylation |
| TOLLIP | NDT | 7 | leukocyte activation |
| TNNT1 | NDT | 7 | skeletal muscle contraction |
| TNNT1 | NDT | 7 | negative regulation of muscle contraction |
| TNFRSF1A | DT | 18 | prostaglandin metabolic process |
| TNFRSF1A | DT | 18 | apoptosis |
| TNFRSF1A | DT | 18 | signal transduction |
| TNFRSF1A | DT | 18 | cytokine and chemokine mediated signaling pathway |
| TNFRSF1A | DT | 18 | positive regulation of I-kappaB kinase |
| TNFRSF1A | DT | 18 | interspecies interaction between organisms |
| TNFRSF1A | DT | 18 | positive regulation of transcription from RNA polymerase II promoter |
| TNFRSF1A | DT | 18 | positive regulation of inflammatory response |
| THRA | DT | 8 | regulation of transcription DNA-dependent |
| THRA | DT | 8 | transcription from RNA polymerase II promoter |
| THAP7 | NDT | 7 | transcription |
| THAP7 | NDT | 7 | regulation of transcription DNA-dependent |
| TGM2 | DT | 9 | peptide cross-linking |
| TGM2 | DT | 9 | blood vessel remodeling |
| TGM2 | DT | 9 | induction of apoptosis |
| TGM2 | DT | 9 | isopeptide cross-linking via N6-(L-isoglutamyl)-L-lysine |
| TGM2 | DT | 9 | positive regulation of I-kappaB kinase |
| TGM2 | DT | 9 | positive regulation of cell adhesion |
| TGM2 | DT | 9 | positive regulation of smooth muscle cell proliferation |
| TGM2 | DT | 9 | positive regulation of inflammatory response |
| TGM2 | DT | 9 | protein homooligomerization |
| TGM2 | DT | 9 | elevation of cytosolic calcium ion concentration during G-protein signaling coupled to IP3 second messenger (phospholipase C activating) |
| TGFB1 | NDT | 13 | skeletal development |
| TGFB1 | NDT | 13 | response to hypoxia |
| TGFB1 | NDT | 13 | morphogenesis of a branching structure |
| TGFB1 | NDT | 13 | epithelial to mesenchymal transition |
| TGFB1 | NDT | 13 | connective tissue replacement during inflammatory response |
| TGFB1 | NDT | 13 | protein amino acid phosphorylation |
| TGFB1 | NDT | 13 | ATP biosynthetic process |
| TGFB1 | NDT | 13 | phosphate metabolic process |
| TGFB1 | NDT | 13 | induction of apoptosis |
| TGFB1 | NDT | 13 | inflammatory response |
| TGFB1 | NDT | 13 | cell cycle arrest |
| TGFB1 | NDT | 13 | SMAD protein nuclear translocation |
| TGFB1 | NDT | 13 | negative regulation of neuroblast proliferation |
| TGFB1 | NDT | 13 | cell death |
| TGFB1 | NDT | 13 | germ cell migration |
| TGFB1 | NDT | 13 | defense response to fungus incompatible interaction |
| TGFB1 | NDT | 13 | organ morphogenesis |
| TGFB1 | NDT | 13 | response to organic substance |
| TGFB1 | NDT | 13 | regulation of striated muscle development |
| TGFB1 | NDT | 13 | negative regulation of transcription |
| TGFB1 | NDT | 13 | regulation of transforming growth factor beta receptor signaling pathway |
| TGFB1 | NDT | 13 | evasion of host defenses by virus |
| TGFB1 | NDT | 13 | negative regulation of ossification |
| TGFB1 | NDT | 13 | negative regulation of cell growth |
| TGFB1 | NDT | 13 | positive regulation of exit from mitosis |
| TGFB1 | NDT | 13 | G1 |
| TGFB1 | NDT | 13 | positive regulation of collagen biosynthetic process |
| TGFB1 | NDT | 13 | positive regulation of protein amino acid dephosphorylation |
| TGFB1 | NDT | 13 | wound healing |
| TGFB1 | NDT | 13 | regulation of protein import into nucleus |
| TGFB1 | NDT | 13 | response to drug |
| TGFB1 | NDT | 13 | myelination |
| TGFB1 | NDT | 13 | regulatory T cell differentiation |
| TGFB1 | NDT | 13 | positive regulation of transcription DNA-dependent |
| TGFB1 | NDT | 13 | negative regulation of mitotic cell cycle |
| TGFB1 | NDT | 13 | positive regulation of isotype switching to IgA isotypes |
| TGFB1 | NDT | 13 | lymph node development |
| TGFB1 | NDT | 13 | positive regulation of epithelial cell proliferation |
| TGFB1 | NDT | 13 | negative regulation of epithelial cell proliferation |
| TGFB1 | NDT | 13 | negative regulation of phagocytosis |
| TGFB1 | NDT | 13 | negative regulation of immune response |
| TGFB1 | NDT | 13 | positive regulation of NF-kappaB transcription factor activity |
| TGFB1 | NDT | 13 | regulation of DNA binding |
| TGFB1 | NDT | 13 | negative regulation of release of sequestered calcium ion into cytosol |
| TCF4 | NDT | 7 | negative regulation of transcription from RNA polymerase II promoter |
| TCF4 | NDT | 7 | positive regulation of transcription DNA-dependent |
| TCF4 | NDT | 7 | blood vessel development |
| TCF4 | NDT | 7 | transcription |
| TCF4 | NDT | 7 | regulation of transcription from RNA polymerase II promoter |
| TCF4 | NDT | 7 | anti-apoptosis |
| TCF4 | NDT | 7 | cell cycle arrest |
| TCF4 | NDT | 7 | cell proliferation |
| TCF4 | NDT | 7 | pancreas development |
| TCF4 | NDT | 7 | positive regulation of insulin secretion |
| TCF4 | NDT | 7 | regulation of hormone metabolic process |
| TCF4 | NDT | 7 | glucose homeostasis |
| TCF4 | NDT | 7 | fat cell differentiation |
| TCF4 | NDT | 7 | myoblast cell fate commitment |
| TCF4 | NDT | 7 | Wnt receptor signaling pathway through beta-catenin |
| TCEB3B | NDT | 11 | regulation of transcription DNA-dependent |
| TCEB3B | NDT | 11 | transcription from RNA polymerase II promoter |
| TADA3L | NDT | 6 | transcription |
| TADA3L | NDT | 6 | regulation of transcription from RNA polymerase II promoter |
| SYK | DT | 20 | serotonin secretion |
| SYK | DT | 20 | protein complex assembly |
| SYK | DT | 20 | leukocyte adhesion |
| SYK | DT | 20 | enzyme linked receptor protein signaling pathway |
| SYK | DT | 20 | integrin-mediated signaling pathway |
| SYK | DT | 20 | intracellular signaling cascade |
| SYK | DT | 20 | activation of JNK activity |
| SYK | DT | 20 | cell proliferation |
| SYK | DT | 20 | organ morphogenesis |
| SYK | DT | 20 | leukotriene biosynthetic process |
| SYK | DT | 20 | neutrophil chemotaxis |
| SYK | DT | 20 | positive regulation of mast cell degranulation |
| SYK | DT | 20 | beta selection |
| SYK | DT | 20 | interspecies interaction between organisms |
| SYK | DT | 20 | positive regulation of interleukin-3 biosynthetic process |
| SYK | DT | 20 | positive regulation of granulocyte macrophage colony-stimulating factor biosynthetic process |
| SYK | DT | 20 | positive regulation of B cell differentiation |
| SYK | DT | 20 | positive regulation of gamma-delta T cell differentiation |
| SYK | DT | 20 | positive regulation of alpha-beta T cell differentiation |
| SYK | DT | 20 | positive regulation of alpha-beta T cell proliferation |
| SYK | DT | 20 | protein amino acid autophosphorylation |
| SYK | DT | 20 | positive regulation of peptidyl-tyrosine phosphorylation |
| SYK | DT | 20 | positive regulation of calcium-mediated signaling |
| SYK | DT | 20 | B cell receptor signaling pathway |
| SUMO1 | NDT | 14 | DNA repair |
| SUMO1 | NDT | 14 | protein modification process |
| SUMO1 | NDT | 14 | ubiquitin cycle |
| SUMO1 | NDT | 14 | negative regulation of transcription |
| SUMO1 | NDT | 14 | protein sumoylation |
| SUMO1 | NDT | 14 | regulation of protein localization |
| SUMO1 | NDT | 14 | negative regulation of transcription factor activity |
| SUFU | NDT | 13 | skeletal development |
| SUFU | NDT | 13 | regulation of transcription DNA-dependent |
| SUFU | NDT | 13 | proteolysis |
| SUFU | NDT | 13 | signal transduction |
| SUFU | NDT | 13 | multicellular organismal development |
| SUFU | NDT | 13 | negative regulation of cell cycle |
| STX5A | NDT | 6 | intracellular protein transport |
| STX5A | NDT | 6 | vesicle targeting |
| STX5A | NDT | 6 | retrograde transport endosome to Golgi |
| STX4A | NDT | 10 | intracellular protein transport |
| STX4A | NDT | 10 | vesicle-mediated transport |
| STX4A | NDT | 10 | neurotransmitter transport |
| STX11 | NDT | 6 | intracellular protein transport |
| STX11 | NDT | 6 | membrane fusion |
| STX11 | NDT | 6 | vesicle-mediated transport |
| STUB1 | NDT | 6 | protein polyubiquitination |
| STUB1 | NDT | 6 | protein folding |
| STUB1 | NDT | 6 | ubiquitin-dependent SMAD protein catabolic process |
| STUB1 | NDT | 6 | positive regulation of protein ubiquitination |
| STUB1 | NDT | 6 | regulation of glucocorticoid metabolic process |
| STUB1 | NDT | 6 | positive regulation of proteasomal ubiquitin-dependent protein catabolic process |
| STUB1 | NDT | 6 | protein maturation |
| STAT5A | NDT | 15 | luteinization |
| STAT5A | NDT | 15 | natural killer cell differentiation |
| STAT5A | NDT | 15 | signal transduction |
| STAT5A | NDT | 15 | JAK-STAT cascade |
| STAT5A | NDT | 15 | female pregnancy |
| STAT5A | NDT | 15 | lactation |
| STAT5A | NDT | 15 | positive regulation of cell proliferation |
| STAT5A | NDT | 15 | regulation of steroid metabolic process |
| STAT5A | NDT | 15 | cytokine and chemokine mediated signaling pathway |
| STAT5A | NDT | 15 | sequestering of lipid |
| STAT5A | NDT | 15 | regulation of cell adhesion |
| STAT5A | NDT | 15 | regulation of epithelial cell differentiation |
| STAT5A | NDT | 15 | mammary gland development |
| STAT5A | NDT | 15 | positive regulation of multicellular organism growth |
| STAT5A | NDT | 15 | positive regulation of activated T cell proliferation |
| STAT5A | NDT | 15 | T cell homeostasis |
| STAT5A | NDT | 15 | negative regulation of apoptosis |
| STAT5A | NDT | 15 | positive regulation of interleukin-2 biosynthetic process |
| STAT5A | NDT | 15 | positive regulation of B cell differentiation |
| STAT5A | NDT | 15 | negative regulation of erythrocyte differentiation |
| STAT5A | NDT | 15 | positive regulation of survival gene product expression |
| STAT5A | NDT | 15 | positive regulation of mitotic cell cycle |
| STAT5A | NDT | 15 | positive regulation of transcription from RNA polymerase II promoter |
| STAT5A | NDT | 15 | development of secondary female sexual characteristics |
| STAT5A | NDT | 15 | development of secondary male sexual characteristics |
| STAT5A | NDT | 15 | positive regulation of inflammatory response |
| STAT3 | NDT | 25 | negative regulation of transcription from RNA polymerase II promoter |
| STAT3 | NDT | 25 | cell motility |
| STAT3 | NDT | 25 | signal transduction |
| STAT3 | NDT | 25 | JAK-STAT cascade |
| STAT3 | NDT | 25 | nervous system development |
| STAT3 | NDT | 25 | cytokine and chemokine mediated signaling pathway |
| STAT3 | NDT | 25 | interspecies interaction between organisms |
| STAT1 | NDT | 18 | regulation of transcription DNA-dependent |
| STAT1 | NDT | 18 | signal transduction |
| STAT1 | NDT | 18 | transcription from RNA polymerase II promoter |
| STAT1 | NDT | 18 | caspase activation |
| STAT1 | NDT | 18 | I-kappaB kinase |
| STAT1 | NDT | 18 | tyrosine phosphorylation of STAT protein |
| STAT1 | NDT | 18 | response to virus |
| STAT1 | NDT | 18 | interspecies interaction between organisms |
| STAM2 | NDT | 7 | intracellular protein transport |
| SSSCA1 | NDT | 12 | cell cycle |
| SSSCA1 | NDT | 12 | mitosis |
| SSSCA1 | NDT | 12 | cell division |
| SQSTM1 | NDT | 9 | ubiquitin-dependent protein catabolic process |
| SQSTM1 | NDT | 9 | apoptosis |
| SQSTM1 | NDT | 9 | response to stress |
| SQSTM1 | NDT | 9 | immune response |
| SQSTM1 | NDT | 9 | intracellular signaling cascade |
| SQSTM1 | NDT | 9 | protein localization |
| SQSTM1 | NDT | 9 | endosome transport |
| SQSTM1 | NDT | 9 | cell differentiation |
| SQSTM1 | NDT | 9 | regulation of I-kappaB kinase |
| SQSTM1 | NDT | 9 | positive regulation of transcription from RNA polymerase II promoter |
| SPRY2 | NDT | 8 | cell-cell signaling |
| SPRY2 | NDT | 8 | multicellular organismal development |
| SPRY2 | NDT | 8 | sensory perception of sound |
| SPRY2 | NDT | 8 | organ morphogenesis |
| SPRY2 | NDT | 8 | regulation of signal transduction |
| SPRY2 | NDT | 8 | lung development |
| SPRY2 | NDT | 8 | inner ear morphogenesis |
| SPRY2 | NDT | 8 | negative regulation of MAP kinase activity |
| SPRY2 | NDT | 8 | cell fate commitment |
| SPRY2 | NDT | 8 | branching morphogenesis of a tube |
| SPP1 | NDT | 6 | ossification |
| SPP1 | NDT | 6 | cell adhesion |
| SPOP | NDT | 6 | mRNA processing |
| SPOP | NDT | 6 | ubiquitin cycle |
| SPG21 | NDT | 11 | antigen receptor-mediated signaling pathway |
| SPARC | DT | 6 | ossification |
| SPARC | DT | 6 | transmembrane receptor protein tyrosine kinase signaling pathway |
| SNRPE | NDT | 8 | mRNA metabolic process |
| SNRPE | NDT | 8 | spliceosome assembly |
| SNRPE | NDT | 8 | spliceosomal snRNP biogenesis |
| SNRPE | NDT | 8 | RNA splicing |
| SNIP1 | NDT | 12 | regulation of transcription DNA-dependent |
| SNIP1 | NDT | 12 | I-kappaB kinase |
| SNAP25 | DT | 6 | neurotransmitter uptake |
| SNAP25 | DT | 6 | synaptic transmission |
| SNAP25 | DT | 6 | neurotransmitter secretion |
| SNAP25 | DT | 6 | synaptic vesicle docking during exocytosis |
| SNAP25 | DT | 6 | regulation of insulin secretion |
| SNAP23 | NDT | 14 | post-Golgi vesicle-mediated transport |
| SNAP23 | NDT | 14 | vesicle targeting |
| SNAP23 | NDT | 14 | membrane fusion |
| SNAP23 | NDT | 14 | protein transport |
| SMN1 | NDT | 16 | spliceosome assembly |
| SMN1 | NDT | 16 | spliceosomal snRNP biogenesis |
| SMN1 | NDT | 16 | mRNA processing |
| SMN1 | NDT | 16 | RNA splicing |
| SMARCD1 | NDT | 7 | regulation of transcription from RNA polymerase II promoter |
| SMARCD1 | NDT | 7 | chromatin-mediated maintenance of transcription |
| SMAD5 | NDT | 13 | transcription |
| SMAD5 | NDT | 13 | regulation of transcription DNA-dependent |
| SMAD5 | NDT | 13 | signal transduction |
| SMAD5 | NDT | 13 | transforming growth factor beta receptor signaling pathway |
| SMAD5 | NDT | 13 | embryonic pattern specification |
| SMAD5 | NDT | 13 | erythrocyte differentiation |
| SMAD5 | NDT | 13 | BMP signaling pathway |
| SMAD5 | NDT | 13 | positive regulation of transcription from RNA polymerase II promoter |
| SMAD1 | NDT | 21 | regulation of transcription DNA-dependent |
| SMAD1 | NDT | 21 | MAPKKK cascade |
| SMAD1 | NDT | 21 | transcription |
| SMAD1 | NDT | 21 | signal transduction |
| SMAD1 | NDT | 21 | transforming growth factor beta receptor signaling pathway |
| SMAD1 | NDT | 21 | gamete generation |
| SMAD1 | NDT | 21 | negative regulation of cell proliferation |
| SMAD1 | NDT | 21 | embryonic pattern specification |
| SMAD1 | NDT | 21 | BMP signaling pathway |
| SMAD1 | NDT | 21 | midbrain development |
| SMAD1 | NDT | 21 | hindbrain development |
| SMAD1 | NDT | 21 | homeostatic process |
| SMAD1 | NDT | 21 | positive regulation of transcription from RNA polymerase II promoter |
| SKP2 | NDT | 10 | G1 |
| SKP2 | NDT | 10 | ubiquitin cycle |
| SKP2 | NDT | 10 | cell proliferation |
| SKP1A | NDT | 14 | ubiquitin-dependent protein catabolic process |
| SKP1A | NDT | 14 | ubiquitin cycle |
| SKP1A | NDT | 14 | positive regulation of ubiquitin-protein ligase activity during mitotic cell cycle |
| SIAH1 | NDT | 12 | proteolysis |
| SIAH1 | NDT | 12 | ubiquitin-dependent protein catabolic process |
| SIAH1 | NDT | 12 | ubiquitin cycle |
| SIAH1 | NDT | 12 | apoptosis |
| SIAH1 | NDT | 12 | cell cycle |
| SIAH1 | NDT | 12 | multicellular organismal development |
| SIAH1 | NDT | 12 | spermatogenesis |
| SIAH1 | NDT | 12 | nervous system development |
| SIAH1 | NDT | 12 | axon guidance |
| SIAH1 | NDT | 12 | anatomical structure morphogenesis |
| SIAH1 | NDT | 12 | cell differentiation |
| SIAH1 | NDT | 12 | protein catabolic process |
| SHC1 | NDT | 26 | intracellular signaling cascade |
| SHC1 | NDT | 26 | activation of MAPK activity |
| SHC1 | NDT | 26 | regulation of cell growth |
| SHC1 | NDT | 26 | epidermal growth factor receptor signaling pathway |
| SHC1 | NDT | 26 | regulation of epidermal growth factor receptor activity |
| SHC1 | NDT | 26 | Ras protein signal transduction |
| SHC1 | NDT | 26 | positive regulation of cell proliferation |
| SHC1 | NDT | 26 | positive regulation of mitosis |
| SH3KBP1 | NDT | 8 | endocytosis |
| SH3KBP1 | NDT | 8 | apoptosis |
| SH3KBP1 | NDT | 8 | cell-cell signaling |
| SGTA | NDT | 14 | biological process |
| SGTA | NDT | 14 | interspecies interaction between organisms |
| SFRS1 | NDT | 7 | nuclear mRNA splicing via spliceosome |
| SFRS1 | NDT | 7 | mRNA splice site selection |
| SFRS1 | NDT | 7 | RNA splicing |
| SFN | NDT | 15 | nucleobase nucleoside nucleotide and nucleic acid metabolic process |
| SFN | NDT | 15 | nucleotide metabolic process |
| SFN | NDT | 15 | regulation of cyclin-dependent protein kinase activity |
| SFN | NDT | 15 | release of cytochrome c from mitochondria |
| SFN | NDT | 15 | negative regulation of protein kinase activity |
| SFN | NDT | 15 | signal transduction |
| SFN | NDT | 15 | cell proliferation |
| SFN | NDT | 15 | DNA damage response signal transduction resulting in induction of apoptosis |
| SFN | NDT | 15 | apoptotic program |
| SFN | NDT | 15 | keratinocyte differentiation |
| SFN | NDT | 15 | negative regulation of caspase activity |
| SFN | NDT | 15 | skin development |
| SF1 | NDT | 8 | transcription |
| SF1 | NDT | 8 | regulation of transcription from RNA polymerase II promoter |
| SF1 | NDT | 8 | signal transduction |
| SF1 | NDT | 8 | cell-cell signaling |
| SF1 | NDT | 8 | primary sex determination |
| SF1 | NDT | 8 | male gonad development |
| SF1 | NDT | 8 | regulation of steroid biosynthetic process |
| SF1 | NDT | 8 | biological process |
| SF1 | NDT | 8 | spliceosome assembly |
| SF1 | NDT | 8 | nuclear mRNA 3'-splice site recognition |
| SF1 | NDT | 8 | regulation of transcription DNA-dependent |
| SF1 | NDT | 8 | RNA splicing |
| SF1 | NDT | 8 | negative regulation of smooth muscle cell proliferation |
| SET | NDT | 10 | nucleosome assembly |
| SET | NDT | 10 | DNA replication |
| SET | NDT | 10 | nucleosome disassembly |
| SET | NDT | 10 | nucleocytoplasmic transport |
| SET | NDT | 10 | negative regulation of histone acetylation |
| SERPINA5 | NDT | 7 | spermatogenesis |
| SERPINA5 | NDT | 7 | fusion of sperm to egg plasma membrane |
| SERPINA5 | NDT | 7 | negative regulation of proteolysis |
| SDCBP2 | NDT | 7 | intracellular signaling cascade |
| SDCBP2 | NDT | 7 | nervous system development |
| SDCBP2 | NDT | 7 | intracellular transport |
| SCAND1 | NDT | 6 | regulation of transcription DNA-dependent |
| SAT | DT | 11 | metabolic process |
| S100B | NDT | 10 | axonogenesis |
| S100B | NDT | 10 | central nervous system development |
| S100B | NDT | 10 | learning and |
| S100B | NDT | 10 | cell proliferation |
| S100A6 | DT | 6 | cell cycle |
| S100A6 | DT | 6 | signal transduction |
| S100A6 | DT | 6 | axonogenesis |
| S100A6 | DT | 6 | cell proliferation |
| S100A6 | DT | 6 | positive regulation of fibroblast proliferation |
| S100A1 | NDT | 11 | intracellular signaling cascade |
| S100A1 | NDT | 11 | regulation of heart contraction |
| RXRB | DT | 8 | transcription |
| RXRB | DT | 8 | regulation of transcription DNA-dependent |
| RXRA | DT | 27 | transcription |
| RXRA | DT | 27 | regulation of transcription DNA-dependent |
| RXRA | DT | 27 | vitamin metabolic process |
| RXRA | DT | 27 | cholesterol metabolic process |
| RXRA | DT | 27 | response to retinoic acid |
| RXRA | DT | 27 | interspecies interaction between organisms |
| RXRA | DT | 27 | positive regulation of transcription from RNA polymerase II promoter |
| RXRA | DT | 27 | retinoic acid receptor signaling pathway |
| RUNX1T1 | NDT | 13 | regulation of transcription DNA-dependent |
| RUNX1T1 | NDT | 13 | generation of precursor metabolites and energy |
| RUNX1T1 | NDT | 13 | transcription |
| RTN4 | NDT | 11 | negative regulation of anti-apoptosis |
| RTN4 | NDT | 11 | regulation of cell migration |
| RTN4 | NDT | 11 | negative regulation of axon extension |
| RTN4 | NDT | 11 | regulation of apoptosis |
| RTN3 | NDT | 8 | apoptosis |
| RTN3 | NDT | 8 | response to stress |
| RTN3 | NDT | 8 | vesicle-mediated transport |
| RTN3 | NDT | 8 | interspecies interaction between organisms |
| RPIA | NDT | 6 | pentose-phosphate shunt non-oxidative branch |
| RPA2 | NDT | 6 | nucleotide-excision repair DNA damage removal |
| RPA2 | NDT | 6 | DNA-dependent DNA replication |
| RPA2 | NDT | 6 | nucleotide-excision repair DNA gap filling |
| RNF11 | NDT | 25 | protein ubiquitination during ubiquitin-dependent protein catabolic process |
| RIPK2 | NDT | 10 | protein amino acid phosphorylation |
| RIPK2 | NDT | 10 | inflammatory response |
| RIPK2 | NDT | 10 | signal transduction |
| RIPK2 | NDT | 10 | T cell proliferation |
| RIPK2 | NDT | 10 | positive regulation of apoptosis |
| RIPK2 | NDT | 10 | positive regulation of I-kappaB kinase |
| RIPK2 | NDT | 10 | positive regulation of NF-kappaB transcription factor activity |
| RHOA | DT | 14 | small GTPase mediated signal transduction |
| RHOA | DT | 14 | Rho protein signal transduction |
| RHOA | DT | 14 | actin cytoskeleton organization and biogenesis |
| RHOA | DT | 14 | positive regulation of NF-kappaB import into nucleus |
| RHOA | DT | 14 | positive regulation of I-kappaB kinase |
| RGS4 | NDT | 6 | inactivation of MAPK activity |
| RGS4 | NDT | 6 | regulation of G-protein coupled receptor protein signaling pathway |
| RGS4 | NDT | 6 | negative regulation of signal transduction |
| RGS2 | NDT | 6 | cell cycle |
| RGS2 | NDT | 6 | transmembrane receptor protein tyrosine kinase signaling pathway |
| RGS2 | NDT | 6 | regulation of G-protein coupled receptor protein signaling pathway |
| RGS2 | NDT | 6 | negative regulation of signal transduction |
| RFXDC1 | NDT | 7 | regulation of transcription DNA-dependent |
| RET | NDT | 8 | regulation of protein amino acid phosphorylation |
| RET | NDT | 8 | signal transduction |
| RET | NDT | 8 | protein amino acid phosphorylation |
| RET | NDT | 8 | homophilic cell adhesion |
| RET | NDT | 8 | posterior midgut development |
| RELA | NDT | 25 | regulation of transcription DNA-dependent |
| RELA | NDT | 25 | positive regulation of I-kappaB kinase |
| RELA | NDT | 25 | anti-apoptosis |
| RELA | NDT | 25 | inflammatory response |
| RELA | NDT | 25 | cellular defense response |
| RELA | NDT | 25 | response to organic substance |
| RELA | NDT | 25 | response to UV-B |
| RELA | NDT | 25 | cytokine and chemokine mediated signaling pathway |
| RELA | NDT | 25 | positive regulation of NF-kappaB transcription factor activity |
| RELA | NDT | 25 | defense response to virus |
| RBMX | NDT | 11 | nuclear mRNA splicing via spliceosome |
| RBMX | NDT | 11 | biological process |
| RBMX | NDT | 11 | RNA splicing |
| RBL1 | NDT | 11 | transcription |
| RBL1 | NDT | 11 | regulation of transcription DNA-dependent |
| RBL1 | NDT | 11 | chromatin modification |
| RBL1 | NDT | 11 | regulation of lipid kinase activity |
| RBL1 | NDT | 11 | negative regulation of cell cycle |
| RBBP8 | NDT | 12 | cell cycle checkpoint |
| RBBP8 | NDT | 12 | DNA repair |
| RBBP8 | NDT | 12 | regulation of transcription from RNA polymerase II promoter |
| RASA1 | NDT | 14 | cytokinesis |
| RASA1 | NDT | 14 | vasculogenesis |
| RASA1 | NDT | 14 | negative regulation of cell-matrix adhesion |
| RASA1 | NDT | 14 | intracellular signaling cascade |
| RASA1 | NDT | 14 | regulation of cell shape |
| RASA1 | NDT | 14 | embryonic development |
| RASA1 | NDT | 14 | regulation of actin filament polymerization |
| RASA1 | NDT | 14 | negative regulation of neuron apoptosis |
| RASA1 | NDT | 14 | positive regulation of anti-apoptosis |
| RASA1 | NDT | 14 | regulation of small GTPase mediated signal transduction |
| RASA1 | NDT | 14 | regulation of RNA metabolic process |
| RARA | DT | 17 | transcription |
| RARA | DT | 17 | regulation of transcription DNA-dependent |
| RARA | DT | 17 | signal transduction |
| RARA | DT | 17 | response to retinoic acid |
| RARA | DT | 17 | retinoic acid receptor signaling pathway |
| RAP1GDS1 | NDT | 7 | biological process |
| RAN | NDT | 10 | DNA metabolic process |
| RAN | NDT | 10 | RNA export from nucleus |
| RAN | NDT | 10 | protein export from nucleus |
| RAN | NDT | 10 | intracellular protein transport |
| RAN | NDT | 10 | nucleocytoplasmic transport |
| RAN | NDT | 10 | mitotic spindle organization and biogenesis |
| RAN | NDT | 10 | mitosis |
| RAN | NDT | 10 | signal transduction |
| RAN | NDT | 10 | small GTPase mediated signal transduction |
| RAN | NDT | 10 | androgen receptor signaling pathway |
| RAN | NDT | 10 | interspecies interaction between organisms |
| RAN | NDT | 10 | positive regulation of transcription DNA-dependent |
| RAF1 | NDT | 26 | protein amino acid phosphorylation |
| RAF1 | NDT | 26 | apoptosis |
| RAF1 | NDT | 26 | cytoskeleton organization and biogenesis |
| RAF1 | NDT | 26 | Ras protein signal transduction |
| RAF1 | NDT | 26 | cell proliferation |
| RAF1 | NDT | 26 | nerve growth factor receptor signaling pathway |
| RAD9A | NDT | 8 | cell cycle checkpoint |
| RAD9A | NDT | 8 | DNA replication checkpoint |
| RAD9A | NDT | 8 | DNA damage checkpoint |
| RAD9A | NDT | 8 | DNA replication |
| RAD9A | NDT | 8 | DNA repair |
| RAD9A | NDT | 8 | positive regulation of apoptosis |
| RAD54B | NDT | 14 | DNA repair |
| RAD54B | NDT | 14 | mitotic recombination |
| RAD54B | NDT | 14 | meiotic recombination |
| RAD23A | NDT | 9 | nucleotide-excision repair |
| RAD23A | NDT | 9 | protein modification process |
| RAD23A | NDT | 9 | proteasomal ubiquitin-dependent protein catabolic process |
| RAD23A | NDT | 9 | interspecies interaction between organisms |
| RAD17 | NDT | 6 | DNA replication checkpoint |
| RAD17 | NDT | 6 | DNA damage checkpoint |
| RAD17 | NDT | 6 | DNA repair |
| RAD17 | NDT | 6 | cell cycle |
| RAD17 | NDT | 6 | mitotic cell cycle checkpoint |
| RAD17 | NDT | 6 | negative regulation of DNA replication |
| RAD17 | NDT | 6 | regulation of phosphorylation |
| RAC2 | NDT | 8 | small GTPase mediated signal transduction |
| RAC2 | NDT | 8 | chemotaxis |
| RAC2 | NDT | 8 | signal transduction |
| RAC2 | NDT | 8 | positive regulation of cell proliferation |
| RAC2 | NDT | 8 | regulation of hydrogen peroxide metabolic process |
| RAC2 | NDT | 8 | cell projection biogenesis |
| RAC2 | NDT | 8 | actin cytoskeleton organization and biogenesis |
| RAC2 | NDT | 8 | regulation of respiratory burst |
| RAC1 | NDT | 21 | small GTPase mediated signal transduction |
| RAC1 | NDT | 21 | cell motility |
| RAC1 | NDT | 21 | inflammatory response |
| RAC1 | NDT | 21 | cell adhesion |
| RAC1 | NDT | 21 | anatomical structure morphogenesis |
| RAC1 | NDT | 21 | regulation of hydrogen peroxide metabolic process |
| RAC1 | NDT | 21 | lamellipodium biogenesis |
| RAC1 | NDT | 21 | actin filament polymerization |
| RAC1 | NDT | 21 | ruffle organization and biogenesis |
| RAC1 | NDT | 21 | positive regulation of Rho protein signal transduction |
| RAC1 | NDT | 21 | negative regulation of receptor-mediated endocytosis |
| RAC1 | NDT | 21 | localization within membrane |
| RAC1 | NDT | 21 | regulation of respiratory burst |
| RAB5A | NDT | 6 | endocytosis |
| RAB5A | NDT | 6 | small GTPase mediated signal transduction |
| RAB5A | NDT | 6 | protein transport |
| PTPN6 | NDT | 19 | protein amino acid dephosphorylation |
| PTPN6 | NDT | 19 | apoptosis |
| PTPN6 | NDT | 19 | G-protein coupled receptor protein signaling pathway |
| PTPN6 | NDT | 19 | intracellular signaling cascade |
| PTPN6 | NDT | 19 | response to wounding |
| PTPN11 | DT | 19 | protein amino acid dephosphorylation |
| PTPN11 | DT | 19 | signal transduction |
| PTPN11 | DT | 19 | sensory perception of sound |
| PTPN1 | DT | 8 | protein amino acid dephosphorylation |
| PTPN1 | DT | 8 | signal transduction |
| PTPN1 | DT | 8 | insulin receptor signaling pathway |
| PTMA | NDT | 12 | biological process |
| PTMA | NDT | 12 | transcription |
| PTK2 | DT | 23 | protein amino acid phosphorylation |
| PTK2 | DT | 23 | signal complex assembly |
| PTK2 | DT | 23 | integrin-mediated signaling pathway |
| PSMF1 | NDT | 13 | ubiquitin-dependent protein catabolic process |
| PSMF1 | NDT | 13 | anaphase-promoting complex-dependent proteasomal ubiquitin-dependent protein catabolic process |
| PSMF1 | NDT | 13 | negative regulation of ubiquitin-protein ligase activity during mitotic cell cycle |
| PSMF1 | NDT | 13 | positive regulation of ubiquitin-protein ligase activity during mitotic cell cycle |
| PSME3 | NDT | 8 | anaphase-promoting complex-dependent proteasomal ubiquitin-dependent protein catabolic process |
| PSME3 | NDT | 8 | negative regulation of ubiquitin-protein ligase activity during mitotic cell cycle |
| PSME3 | NDT | 8 | positive regulation of ubiquitin-protein ligase activity during mitotic cell cycle |
| PSMA1 | NDT | 12 | ubiquitin-dependent protein catabolic process |
| PSMA1 | NDT | 12 | anaphase-promoting complex-dependent proteasomal ubiquitin-dependent protein catabolic process |
| PSMA1 | NDT | 12 | negative regulation of ubiquitin-protein ligase activity during mitotic cell cycle |
| PSMA1 | NDT | 12 | positive regulation of ubiquitin-protein ligase activity during mitotic cell cycle |
| PSEN2 | NDT | 7 | intracellular signaling cascade |
| PSEN2 | NDT | 7 | membrane protein ectodomain proteolysis |
| PSEN2 | NDT | 7 | chromosome segregation |
| PSEN2 | NDT | 7 | Notch receptor processing |
| PSEN2 | NDT | 7 | apoptotic program |
| PSEN2 | NDT | 7 | protein processing |
| PSEN2 | NDT | 7 | amyloid precursor protein catabolic process |
| PSEN2 | NDT | 7 | positive regulation of catalytic activity |
| PSEN1 | NDT | 12 | membrane protein ectodomain proteolysis |
| PSEN1 | NDT | 12 | apoptosis |
| PSEN1 | NDT | 12 | anti-apoptosis |
| PSEN1 | NDT | 12 | chromosome segregation |
| PSEN1 | NDT | 12 | Notch receptor processing |
| PSEN1 | NDT | 12 | intracellular signaling cascade |
| PSEN1 | NDT | 12 | cell-cell adhesion |
| PSEN1 | NDT | 12 | protein processing |
| PSEN1 | NDT | 12 | endoplasmic reticulum calcium ion homeostasis |
| PSEN1 | NDT | 12 | regulation of phosphorylation |
| PSEN1 | NDT | 12 | amyloid precursor protein catabolic process |
| PSEN1 | NDT | 12 | positive regulation of catalytic activity |
| PSCD2 | DT | 6 | endocytosis |
| PSCD2 | DT | 6 | actin cytoskeleton organization and biogenesis |
| PSCD2 | DT | 6 | regulation of ARF protein signal transduction |
| PRTFDC1 | NDT | 7 | purine ribonucleoside salvage |
| PRTFDC1 | NDT | 7 | nucleoside metabolic process |
| PRKCZ | NDT | 16 | intracellular signaling cascade |
| PRKCZ | NDT | 16 | protein amino acid phosphorylation |
| PRKCZ | NDT | 16 | anti-apoptosis |
| PRKCI | NDT | 6 | protein amino acid phosphorylation |
| PRKCI | NDT | 6 | protein targeting to membrane |
| PRKCI | NDT | 6 | cytoskeleton organization and biogenesis |
| PRKCI | NDT | 6 | actin filament organization |
| PRKCI | NDT | 6 | intracellular signaling cascade |
| PRKCI | NDT | 6 | membrane organization and biogenesis |
| PRKCI | NDT | 6 | vesicle-mediated transport |
| PRKCI | NDT | 6 | establishment of apical |
| PRKCI | NDT | 6 | eye photoreceptor cell development |
| PRKCI | NDT | 6 | establishment and |
| PRKCI | NDT | 6 | cell-cell junction assembly and maintenance |
| PRKCI | NDT | 6 | secretion |
| PRKCI | NDT | 6 | Golgi vesicle budding |
| PRKCB1 | NDT | 13 | protein amino acid phosphorylation |
| PRKCB1 | NDT | 13 | intracellular signaling cascade |
| PRKAR1A | NDT | 6 | regulation of protein amino acid phosphorylation |
| PRKAR1A | NDT | 6 | regulation of transcription from RNA polymerase II promoter |
| PRKAR1A | NDT | 6 | hormone-mediated signaling |
| PRKAR1A | NDT | 6 | signal transduction |
| PPP3CA | NDT | 9 | protein amino acid dephosphorylation |
| PPP1CA | NDT | 15 | carbohydrate metabolic process |
| PPP1CA | NDT | 15 | glycogen metabolic process |
| PPP1CA | NDT | 15 | protein amino acid dephosphorylation |
| PPP1CA | NDT | 15 | cell cycle |
| PPP1CA | NDT | 15 | cell division |
| PPFIA1 | NDT | 6 | cell-matrix adhesion |
| PPFIA1 | NDT | 6 | signal transduction |
| PPARG | DT | 13 | transcription |
| PPARG | DT | 13 | regulation of transcription DNA-dependent |
| PPARG | DT | 13 | negative regulation of transcription from RNA polymerase II promoter |
| PPARG | DT | 13 | placenta development |
| PPARG | DT | 13 | lipid metabolic process |
| PPARG | DT | 13 | signal transduction |
| PPARG | DT | 13 | response to nutrient |
| PPARG | DT | 13 | regulation of blood pressure |
| PPARG | DT | 13 | long-chain fatty acid transport |
| PPARG | DT | 13 | monocyte differentiation |
| PPARG | DT | 13 | epithelial cell differentiation |
| PPARG | DT | 13 | cellular response to insulin stimulus |
| PPARG | DT | 13 | response to lipid |
| PPARG | DT | 13 | glucose homeostasis |
| PPARG | DT | 13 | lipoprotein transport |
| PPARG | DT | 13 | innate immune response |
| PPARG | DT | 13 | cell fate commitment |
| PPARG | DT | 13 | positive regulation of fat cell differentiation |
| PPARG | DT | 13 | low-density lipoprotein receptor biosynthetic process |
| PPARG | DT | 13 | positive regulation of transcription from RNA polymerase II promoter |
| PPARG | DT | 13 | cell maturation |
| PPARG | DT | 13 | white fat cell differentiation |
| PPARG | DT | 13 | lipid homeostasis |
| PPARG | DT | 13 | response to low density lipoprotein stimulus |
| PPARA | DT | 11 | regulation of transcription DNA-dependent |
| PPARA | DT | 11 | transcription |
| PPARA | DT | 11 | response to hypoxia |
| PPARA | DT | 11 | lipid metabolic process |
| PPARA | DT | 11 | epidermis development |
| PPARA | DT | 11 | fatty acid transport |
| PPARA | DT | 11 | regulation of fatty acid metabolic process |
| PPARA | DT | 11 | positive regulation of fatty acid beta-oxidation |
| PPARA | DT | 11 | positive regulation of transcription |
| PPARA | DT | 11 | positive regulation of transcription from RNA polymerase II promoter |
| POU2F1 | NDT | 6 | regulation of transcription DNA-dependent |
| POU2F1 | NDT | 6 | negative regulation of transcription |
| POM121 | NDT | 13 | protein transport |
| POM121 | NDT | 13 | mRNA transport |
| POM121 | NDT | 13 | intracellular protein transport across a membrane |
| POLD2 | NDT | 6 | DNA replication |
| POLD2 | NDT | 6 | nucleotide-excision repair DNA gap filling |
| PML | NDT | 22 | DNA repair |
| PML | NDT | 22 | induction of apoptosis |
| PML | NDT | 22 | response to ionizing radiation |
| PML | NDT | 22 | response to hypoxia |
| PML | NDT | 22 | positive regulation of defense response to virus by host |
| PML | NDT | 22 | regulation of transcription DNA-dependent |
| PML | NDT | 22 | protein complex assembly |
| PML | NDT | 22 | protein targeting |
| PML | NDT | 22 | DNA damage response signal transduction by p53 class mediator resulting in cell cycle arrest |
| PML | NDT | 22 | cell cycle arrest |
| PML | NDT | 22 | DNA damage response signal transduction resulting in induction of apoptosis |
| PML | NDT | 22 | negative regulation of transcription |
| PML | NDT | 22 | negative regulation of angiogenesis |
| PML | NDT | 22 | negative regulation of cell growth |
| PML | NDT | 22 | PML body organization and biogenesis |
| PML | NDT | 22 | positive regulation of histone deacetylation |
| PML | NDT | 22 | negative regulation of translation in response to oxidative stress |
| PML | NDT | 22 | DNA damage response signal transduction by p53 class mediator resulting in induction of apoptosis |
| PML | NDT | 22 | interspecies interaction between organisms |
| PML | NDT | 22 | negative regulation of mitotic cell cycle |
| PML | NDT | 22 | protein stabilization |
| PML | NDT | 22 | maintenance of protein location in nucleus |
| PLK1 | NDT | 17 | protein amino acid phosphorylation |
| PLK1 | NDT | 17 | cell cycle |
| PLK1 | NDT | 17 | mitosis |
| PLK1 | NDT | 17 | cell proliferation |
| PLK1 | NDT | 17 | cell division |
| PLK1 | NDT | 17 | positive regulation of ubiquitin-protein ligase activity during mitotic cell cycle |
| PLDN | NDT | 9 | synaptic vesicle docking during exocytosis |
| PLDN | NDT | 9 | melanocyte differentiation |
| PLDN | NDT | 9 | pigmentation |
| PLCG2 | NDT | 6 | lipid metabolic process |
| PLCG2 | NDT | 6 | intracellular signaling cascade |
| PLCG2 | NDT | 6 | lipid catabolic process |
| PLAT | DT | 10 | protein modification process |
| PLAT | DT | 10 | proteolysis |
| PLAT | DT | 10 | blood coagulation |
| PIN1 | NDT | 19 | protein folding |
| PIN1 | NDT | 19 | cell cycle |
| PIN1 | NDT | 19 | regulation of mitosis |
| PIAS3 | NDT | 7 | transcription |
| PIAS3 | NDT | 7 | regulation of transcription DNA-dependent |
| PIAS3 | NDT | 7 | ubiquitin cycle |
| PIAS2 | NDT | 9 | transcription |
| PIAS2 | NDT | 9 | ubiquitin cycle |
| PIAS2 | NDT | 9 | androgen receptor signaling pathway |
| PIAS2 | NDT | 9 | positive regulation of transcription DNA-dependent |
| PHC2 | NDT | 6 | multicellular organismal development |
| PHB | NDT | 6 | DNA replication |
| PHB | NDT | 6 | signal transduction |
| PHB | NDT | 6 | negative regulation of cell proliferation |
| PHB | NDT | 6 | negative regulation of transcription |
| PHB | NDT | 6 | histone deacetylation |
| PHB | NDT | 6 | regulation of apoptosis |
| PFN1 | NDT | 6 | neural tube closure |
| PFN1 | NDT | 6 | regulation of transcription from RNA polymerase II promoter |
| PFN1 | NDT | 6 | cytoskeleton organization and biogenesis |
| PFN1 | NDT | 6 | actin cytoskeleton organization and biogenesis |
| PEX5 | NDT | 7 | protein targeting to peroxisome |
| PEX5 | NDT | 7 | protein transport |
| PEX5 | NDT | 7 | protein import into peroxisome matrix translocation |
| PEX5 | NDT | 7 | protein tetramerization |
| PEX19 | NDT | 9 | protein targeting to peroxisome |
| PEX19 | NDT | 9 | peroxisome organization and biogenesis |
| PEX19 | NDT | 9 | peroxisome membrane biogenesis |
| PDPK1 | DT | 8 | protein amino acid phosphorylation |
| PDPK1 | DT | 8 | intracellular signaling cascade |
| PDPK1 | DT | 8 | insulin receptor signaling pathway |
| PDPK1 | DT | 8 | actin cytoskeleton organization and biogenesis |
| PDLIM7 | NDT | 15 | ossification |
| PDLIM7 | NDT | 15 | receptor-mediated endocytosis |
| PDLIM7 | NDT | 15 | multicellular organismal development |
| PDLIM7 | NDT | 15 | cell differentiation |
| PDGFRB | DT | 21 | signal transduction |
| PDGFRB | DT | 21 | positive regulation of cell proliferation |
| PDGFRB | DT | 21 | positive regulation of cell migration |
| PDGFRB | DT | 21 | protein amino acid autophosphorylation |
| PDGFRB | DT | 21 | platelet-derived growth factor receptor signaling pathway |
| PDGFRB | DT | 21 | regulation of peptidyl-tyrosine phosphorylation |
| PDGFRA | NDT | 6 | cell activation |
| PDGFRA | NDT | 6 | organ morphogenesis |
| PDGFRA | NDT | 6 | peptidyl-tyrosine phosphorylation |
| PDGFRA | NDT | 6 | extracellular matrix organization and biogenesis |
| PDGFRA | NDT | 6 | lung development |
| PDGFRA | NDT | 6 | positive regulation of cell migration |
| PDGFRA | NDT | 6 | male genitalia development |
| PDGFRA | NDT | 6 | odontogenesis of dentine-containing tooth |
| PDGFRA | NDT | 6 | positive regulation of DNA replication |
| PDGFRA | NDT | 6 | protein amino acid autophosphorylation |
| PDGFRA | NDT | 6 | platelet-derived growth factor receptor signaling pathway |
| PDGFRA | NDT | 6 | positive regulation of fibroblast proliferation |
| PDE6D | NDT | 10 | visual perception |
| PDE6D | NDT | 10 | response to stimulus |
| PDCD6IP | NDT | 7 | apoptosis |
| PDCD6IP | NDT | 7 | protein transport |
| PDCD6IP | NDT | 7 | interspecies interaction between organisms |
| PDCD6 | NDT | 9 | apoptosis |
| PDCD6 | NDT | 9 | induction of apoptosis by extracellular signals |
| PDCD6 | NDT | 9 | response to calcium ion |
| PCM1 | NDT | 11 | regulation of transcription DNA-dependent |
| PCM1 | NDT | 11 | transcription from RNA polymerase II promoter |
| PCM1 | NDT | 11 | negative regulation of transcription |
| PCM1 | NDT | 11 | cilium biogenesis |
| PCM1 | NDT | 11 | centrosome organization and biogenesis |
| PCBD1 | NDT | 8 | tetrahydrobiopterin biosynthetic process |
| PCBD1 | NDT | 8 | regulation of protein homodimerization activity |
| PCBD1 | NDT | 8 | positive regulation of transcription |
| PCBD1 | NDT | 8 | protein homotetramerization |
| PCBD1 | NDT | 8 | protein heterooligomerization |
| PBX2 | NDT | 9 | regulation of transcription DNA-dependent |
| PBX2 | NDT | 9 | nervous system development |
| PBX2 | NDT | 9 | brain development |
| PARK2 | NDT | 6 | protein modification process |
| PARK2 | NDT | 6 | ubiquitin cycle |
| PARK2 | NDT | 6 | central nervous system development |
| PARK2 | NDT | 6 | protein ubiquitination during ubiquitin-dependent protein catabolic process |
| PAK7 | NDT | 6 | protein amino acid phosphorylation |
| PAK7 | NDT | 6 | anti-apoptosis |
| ORC4L | NDT | 6 | DNA replication |
| ORC4L | NDT | 6 | DNA replication initiation |
| ORC2L | NDT | 10 | negative regulation of transcription from RNA polymerase II promoter |
| ORC2L | NDT | 10 | DNA replication |
| ORC2L | NDT | 10 | DNA replication initiation |
| ORC1L | NDT | 9 | DNA replication |
| ORC1L | NDT | 9 | DNA replication initiation |
| NUP62 | NDT | 9 | cell surface receptor linked signal transduction |
| NUP62 | NDT | 9 | cell death |
| NUP62 | NDT | 9 | negative regulation of cell proliferation |
| NUP62 | NDT | 9 | hormone-mediated signaling |
| NUP62 | NDT | 9 | regulation of signal transduction |
| NUP62 | NDT | 9 | protein transport |
| NUP62 | NDT | 9 | negative regulation of epidermal growth factor receptor signaling pathway |
| NUP62 | NDT | 9 | negative regulation of apoptosis |
| NUP62 | NDT | 9 | negative regulation of non-apoptotic programmed cell death |
| NUP62 | NDT | 9 | positive regulation of I-kappaB kinase |
| NUP62 | NDT | 9 | negative regulation of MAP kinase activity |
| NUP62 | NDT | 9 | positive regulation of epidermal growth factor receptor signaling pathway |
| NUP62 | NDT | 9 | positive regulation of transcription |
| NUP62 | NDT | 9 | negative regulation of Ras protein signal transduction |
| NUP62 | NDT | 9 | mRNA transport |
| NUP62 | NDT | 9 | intracellular protein transport across a membrane |
| NRIP1 | NDT | 17 | negative regulation of transcription from RNA polymerase II promoter |
| NRIP1 | NDT | 17 | ovarian follicle rupture |
| NRIP1 | NDT | 17 | transcription |
| NRIP1 | NDT | 17 | sequestering of lipid |
| NRIP1 | NDT | 17 | androgen receptor signaling pathway |
| NRIP1 | NDT | 17 | ovulation |
| NRIP1 | NDT | 17 | positive regulation of transcription from RNA polymerase II promoter |
| NR3C1 | DT | 29 | regulation of transcription DNA-dependent |
| NR3C1 | DT | 29 | transcription from RNA polymerase II promoter |
| NR3C1 | DT | 29 | signal transduction |
| NR3C1 | DT | 29 | sex determination |
| NR3C1 | DT | 29 | chromatin modification |
| NR0B2 | NDT | 7 | negative regulation of transcription from RNA polymerase II promoter |
| NR0B2 | NDT | 7 | transcription |
| NR0B2 | NDT | 7 | regulation of transcription DNA-dependent |
| NR0B2 | NDT | 7 | cholesterol metabolic process |
| NONO | NDT | 7 | DNA repair |
| NONO | NDT | 7 | DNA recombination |
| NONO | NDT | 7 | transcription |
| NONO | NDT | 7 | regulation of transcription DNA-dependent |
| NONO | NDT | 7 | mRNA processing |
| NONO | NDT | 7 | RNA splicing |
| NOL3 | NDT | 6 | mRNA processing |
| NOL3 | NDT | 6 | anti-apoptosis |
| NOL3 | NDT | 6 | RNA splicing |
| NOL3 | NDT | 6 | regulation of apoptosis |
| NMI | NDT | 14 | transcription from RNA polymerase II promoter |
| NMI | NDT | 14 | inflammatory response |
| NMI | NDT | 14 | JAK-STAT cascade |
| NME1 | DT | 9 | GTP biosynthetic process |
| NME1 | DT | 9 | UTP biosynthetic process |
| NME1 | DT | 9 | CTP biosynthetic process |
| NME1 | DT | 9 | negative regulation of cell proliferation |
| NME1 | DT | 9 | nucleotide metabolic process |
| NME1 | DT | 9 | regulation of apoptosis |
| NME1 | DT | 9 | positive regulation of DNA binding |
| NME1 | DT | 9 | negative regulation of cell cycle |
| NME1 | DT | 9 | positive regulation of epithelial cell proliferation |
| NIF3L1 | NDT | 30 | positive regulation of transcription DNA-dependent |
| NFKBIA | NDT | 12 | protein import into nucleus translocation |
| NFKBIA | NDT | 12 | apoptosis |
| NFKBIA | NDT | 12 | cytoplasmic sequestering of NF-kappaB |
| NFKBIA | NDT | 12 | lipopolysaccharide-mediated signaling pathway |
| NFKBIA | NDT | 12 | response to lipopolysaccharide |
| NFKBIA | NDT | 12 | regulation of cell proliferation |
| NFKBIA | NDT | 12 | regulation of NF-kappaB import into nucleus |
| NFKBIA | NDT | 12 | response to exogenous dsRNA |
| NFKBIA | NDT | 12 | negative regulation of DNA binding |
| NFKBIA | NDT | 12 | interspecies interaction between organisms |
| NFKBIA | NDT | 12 | negative regulation of myeloid cell differentiation |
| NFKBIA | NDT | 12 | negative regulation of Notch signaling pathway |
| NFIL3 | NDT | 7 | transcription |
| NFIL3 | NDT | 7 | regulation of transcription DNA-dependent |
| NFIL3 | NDT | 7 | transcription from RNA polymerase II promoter |
| NFIL3 | NDT | 7 | immune response |
| NFIL3 | NDT | 7 | rhythmic process |
| NFIC | NDT | 6 | DNA replication |
| NFIC | NDT | 6 | transcription |
| NFIC | NDT | 6 | regulation of transcription DNA-dependent |
| NFIC | NDT | 6 | transcription from RNA polymerase II promoter |
| NCL | NDT | 9 | angiogenesis |
| NCK2 | NDT | 16 | regulation of translation |
| NCK2 | NDT | 16 | signal transduction |
| NCK2 | NDT | 16 | signal complex assembly |
| NCK2 | NDT | 16 | epidermal growth factor receptor signaling pathway |
| NCK2 | NDT | 16 | regulation of epidermal growth factor receptor activity |
| NCK2 | NDT | 16 | negative regulation of cell proliferation |
| NCK2 | NDT | 16 | positive regulation of actin filament polymerization |
| NCK2 | NDT | 16 | positive regulation of T cell proliferation |
| NCK2 | NDT | 16 | T cell activation |
| NCK1 | NDT | 15 | regulation of translation |
| NCK1 | NDT | 15 | actin filament organization |
| NCK1 | NDT | 15 | signal complex assembly |
| NCK1 | NDT | 15 | cell migration |
| NCK1 | NDT | 15 | lamellipodium biogenesis |
| NCK1 | NDT | 15 | positive regulation of actin filament polymerization |
| NCK1 | NDT | 15 | positive regulation of T cell proliferation |
| NCK1 | NDT | 15 | T cell activation |
| NCF2 | NDT | 6 | cellular defense response |
| NCF1 | NDT | 6 | cellular defense response |
| NCF1 | NDT | 6 | cell communication |
| NAPA | NDT | 8 | intracellular protein transport |
| NAPA | NDT | 8 | intra-Golgi vesicle-mediated transport |
| NAPA | NDT | 8 | membrane fusion |
| NAPA | NDT | 8 | brain development |
| NAPA | NDT | 8 | vesicle-mediated transport |
| NAPA | NDT | 8 | neuron differentiation |
| NAPA | NDT | 8 | apical protein localization |
| MYST2 | NDT | 11 | DNA replication |
| MYST2 | NDT | 11 | transcription |
| MYST2 | NDT | 11 | regulation of transcription DNA-dependent |
| MYST2 | NDT | 11 | chromatin modification |
| MYBL2 | NDT | 7 | regulation of transcription |
| MYBL2 | NDT | 7 | regulation of transcription DNA-dependent |
| MTA1 | NDT | 6 | regulation of transcription DNA-dependent |
| MTA1 | NDT | 6 | signal transduction |
| MPHOSPH6 | NDT | 6 | M phase of mitotic cell cycle |
| MNAT1 | NDT | 7 | regulation of cyclin-dependent protein kinase activity |
| MNAT1 | NDT | 7 | nucleotide-excision repair DNA damage removal |
| MNAT1 | NDT | 7 | DNA repair |
| MNAT1 | NDT | 7 | transcription |
| MNAT1 | NDT | 7 | transcription initiation from RNA polymerase II promoter |
| MNAT1 | NDT | 7 | RNA elongation from RNA polymerase II promoter |
| MNAT1 | NDT | 7 | protein complex assembly |
| MNAT1 | NDT | 7 | cell cycle |
| MNAT1 | NDT | 7 | cell proliferation |
| MNAT1 | NDT | 7 | positive regulation of transcription from RNA polymerase II promoter |
| MMP9 | DT | 8 | skeletal development |
| MMP9 | DT | 8 | proteolysis |
| MMP9 | DT | 8 | metabolic process |
| MMP9 | DT | 8 | extracellular matrix organization and biogenesis |
| MMP9 | DT | 8 | macrophage differentiation |
| MMP9 | DT | 8 | collagen catabolic process |
| MMP9 | DT | 8 | positive regulation of apoptosis |
| MMP2 | DT | 6 | blood vessel maturation |
| MMP2 | DT | 6 | proteolysis |
| MMP2 | DT | 6 | metabolic process |
| MMP2 | DT | 6 | collagen catabolic process |
| MLH1 | NDT | 9 | mismatch repair |
| MLH1 | NDT | 9 | negative regulation of cell cycle |
| MITF | NDT | 7 | regulation of transcription DNA-dependent |
| MITF | NDT | 7 | multicellular organismal development |
| MITF | NDT | 7 | sensory perception of sound |
| MITF | NDT | 7 | melanocyte differentiation |
| MCRS1 | NDT | 15 | protein modification process |
| MCM7 | NDT | 17 | DNA replication initiation |
| MCM7 | NDT | 17 | DNA replication |
| MCM7 | NDT | 17 | transcription |
| MCM7 | NDT | 17 | regulation of transcription DNA-dependent |
| MCM7 | NDT | 17 | response to DNA damage stimulus |
| MCM7 | NDT | 17 | cell cycle |
| MCM7 | NDT | 17 | regulation of phosphorylation |
| MCM6 | NDT | 11 | DNA replication |
| MCM6 | NDT | 11 | DNA unwinding during replication |
| MCM6 | NDT | 11 | DNA replication initiation |
| MCM6 | NDT | 11 | transcription |
| MCM6 | NDT | 11 | regulation of transcription DNA-dependent |
| MCM6 | NDT | 11 | cell cycle |
| MCM5 | NDT | 7 | DNA replication initiation |
| MCM5 | NDT | 7 | cell division |
| MCM5 | NDT | 7 | DNA replication |
| MCM5 | NDT | 7 | transcription |
| MCM5 | NDT | 7 | regulation of transcription DNA-dependent |
| MCM3 | NDT | 12 | DNA replication |
| MCM3 | NDT | 12 | DNA replication initiation |
| MCM3 | NDT | 12 | transcription |
| MCM3 | NDT | 12 | regulation of transcription DNA-dependent |
| MCM3 | NDT | 12 | cell cycle |
| MCM2 | NDT | 14 | DNA replication |
| MCM2 | NDT | 14 | DNA unwinding during replication |
| MCM2 | NDT | 14 | DNA replication initiation |
| MCM2 | NDT | 14 | nucleosome assembly |
| MCM2 | NDT | 14 | transcription |
| MCM2 | NDT | 14 | regulation of transcription DNA-dependent |
| MCM2 | NDT | 14 | cell cycle |
| MCM2 | NDT | 14 | response to DNA damage stimulus |
| MCM2 | NDT | 14 | regulation of phosphorylation |
| MCM10 | NDT | 23 | DNA replication |
| MBIP | NDT | 16 | inactivation of MAPK activity during osmolarity sensing |
| MAX | NDT | 8 | regulation of transcription |
| MAX | NDT | 8 | regulation of transcription DNA-dependent |
| MAX | NDT | 8 | transcription from RNA polymerase II promoter |
| MAPK9 | NDT | 9 | protein amino acid phosphorylation |
| MAPK9 | NDT | 9 | response to stress |
| MAPK9 | NDT | 9 | JNK cascade |
| MAPK7 | NDT | 7 | protein amino acid phosphorylation |
| MAPK7 | NDT | 7 | cell cycle |
| MAPK7 | NDT | 7 | signal transduction |
| MAPK14 | DT | 20 | protein amino acid phosphorylation |
| MAPK14 | DT | 20 | cell motility |
| MAPK14 | DT | 20 | chemotaxis |
| MAPK14 | DT | 20 | response to stress |
| MAPK14 | DT | 20 | cell surface receptor linked signal transduction |
| MAPK14 | DT | 20 | protein kinase cascade |
| MAPK14 | DT | 20 | Ras protein signal transduction |
| MAFG | NDT | 6 | in utero embryonic development |
| MAFG | NDT | 6 | regulation of transcription DNA-dependent |
| MAFG | NDT | 6 | adult behavior |
| MAFG | NDT | 6 | regulation of cellular pH |
| MAFG | NDT | 6 | regulation of cell proliferation |
| MAFG | NDT | 6 | regulation of epidermal cell differentiation |
| MAD2L1 | NDT | 8 | mitosis |
| MAD2L1 | NDT | 8 | cell cycle |
| MAD2L1 | NDT | 8 | mitotic cell cycle checkpoint |
| MAD2L1 | NDT | 8 | anaphase-promoting complex-dependent proteasomal ubiquitin-dependent protein catabolic process |
| MAD2L1 | NDT | 8 | cell division |
| MAD2L1 | NDT | 8 | negative regulation of ubiquitin-protein ligase activity during mitotic cell cycle |
| MAD1L1 | NDT | 9 | mitotic metaphase |
| MAD1L1 | NDT | 9 | mitotic anaphase |
| MAD1L1 | NDT | 9 | mitotic telophase |
| MAD1L1 | NDT | 9 | cell cycle |
| MAD1L1 | NDT | 9 | mitotic cell cycle checkpoint |
| MAD1L1 | NDT | 9 | cell division |
| LZTS2 | NDT | 8 | negative regulation of cell cycle |
| LSM6 | NDT | 9 | rRNA processing |
| LSM6 | NDT | 9 | mRNA processing |
| LSM6 | NDT | 9 | tRNA processing |
| LSM6 | NDT | 9 | RNA splicing |
| LSM5 | NDT | 8 | mRNA processing |
| LSM5 | NDT | 8 | RNA splicing |
| LSM4 | NDT | 11 | mRNA processing |
| LSM4 | NDT | 11 | RNA splicing |
| LSM3 | NDT | 10 | mRNA processing |
| LSM3 | NDT | 10 | RNA splicing |
| LSM2 | NDT | 10 | nuclear mRNA splicing via spliceosome |
| LSM2 | NDT | 10 | RNA splicing |
| LSM1 | NDT | 12 | mRNA processing |
| LSM1 | NDT | 12 | RNA splicing |
| LRDD | NDT | 6 | apoptosis |
| LRDD | NDT | 6 | signal transduction |
| LPXN | NDT | 6 | protein complex assembly |
| LPXN | NDT | 6 | cell adhesion |
| LPXN | NDT | 6 | signal transduction |
| LMO3 | NDT | 16 | transcription |
| LMO3 | NDT | 16 | regulation of transcription DNA-dependent |
| LMO2 | NDT | 11 | multicellular organismal development |
| LILRB1 | NDT | 6 | immune response |
| LILRB1 | NDT | 6 | response to virus |
| LDOC1 | NDT | 25 | negative regulation of cell proliferation |
| LCP2 | NDT | 10 | immune response |
| LCP2 | NDT | 10 | transmembrane receptor protein tyrosine kinase signaling pathway |
| LCP2 | NDT | 10 | mast cell activation |
| LCP2 | NDT | 10 | cytokine secretion |
| LCK | DT | 24 | protein amino acid phosphorylation |
| LCK | DT | 24 | cellular zinc ion homeostasis |
| LCK | DT | 24 | induction of apoptosis |
| LCK | DT | 24 | caspase activation |
| LCK | DT | 24 | intracellular signaling cascade |
| LCK | DT | 24 | Ras protein signal transduction |
| LCK | DT | 24 | hemopoiesis |
| LCK | DT | 24 | T cell differentiation |
| LCK | DT | 24 | response to drug |
| LCK | DT | 24 | interspecies interaction between organisms |
| LCK | DT | 24 | positive regulation of T cell receptor signaling pathway |
| LCK | DT | 24 | positive regulation of T cell activation |
| LCK | DT | 24 | release of sequestered calcium ion into cytosol |
| LCK | DT | 24 | regulation of lymphocyte activation |
| LAT | NDT | 9 | immune response |
| LAT | NDT | 9 | integrin-mediated signaling pathway |
| LAT | NDT | 9 | intracellular signaling cascade |
| LAT | NDT | 9 | Ras protein signal transduction |
| LAT | NDT | 9 | calcium-mediated signaling |
| LAT | NDT | 9 | mast cell degranulation |
| LAT | NDT | 9 | regulation of T cell activation |
| KRT20 | NDT | 14 | apoptosis |
| KRT20 | NDT | 14 | biological process |
| KRT20 | NDT | 14 | cellular response to stress |
| KRT20 | NDT | 14 | intermediate filament organization |
| KRT20 | NDT | 14 | regulation of protein secretion |
| KRT19 | NDT | 13 | response to estrogen stimulus |
| KRT19 | NDT | 13 | interspecies interaction between organisms |
| KRT19 | NDT | 13 | sarcomere organization |
| KRT18 | NDT | 16 | cell cycle |
| KRT18 | NDT | 16 | anatomical structure morphogenesis |
| KRT18 | NDT | 16 | Golgi to plasma membrane CFTR protein transport |
| KRT18 | NDT | 16 | negative regulation of apoptosis |
| KRT18 | NDT | 16 | interspecies interaction between organisms |
| KPNA2 | NDT | 6 | protein import into nucleus |
| KPNA2 | NDT | 6 | intracellular protein transport |
| KPNA2 | NDT | 6 | regulation of DNA recombination |
| KPNA2 | NDT | 6 | M phase specific microtubule process |
| KPNA2 | NDT | 6 | G2 phase of mitotic cell cycle |
| KPNA2 | NDT | 6 | DNA metabolic process |
| KPNA2 | NDT | 6 | NLS-bearing substrate import into nucleus |
| KPNA2 | NDT | 6 | interspecies interaction between organisms |
| KNTC2 | NDT | 9 | mitotic sister chromatid segregation |
| KNTC2 | NDT | 9 | cell cycle |
| KNTC2 | NDT | 9 | spindle organization and biogenesis |
| KNTC2 | NDT | 9 | phosphoinositide-mediated signaling |
| KNTC2 | NDT | 9 | cell division |
| KLHL12 | NDT | 13 | ubiquitin cycle |
| KLHL12 | NDT | 13 | Wnt receptor signaling pathway |
| KIAA1279 | NDT | 8 | mitochondrial transport |
| KIAA1279 | NDT | 8 | multicellular organismal development |
| KIAA1279 | NDT | 8 | nervous system development |
| KIAA1279 | NDT | 8 | cell differentiation |
| KIAA1217 | NDT | 7 | multicellular organismal development |
| KIAA1217 | NDT | 7 | embryonic skeletal development |
| KHDRBS3 | NDT | 11 | transcription |
| KHDRBS3 | NDT | 11 | regulation of transcription DNA-dependent |
| KHDRBS3 | NDT | 11 | spermatogenesis |
| KCTD17 | NDT | 9 | potassium ion transport |
| KCTD13 | NDT | 8 | DNA replication |
| KCTD13 | NDT | 8 | potassium ion transport |
| JUP | NDT | 8 | cell-cell adhesion |
| JUNB | NDT | 10 | regulation of transcription from RNA polymerase II promoter |
| JUNB | NDT | 10 | cellular process |
| JTV1 | NDT | 9 | translation |
| JAK3 | NDT | 9 | protein amino acid phosphorylation |
| ITGB5 | NDT | 7 | cell adhesion |
| ITGB5 | NDT | 7 | cell-matrix adhesion |
| ITGB5 | NDT | 7 | integrin-mediated signaling pathway |
| ITGB2 | NDT | 10 | cell adhesion |
| ITGB2 | NDT | 10 | cell-matrix adhesion |
| ITGB2 | NDT | 10 | integrin-mediated signaling pathway |
| ITGB2 | NDT | 10 | apoptosis |
| ITGB2 | NDT | 10 | inflammatory response |
| ITGB2 | NDT | 10 | leukocyte adhesion |
| ITGB2 | NDT | 10 | cell-cell signaling |
| ITGB2 | NDT | 10 | regulation of cell shape |
| ITGB2 | NDT | 10 | neutrophil chemotaxis |
| ITGB2 | NDT | 10 | regulation of peptidyl-tyrosine phosphorylation |
| ITGB1 | NDT | 16 | integrin-mediated signaling pathway |
| ITGB1 | NDT | 16 | cell adhesion |
| ITGB1 | NDT | 16 | cell-matrix adhesion |
| ITGB1 | NDT | 16 | cellular defense response |
| ITGB1 | NDT | 16 | homophilic cell adhesion |
| ITGB1 | NDT | 16 | leukocyte adhesion |
| ITGB1 | NDT | 16 | cell migration |
| ITGB1 | NDT | 16 | B cell differentiation |
| ITGB1 | NDT | 16 | interspecies interaction between organisms |
| ILK | NDT | 9 | ureteric bud branching |
| ILK | NDT | 9 | protein amino acid phosphorylation |
| ILK | NDT | 9 | cell-matrix adhesion |
| ILK | NDT | 9 | integrin-mediated signaling pathway |
| ILK | NDT | 9 | positive regulation of cell proliferation |
| ILK | NDT | 9 | establishment and |
| ILF3 | NDT | 7 | M phase |
| ILF3 | NDT | 7 | transcription |
| ILF3 | NDT | 7 | negative regulation of transcription DNA-dependent |
| ILF3 | NDT | 7 | positive regulation of transcription DNA-dependent |
| IL8RB | NDT | 8 | apoptosis |
| IL8RB | NDT | 8 | chemotaxis |
| IL8RB | NDT | 8 | inflammatory response |
| IL8RB | NDT | 8 | cellular defense response |
| IL8RB | NDT | 8 | G-protein signaling coupled to IP3 second messenger (phospholipase C activating) |
| IL8RB | NDT | 8 | positive regulation of cell proliferation |
| IL8RB | NDT | 8 | neutrophil chemotaxis |
| IL8RB | NDT | 8 | neutrophil activation |
| IL8RA | NDT | 8 | chemotaxis |
| IL8RA | NDT | 8 | inflammatory response |
| IL8RA | NDT | 8 | G-protein coupled receptor protein signaling pathway |
| IL2RG | NDT | 8 | immune response |
| IL2RG | NDT | 8 | signal transduction |
| IL2RG | NDT | 8 | interspecies interaction between organisms |
| IL2RB | DT | 11 | protein complex assembly |
| IL2RB | DT | 11 | signal transduction |
| IL2RB | DT | 11 | cytokine and chemokine mediated signaling pathway |
| IL2RB | DT | 11 | interspecies interaction between organisms |
| IL2RB | DT | 11 | positive regulation of survival gene product expression |
| IL16 | NDT | 9 | immune response |
| IL16 | NDT | 9 | leukocyte chemotaxis |
| IL16 | NDT | 9 | induction of positive chemotaxis |
| IKBKG | NDT | 14 | transcription |
| IKBKG | NDT | 14 | regulation of transcription DNA-dependent |
| IKBKG | NDT | 14 | induction of apoptosis |
| IKBKG | NDT | 14 | immune response |
| IKBKG | NDT | 14 | I-kappaB kinase |
| IKBKG | NDT | 14 | interspecies interaction between organisms |
| IKBKG | NDT | 14 | T cell receptor signaling pathway |
| HSPD1 | NDT | 8 | protein folding |
| HSPD1 | NDT | 8 | response to unfolded protein |
| HSPD1 | NDT | 8 | protein import into mitochondrial matrix |
| HSPD1 | NDT | 8 | regulation of apoptosis |
| HSPD1 | NDT | 8 | interspecies interaction between organisms |
| HSPCA | DT | 24 | mitochondrial transport |
| HSPCA | DT | 24 | response to unfolded protein |
| HSPCA | DT | 24 | signal transduction |
| HSPCA | DT | 24 | protein refolding |
| HSPCA | DT | 24 | positive regulation of nitric oxide biosynthetic process |
| HSPA8 | NDT | 19 | protein folding |
| HSPA8 | NDT | 19 | response to unfolded protein |
| HSPA1A | DT | 17 | response to stress |
| HSPA1A | DT | 17 | mRNA catabolic process |
| HSPA1A | DT | 17 | anti-apoptosis |
| HSPA1A | DT | 17 | response to unfolded protein |
| HSF1 | NDT | 11 | transcription |
| HSF1 | NDT | 11 | regulation of transcription DNA-dependent |
| HSF1 | NDT | 11 | response to stress |
| HOXA1 | NDT | 10 | regulation of transcription DNA-dependent |
| HOXA1 | NDT | 10 | multicellular organismal development |
| HOOK2 | NDT | 16 | microtubule cytoskeleton organization and biogenesis |
| HOOK2 | NDT | 16 | endocytosis |
| HNRPK | NDT | 22 | nuclear mRNA splicing via spliceosome |
| HNRPK | NDT | 22 | signal transduction |
| HNRPK | NDT | 22 | RNA splicing |
| HNRPK | NDT | 22 | interspecies interaction between organisms |
| HNRPC | NDT | 14 | nuclear mRNA splicing via spliceosome |
| HNRPC | NDT | 14 | RNA splicing |
| HMGB2 | NDT | 6 | DNA replication |
| HMGB2 | NDT | 6 | DNA unwinding during replication |
| HMGB2 | NDT | 6 | DNA repair |
| HMGB2 | NDT | 6 | base-excision repair DNA ligation |
| HMGB2 | NDT | 6 | establishment and |
| HMGB2 | NDT | 6 | nucleosome assembly |
| HMGB2 | NDT | 6 | regulation of transcription from RNA polymerase II promoter |
| HMGB2 | NDT | 6 | phosphoinositide-mediated signaling |
| HMGB1 | NDT | 6 | regulation of transcription DNA-dependent |
| HMGB1 | NDT | 6 | DNA unwinding during replication |
| HMGB1 | NDT | 6 | DNA repair |
| HMGB1 | NDT | 6 | base-excision repair DNA ligation |
| HMGB1 | NDT | 6 | DNA recombination |
| HMGB1 | NDT | 6 | establishment and |
| HMGB1 | NDT | 6 | regulation of transcription from RNA polymerase II promoter |
| HMGB1 | NDT | 6 | anti-apoptosis |
| HMGB1 | NDT | 6 | signal transduction |
| HMGB1 | NDT | 6 | negative regulation of transcriptional preinitiation complex assembly |
| HMG20A | NDT | 7 | transcription |
| HMG20A | NDT | 7 | regulation of transcription DNA-dependent |
| HMG20A | NDT | 7 | chromatin modification |
| HLA-G | NDT | 6 | immune response |
| HLA-G | NDT | 6 | antigen processing and presentation |
| HLA-G | NDT | 6 | antigen processing and presentation of peptide antigen via MHC class I |
| HLA-G | NDT | 6 | cellular defense response |
| HLA-B | NDT | 6 | antigen processing and presentation of peptide antigen via MHC class I |
| HLA-B | NDT | 6 | immune response |
| HLA-B | NDT | 6 | antigen processing and presentation |
| HLA-B | NDT | 6 | interspecies interaction between organisms |
| HLA-B | NDT | 6 | defense response |
| HLA-B | NDT | 6 | biological process |
| HIF1AN | DT | 7 | transcription |
| HIF1AN | DT | 7 | regulation of transcription DNA-dependent |
| HIF1AN | DT | 7 | oxidation reduction |
| HIF1A | NDT | 7 | regulation of transcription DNA-dependent |
| HIF1A | NDT | 7 | signal transduction |
| HIF1A | NDT | 7 | response to hypoxia |
| HIF1A | NDT | 7 | epithelial to mesenchymal transition |
| HIF1A | NDT | 7 | positive regulation of endothelial cell proliferation |
| HIF1A | NDT | 7 | connective tissue replacement during inflammatory response |
| HIF1A | NDT | 7 | positive regulation vascular endothelial growth factor production |
| HIF1A | NDT | 7 | positive regulation of cell migration |
| HIF1A | NDT | 7 | positive regulation of vascular endothelial growth factor receptor signaling pathway |
| HIF1A | NDT | 7 | oxygen homeostasis |
| HIF1A | NDT | 7 | positive regulation of chemokine production |
| HIF1A | NDT | 7 | regulation of transforming growth factor-beta2 production |
| HIF1A | NDT | 7 | collagen metabolic process |
| HIF1A | NDT | 7 | mRNA transcription from RNA polymerase II promoter |
| HIF1A | NDT | 7 | positive regulation of gene-specific transcription |
| HIF1A | NDT | 7 | regulation of transcription from RNA polymerase II promoter in response to oxidative stress |
| HIF1A | NDT | 7 | positive regulation of erythrocyte differentiation |
| HIF1A | NDT | 7 | positive regulation of angiogenesis |
| HIF1A | NDT | 7 | positive regulation of glycolysis |
| HIF1A | NDT | 7 | positive regulation of transcription |
| HIF1A | NDT | 7 | positive regulation of transcription from RNA polymerase II promoter |
| HIF1A | NDT | 7 | positive regulation of hormone biosynthetic process |
| HIF1A | NDT | 7 | positive regulation of nitric-oxide synthase activity |
| HIF1A | NDT | 7 | elastin metabolic process |
| HGS | NDT | 23 | intracellular protein transport |
| HGS | NDT | 23 | signal transduction |
| HGS | NDT | 23 | negative regulation of cell proliferation |
| HGS | NDT | 23 | endosome transport |
| HGS | NDT | 23 | regulation of protein catabolic process |
| HGS | NDT | 23 | negative regulation of JAK-STAT cascade |
| HDAC3 | NDT | 18 | transcription |
| HDAC3 | NDT | 18 | regulation of transcription DNA-dependent |
| HDAC3 | NDT | 18 | anti-apoptosis |
| HDAC3 | NDT | 18 | chromatin modification |
| HDAC3 | NDT | 18 | histone deacetylation |
| HCK | DT | 8 | protein amino acid phosphorylation |
| HCK | DT | 8 | mesoderm development |
| HCK | DT | 8 | interspecies interaction between organisms |
| GTF2F2 | NDT | 6 | transcription initiation from RNA polymerase II promoter |
| GTF2F2 | NDT | 6 | nuclear mRNA splicing via spliceosome |
| GTF2F2 | NDT | 6 | transcription |
| GTF2F2 | NDT | 6 | regulation of transcription DNA-dependent |
| GTF2F2 | NDT | 6 | RNA elongation from RNA polymerase II promoter |
| GSK3B | DT | 8 | glycogen metabolic process |
| GSK3B | DT | 8 | ER overload response |
| GSK3B | DT | 8 | intracellular signaling cascade |
| GSK3B | DT | 8 | peptidyl-serine phosphorylation |
| GSK3B | DT | 8 | negative regulation of apoptosis |
| GSK3B | DT | 8 | positive regulation of protein export from nucleus |
| GSK3B | DT | 8 | Wnt receptor signaling pathway through beta-catenin |
| GRN | NDT | 6 | signal transduction |
| GRAP2 | NDT | 13 | Ras protein signal transduction |
| GRAP2 | NDT | 13 | cell-cell signaling |
| GORASP2 | NDT | 13 | Golgi organization and biogenesis |
| GNAZ | NDT | 6 | signal transduction |
| GNAZ | NDT | 6 | G-protein coupled receptor protein signaling pathway |
| GNAO1 | NDT | 6 | muscle contraction |
| GNAO1 | NDT | 6 | signal transduction |
| GNAO1 | NDT | 6 | G-protein coupled receptor protein signaling pathway |
| GNAI2 | NDT | 15 | signal transduction |
| GNAI2 | NDT | 15 | G-protein coupled receptor protein signaling pathway |
| GNAI2 | NDT | 15 | negative regulation of adenylate cyclase activity |
| GNAI2 | NDT | 15 | response to nutrient |
| GNAI1 | NDT | 11 | signal transduction |
| GNAI1 | NDT | 11 | G-protein coupled receptor protein signaling pathway |
| GNA13 | NDT | 8 | patterning of blood vessels |
| GNA13 | NDT | 8 | in utero embryonic development |
| GNA13 | NDT | 8 | cell motility |
| GNA13 | NDT | 8 | signal transduction |
| GNA13 | NDT | 8 | G-protein coupled receptor protein signaling pathway |
| GNA13 | NDT | 8 | protein kinase cascade |
| GNA13 | NDT | 8 | Rho protein signal transduction |
| GNA13 | NDT | 8 | regulation of cell shape |
| GNA13 | NDT | 8 | cell differentiation |
| GNA13 | NDT | 8 | platelet activation |
| GNA13 | NDT | 8 | regulation of cell migration |
| GNA12 | NDT | 6 | signal transduction |
| GNA12 | NDT | 6 | G-protein coupled receptor protein signaling pathway |
| GNA12 | NDT | 6 | blood coagulation |
| GJA1 | NDT | 6 | in utero embryonic development |
| GJA1 | NDT | 6 | neuron migration |
| GJA1 | NDT | 6 | heart looping |
| GJA1 | NDT | 6 | epithelial cell maturation |
| GJA1 | NDT | 6 | apoptosis |
| GJA1 | NDT | 6 | muscle contraction |
| GJA1 | NDT | 6 | cell-cell signaling |
| GJA1 | NDT | 6 | adult heart development |
| GJA1 | NDT | 6 | sensory perception of sound |
| GJA1 | NDT | 6 | regulation of heart contraction |
| GJA1 | NDT | 6 | negative regulation of cell proliferation |
| GJA1 | NDT | 6 | response to pH |
| GJA1 | NDT | 6 | vascular transport |
| GJA1 | NDT | 6 | ATP transport |
| GJA1 | NDT | 6 | gap junction assembly |
| GJA1 | NDT | 6 | embryonic heart tube development |
| GJA1 | NDT | 6 | positive regulation of I-kappaB kinase |
| GJA1 | NDT | 6 | skeletal muscle regeneration |
| GJA1 | NDT | 6 | positive regulation of protein catabolic process |
| GJA1 | NDT | 6 | positive regulation of striated muscle development |
| GJA1 | NDT | 6 | blood vessel morphogenesis |
| GJA1 | NDT | 6 | neurite morphogenesis |
| GJA1 | NDT | 6 | protein oligomerization |
| GJA1 | NDT | 6 | regulation of calcium ion transport |
| GDI2 | NDT | 6 | protein transport |
| GDI2 | NDT | 6 | regulation of GTPase activity |
| GDI2 | NDT | 6 | signal transduction |
| GAK | NDT | 7 | protein amino acid phosphorylation |
| GAK | NDT | 7 | cell cycle |
| GADD45G | NDT | 10 | activation of MAPKKK activity |
| GADD45G | NDT | 10 | DNA repair |
| GADD45G | NDT | 10 | apoptosis |
| GADD45G | NDT | 10 | response to stress |
| GADD45G | NDT | 10 | multicellular organismal development |
| GADD45G | NDT | 10 | cell differentiation |
| GADD45A | NDT | 7 | regulation of cyclin-dependent protein kinase activity |
| GADD45A | NDT | 7 | DNA repair |
| GADD45A | NDT | 7 | apoptosis |
| GADD45A | NDT | 7 | cell cycle |
| GADD45A | NDT | 7 | cell cycle arrest |
| GABARAPL2 | NDT | 6 | intra-Golgi vesicle-mediated transport |
| GABARAPL2 | NDT | 6 | autophagy |
| GABARAPL2 | NDT | 6 | protein transport |
| GABARAPL2 | NDT | 6 | positive regulation of ATPase activity |
| G22P1 | NDT | 14 | DNA ligation |
| G22P1 | NDT | 14 | DNA repair |
| G22P1 | NDT | 14 | double-strand break repair via nonhomologous end joining |
| G22P1 | NDT | 14 | provirus integration |
| G22P1 | NDT | 14 | initiation of viral infection |
| G22P1 | NDT | 14 | positive regulation of transcription DNA-dependent |
| FOS | NDT | 16 | DNA methylation |
| FOS | NDT | 16 | regulation of transcription from RNA polymerase II promoter |
| FOS | NDT | 16 | inflammatory response |
| FOS | NDT | 16 | nervous system development |
| FNBP3 | NDT | 23 | mRNA processing |
| FNBP3 | NDT | 23 | RNA splicing |
| FKBP1A | DT | 6 | protein folding |
| FKBP1A | DT | 6 | positive regulation of I-kappaB kinase |
| FHL3 | NDT | 12 | muscle development |
| FGFR1 | NDT | 6 | MAPKKK cascade |
| FGFR1 | NDT | 6 | skeletal development |
| FGFR1 | NDT | 6 | protein amino acid phosphorylation |
| FGFR1 | NDT | 6 | fibroblast growth factor receptor signaling pathway |
| FGFR1 | NDT | 6 | cell growth |
| FCGR3A | NDT | 7 | immune response |
| FCGR1A | DT | 6 | phagocytosis engulfment |
| FCGR1A | DT | 6 | signal transduction |
| FCGR1A | DT | 6 | innate immune response |
| FAS | NDT | 8 | apoptosis |
| FAS | NDT | 8 | immune response |
| FAS | NDT | 8 | signal transduction |
| FAS | NDT | 8 | protein complex assembly |
| FAS | NDT | 8 | anti-apoptosis |
| FAS | NDT | 8 | induction of apoptosis by extracellular signals |
| FAS | NDT | 8 | activation of pro-apoptotic gene products |
| FAS | NDT | 8 | regulation of apoptosis |
| FAS | NDT | 8 | fatty acid biosynthetic process |
| FAS | NDT | 8 | oxidation reduction |
| FANCG | NDT | 8 | cell cycle checkpoint |
| FANCG | NDT | 8 | DNA repair |
| FANCG | NDT | 8 | caspase activation |
| FADD | NDT | 12 | signal transduction |
| FADD | NDT | 12 | induction of apoptosis via death domain receptors |
| FADD | NDT | 12 | activation of pro-apoptotic gene products |
| FADD | NDT | 12 | regulation of apoptosis |
| FADD | NDT | 12 | positive regulation of I-kappaB kinase |
| FADD | NDT | 12 | interspecies interaction between organisms |
| F2R | NDT | 6 | connective tissue replacement during inflammatory response |
| F2R | NDT | 6 | caspase activation |
| F2R | NDT | 6 | signal transduction |
| F2R | NDT | 6 | G-protein coupled receptor protein signaling pathway |
| F2R | NDT | 6 | tyrosine phosphorylation of STAT protein |
| F2R | NDT | 6 | STAT protein nuclear translocation |
| F2R | NDT | 6 | negative regulation of cell proliferation |
| F2R | NDT | 6 | response to wounding |
| F2R | NDT | 6 | anatomical structure morphogenesis |
| F2R | NDT | 6 | platelet activation |
| F2R | NDT | 6 | positive regulation of blood coagulation |
| F2R | NDT | 6 | positive regulation of cell migration |
| F2R | NDT | 6 | positive regulation of collagen biosynthetic process |
| F2R | NDT | 6 | positive regulation of I-kappaB kinase |
| F2R | NDT | 6 | positive regulation of MAPKKK cascade |
| F2R | NDT | 6 | positive regulation of transcription DNA-dependent |
| F2R | NDT | 6 | positive regulation of JAK-STAT cascade |
| F2R | NDT | 6 | release of sequestered calcium ion into cytosol |
| EXOSC5 | NDT | 6 | rRNA processing |
| EXOSC5 | NDT | 6 | RNA processing |
| EXOSC4 | NDT | 11 | rRNA processing |
| EXOSC4 | NDT | 11 | RNA processing |
| EXOC8 | NDT | 10 | exocytosis |
| EXOC8 | NDT | 10 | protein transport |
| EXOC7 | NDT | 8 | exocytosis |
| EXOC7 | NDT | 8 | protein transport |
| ETS1 | NDT | 7 | transcription |
| ETS1 | NDT | 7 | regulation of transcription DNA-dependent |
| ETS1 | NDT | 7 | transcription from RNA polymerase II promoter |
| ETS1 | NDT | 7 | induction of apoptosis |
| ETS1 | NDT | 7 | immune response |
| ETS1 | NDT | 7 | negative regulation of cell proliferation |
| ETS1 | NDT | 7 | positive regulation of erythrocyte differentiation |
| ETS1 | NDT | 7 | negative regulation of cell cycle |
| ETS1 | NDT | 7 | positive regulation of transcription |
| ETS1 | NDT | 7 | response to antibiotic |
| ESR2 | DT | 8 | transcription |
| ESR2 | DT | 8 | regulation of transcription DNA-dependent |
| ESR2 | DT | 8 | signal transduction |
| ESR2 | DT | 8 | cell-cell signaling |
| ESR2 | DT | 8 | negative regulation of cell growth |
| ESR2 | DT | 8 | estrogen receptor signaling pathway |
| ERBB3 | DT | 8 | protein amino acid phosphorylation |
| ERBB3 | DT | 8 | transmembrane receptor protein tyrosine kinase signaling pathway |
| ERBB3 | DT | 8 | negative regulation of cell adhesion |
| ERBB3 | DT | 8 | heart development |
| ERBB3 | DT | 8 | negative regulation of signal transduction |
| ERBB3 | DT | 8 | Schwann cell differentiation |
| ERBB3 | DT | 8 | positive regulation of phosphoinositide 3-kinase cascade |
| ERBB3 | DT | 8 | cranial nerve development |
| ERBB3 | DT | 8 | wound healing |
| ERBB3 | DT | 8 | regulation of cell proliferation |
| ERBB3 | DT | 8 | negative regulation of neuron apoptosis |
| ERBB3 | DT | 8 | negative regulation of secretion |
| EPS8 | NDT | 9 | signal transduction |
| EPS8 | NDT | 9 | epidermal growth factor receptor signaling pathway |
| EPS8 | NDT | 9 | cell proliferation |
| EMD | NDT | 7 | muscle contraction |
| EMD | NDT | 7 | muscle development |
| EIF4E | DT | 7 | translational initiation |
| EIF4E | DT | 7 | regulation of translation |
| EIF4E | DT | 7 | interspecies interaction between organisms |
| EIF3S6 | NDT | 6 | translation |
| EIF3S6 | NDT | 6 | regulation of translational initiation |
| EFEMP2 | NDT | 28 | blood coagulation |
| EFCBP2 | NDT | 18 | antibiotic biosynthetic process |
| EEF1G | NDT | 7 | translational elongation |
| EEF1A1 | NDT | 8 | translational elongation |
| EDF1 | NDT | 8 | regulation of transcription DNA-dependent |
| EDF1 | NDT | 8 | multicellular organismal development |
| EDF1 | NDT | 8 | regulation of lipid metabolic process |
| EDF1 | NDT | 8 | positive regulation of DNA binding |
| EDF1 | NDT | 8 | endothelial cell differentiation |
| DVL3 | NDT | 15 | intracellular signaling cascade |
| DVL3 | NDT | 15 | multicellular organismal development |
| DVL3 | NDT | 15 | nervous system development |
| DVL3 | NDT | 15 | heart development |
| DVL3 | NDT | 15 | Wnt receptor signaling pathway |
| DTX2 | NDT | 11 | Notch signaling pathway |
| DRAP1 | NDT | 6 | negative regulation of transcription from RNA polymerase II promoter |
| DRAP1 | NDT | 6 | transcription |
| DRAP1 | NDT | 6 | regulation of transcription DNA-dependent |
| DNAJB1 | NDT | 6 | protein folding |
| DNAJB1 | NDT | 6 | response to unfolded protein |
| DNAJB1 | NDT | 6 | chaperone cofactor-dependent protein folding |
| DNAJA3 | NDT | 7 | protein folding |
| DNAJA3 | NDT | 7 | regulation of apoptosis |
| DMRTB1 | NDT | 9 | transcription |
| DMRTB1 | NDT | 9 | regulation of transcription DNA-dependent |
| DMRTB1 | NDT | 9 | sex differentiation |
| DLC2 | NDT | 7 | microtubule-based process |
| DLC2 | NDT | 7 | signal transduction |
| DLC2 | NDT | 7 | negative regulation of cell cycle |
| DLAT | NDT | 7 | acetyl-CoA biosynthetic process |
| DLAT | NDT | 7 | pyruvate metabolic process |
| DLAT | NDT | 7 | glycolysis |
| DLAT | NDT | 7 | metabolic process |
| DDIT3 | NDT | 17 | response to amphetamine |
| DDIT3 | NDT | 17 | regulation of transcription DNA-dependent |
| DDIT3 | NDT | 17 | response to DNA damage stimulus |
| DDIT3 | NDT | 17 | response to oxidative stress |
| DDIT3 | NDT | 17 | ER overload response |
| DDIT3 | NDT | 17 | cell cycle |
| DDIT3 | NDT | 17 | cell cycle arrest |
| DDIT3 | NDT | 17 | aging |
| DDIT3 | NDT | 17 | response to nutrient |
| DDIT3 | NDT | 17 | regulation of cell redox homeostasis |
| DDIT3 | NDT | 17 | endoplasmic reticulum unfolded protein response |
| DDIT3 | NDT | 17 | negative regulation of CREB transcription factor activity |
| DDIT3 | NDT | 17 | response to hydrogen peroxide |
| DDIT3 | NDT | 17 | mRNA transcription from RNA polymerase II promoter |
| DDIT3 | NDT | 17 | positive regulation of apoptosis |
| DDIT3 | NDT | 17 | positive regulation of transcription |
| DDIT3 | NDT | 17 | embryonic organ development |
| DCTN1 | NDT | 8 | mitosis |
| DCTN1 | NDT | 8 | nervous system development |
| DCN | NDT | 6 | organ morphogenesis |
| DAB2 | NDT | 8 | cell proliferation |
| CYCS | NDT | 9 | DNA fragmentation during apoptosis |
| CYCS | NDT | 9 | transport |
| CYCS | NDT | 9 | apoptosis |
| CYCS | NDT | 9 | caspase activation via cytochrome c |
| CYCS | NDT | 9 | cellular respiration |
| CUTC | NDT | 6 | copper ion homeostasis |
| CUL1 | NDT | 7 | G1 |
| CUL1 | NDT | 7 | ubiquitin-dependent protein catabolic process |
| CUL1 | NDT | 7 | ubiquitin cycle |
| CUL1 | NDT | 7 | apoptosis |
| CUL1 | NDT | 7 | cell cycle arrest |
| CUL1 | NDT | 7 | negative regulation of cell proliferation |
| CUL1 | NDT | 7 | induction of apoptosis by intracellular signals |
| CUL1 | NDT | 7 | organ morphogenesis |
| CUL1 | NDT | 7 | positive regulation of ubiquitin-protein ligase activity during mitotic cell cycle |
| CTBP2 | NDT | 12 | metabolic process |
| CTBP2 | NDT | 12 | negative regulation of cell proliferation |
| CTBP2 | NDT | 12 | viral genome replication |
| CTBP2 | NDT | 12 | oxidation reduction |
| CTBP1 | DT | 8 | metabolic process |
| CTBP1 | DT | 8 | protein amino acid phosphorylation |
| CTBP1 | DT | 8 | negative regulation of cell proliferation |
| CTBP1 | DT | 8 | viral genome replication |
| CTBP1 | DT | 8 | oxidation reduction |
| CSNK2A2 | NDT | 7 | protein amino acid phosphorylation |
| CSNK2A2 | NDT | 7 | signal transduction |
| CSNK2A2 | NDT | 7 | spermatid development |
| CSNK2A2 | NDT | 7 | Wnt receptor signaling pathway |
| CSNK1E | NDT | 6 | protein amino acid phosphorylation |
| CSNK1E | NDT | 6 | DNA repair |
| CSNK1E | NDT | 6 | signal transduction |
| CSNK1D | NDT | 6 | DNA repair |
| CSNK1D | NDT | 6 | protein amino acid phosphorylation |
| CSNK1D | NDT | 6 | signal transduction |
| CSNK1D | NDT | 6 | Wnt receptor signaling pathway |
| CSEN | NDT | 6 | transcription |
| CSEN | NDT | 6 | regulation of transcription from RNA polymerase II promoter |
| CSEN | NDT | 6 | ion transport |
| CSEN | NDT | 6 | potassium ion transport |
| CSEN | NDT | 6 | apoptosis |
| CSEN | NDT | 6 | signal transduction |
| CRY1 | NDT | 8 | DNA repair |
| CRY1 | NDT | 8 | transcription |
| CRY1 | NDT | 8 | regulation of transcription DNA-dependent |
| CRY1 | NDT | 8 | circadian rhythm |
| CRY1 | NDT | 8 | protein-chromophore linkage |
| CRY1 | NDT | 8 | response to stimulus |
| CRK | NDT | 10 | regulation of transcription from RNA polymerase II promoter |
| CRK | NDT | 10 | intracellular signaling cascade |
| CRK | NDT | 10 | actin cytoskeleton organization and biogenesis |
| CREM | NDT | 6 | regulation of transcription DNA-dependent |
| CREM | NDT | 6 | transcription |
| CREM | NDT | 6 | signal transduction |
| CREM | NDT | 6 | multicellular organismal development |
| CREM | NDT | 6 | spermatogenesis |
| CREM | NDT | 6 | cell differentiation |
| CREB3 | NDT | 9 | transcription |
| CREB3 | NDT | 9 | regulation of transcription DNA-dependent |
| CREB3 | NDT | 9 | chemotaxis |
| CREB3 | NDT | 9 | reactivation of latent virus |
| CREB3 | NDT | 9 | positive regulation of cell migration |
| CREB3 | NDT | 9 | regulation of cell proliferation |
| CREB3 | NDT | 9 | interspecies interaction between organisms |
| CREB3 | NDT | 9 | negative regulation of cell cycle |
| CREB3 | NDT | 9 | induction of positive chemotaxis |
| CREB3 | NDT | 9 | positive regulation of calcium ion transport |
| CREB1 | NDT | 8 | regulation of transcription DNA-dependent |
| CREB1 | NDT | 8 | protein amino acid phosphorylation |
| CREB1 | NDT | 8 | signal transduction |
| CREB1 | NDT | 8 | interspecies interaction between organisms |
| CPSF6 | NDT | 10 | mRNA processing |
| CPSF5 | NDT | 6 | nuclear mRNA splicing via spliceosome |
| COPB | NDT | 9 | intracellular protein transport |
| COPB | NDT | 9 | retrograde vesicle-mediated transport Golgi to ER |
| COPB | NDT | 9 | intra-Golgi vesicle-mediated transport |
| COPB | NDT | 9 | membrane organization and biogenesis |
| COPB | NDT | 9 | vesicle-mediated transport |
| COPB | NDT | 9 | interspecies interaction between organisms |
| COPB | NDT | 9 | COPI coating of Golgi vesicle |
| COL4A6 | NDT | 6 | phosphate transport |
| COL4A6 | NDT | 6 | cell adhesion |
| COL4A6 | NDT | 6 | extracellular matrix organization and biogenesis |
| COL2A1 | NDT | 11 | skeletal development |
| COL2A1 | NDT | 11 | phosphate transport |
| COL2A1 | NDT | 11 | sensory perception of sound |
| COL1A1 | DT | 14 | ossification |
| COL1A1 | DT | 14 | phosphate transport |
| COL1A1 | DT | 14 | response to nutrient |
| COL1A1 | DT | 14 | sensory perception of sound |
| COL1A1 | DT | 14 | epidermis development |
| COL1A1 | DT | 14 | response to mechanical stimulus |
| COL1A1 | DT | 14 | response to inorganic substance |
| COL1A1 | DT | 14 | response to corticosteroid stimulus |
| COL1A1 | DT | 14 | response to hydrogen peroxide |
| COL1A1 | DT | 14 | response to peptide hormone stimulus |
| COL1A1 | DT | 14 | response to cAMP |
| CHIC2 | NDT | 8 | biological process |
| CHEK2 | NDT | 7 | DNA damage checkpoint |
| CHEK2 | NDT | 7 | protein amino acid phosphorylation |
| CHEK2 | NDT | 7 | cell cycle |
| CHEK2 | NDT | 7 | DNA damage response signal transduction resulting in induction of apoptosis |
| CGI-116 | NDT | 10 | biological process |
| CFLAR | NDT | 8 | proteolysis |
| CFLAR | NDT | 8 | anti-apoptosis |
| CFLAR | NDT | 8 | induction of apoptosis by extracellular signals |
| CFLAR | NDT | 8 | regulation of apoptosis |
| CFLAR | NDT | 8 | positive regulation of I-kappaB kinase |
| CFLAR | NDT | 8 | interspecies interaction between organisms |
| CFL1 | NDT | 10 | anti-apoptosis |
| CFL1 | NDT | 10 | Rho protein signal transduction |
| CFL1 | NDT | 10 | actin cytoskeleton organization and biogenesis |
| CEBPG | NDT | 14 | liver development |
| CEBPG | NDT | 14 | transcription |
| CEBPG | NDT | 14 | regulation of transcription from RNA polymerase II promoter |
| CEBPG | NDT | 14 | immune response |
| CEBPG | NDT | 14 | B cell differentiation |
| CEBPG | NDT | 14 | natural killer cell mediated cytotoxicity |
| CEBPG | NDT | 14 | enucleate erythrocyte differentiation |
| CEBPG | NDT | 14 | negative regulation of transcription factor activity |
| CEBPG | NDT | 14 | positive regulation of interferon-gamma biosynthetic process |
| CEBPG | NDT | 14 | positive regulation of DNA repair |
| CEBPG | NDT | 14 | positive regulation of transcription factor activity |
| CDKN1B | NDT | 11 | cell cycle arrest |
| CDKN1B | NDT | 11 | G1 |
| CDKN1B | NDT | 11 | potassium ion transport |
| CDKN1B | NDT | 11 | induction of apoptosis |
| CDKN1B | NDT | 11 | cell cycle |
| CDKN1B | NDT | 11 | sensory perception of sound |
| CDKN1B | NDT | 11 | positive regulation of cell proliferation |
| CDKN1B | NDT | 11 | negative regulation of cell growth |
| CDKN1B | NDT | 11 | positive regulation of microtubule polymerization |
| CDKN1B | NDT | 11 | negative regulation of phosphorylation |
| CDKN1B | NDT | 11 | negative regulation of cyclin-dependent protein kinase activity |
| CDKN1B | NDT | 11 | autophagic cell death |
| CDKN1B | NDT | 11 | inner ear development |
| CDKN1B | NDT | 11 | negative regulation of epithelial cell proliferation |
| CDKN1B | NDT | 11 | negative regulation of cell motility |
| CDKN1A | NDT | 23 | G1 |
| CDKN1A | NDT | 23 | G2 |
| CDKN1A | NDT | 23 | response to DNA damage stimulus |
| CDKN1A | NDT | 23 | cell cycle |
| CDKN1A | NDT | 23 | cell cycle arrest |
| CDKN1A | NDT | 23 | negative regulation of cell proliferation |
| CDKN1A | NDT | 23 | induction of apoptosis by intracellular signals |
| CDKN1A | NDT | 23 | response to UV |
| CDKN1A | NDT | 23 | negative regulation of cell growth |
| CDKN1A | NDT | 23 | positive regulation of B cell proliferation |
| CDKN1A | NDT | 23 | cellular response to extracellular stimulus |
| CDKN1A | NDT | 23 | negative regulation of phosphorylation |
| CDKN1A | NDT | 23 | negative regulation of apoptosis |
| CDKN1A | NDT | 23 | positive regulation of non-apoptotic programmed cell death |
| CDKN1A | NDT | 23 | negative regulation of cyclin-dependent protein kinase activity |
| CDKN1A | NDT | 23 | positive regulation of fibroblast proliferation |
| CDK6 | NDT | 9 | G1 phase of mitotic cell cycle |
| CDK6 | NDT | 9 | positive regulation of cell-matrix adhesion |
| CDK6 | NDT | 9 | protein amino acid phosphorylation |
| CDK6 | NDT | 9 | cell cycle |
| CDK6 | NDT | 9 | regulation of gene expression |
| CDK6 | NDT | 9 | gliogenesis |
| CDK6 | NDT | 9 | cell dedifferentiation |
| CDK6 | NDT | 9 | regulation of erythrocyte differentiation |
| CDK6 | NDT | 9 | negative regulation of osteoblast differentiation |
| CDK6 | NDT | 9 | positive regulation of fibroblast proliferation |
| CDK6 | NDT | 9 | negative regulation of epithelial cell proliferation |
| CDK6 | NDT | 9 | cell division |
| CDK5 | NDT | 6 | protein amino acid phosphorylation |
| CDK5 | NDT | 6 | cell cycle |
| CDK5 | NDT | 6 | cell-matrix adhesion |
| CDK5 | NDT | 6 | axonogenesis |
| CDK5 | NDT | 6 | motor axon guidance |
| CDK5 | NDT | 6 | cell proliferation |
| CDK5 | NDT | 6 | embryonic development |
| CDK5 | NDT | 6 | Schwann cell development |
| CDK5 | NDT | 6 | neuron differentiation |
| CDK5 | NDT | 6 | regulation of cell migration |
| CDK5 | NDT | 6 | neurite development |
| CDK5 | NDT | 6 | receptor clustering |
| CDK5 | NDT | 6 | positive regulation of neuron apoptosis |
| CDK5 | NDT | 6 | positive regulation of protein kinase activity |
| CDK5 | NDT | 6 | cell division |
| CDK5 | NDT | 6 | regulation of postsynaptic membrane potential |
| CDK4 | NDT | 16 | G1 |
| CDK4 | NDT | 16 | protein amino acid phosphorylation |
| CDK4 | NDT | 16 | cell cycle |
| CDK4 | NDT | 16 | regulation of gene expression |
| CDK4 | NDT | 16 | positive regulation of fibroblast proliferation |
| CDK4 | NDT | 16 | cell division |
| CDK2 | DT | 18 | G2 |
| CDK2 | DT | 18 | regulation of DNA replication |
| CDK2 | DT | 18 | protein amino acid phosphorylation |
| CDK2 | DT | 18 | cell cycle |
| CDK2 | DT | 18 | mitosis |
| CDK2 | DT | 18 | traversing start control point of mitotic cell cycle |
| CDK2 | DT | 18 | positive regulation of cell proliferation |
| CDK2 | DT | 18 | cell division |
| CDC5L | NDT | 7 | regulation of transcription DNA-dependent |
| CDC5L | NDT | 7 | mRNA processing |
| CDC5L | NDT | 7 | cell cycle |
| CDC5L | NDT | 7 | RNA splicing |
| CDC42EP1 | NDT | 6 | Rho protein signal transduction |
| CDC42EP1 | NDT | 6 | regulation of cell shape |
| CDC42EP1 | NDT | 6 | positive regulation of pseudopodium formation |
| CDC42 | NDT | 21 | small GTPase mediated signal transduction |
| CDC42 | NDT | 21 | cell division |
| CDC42 | NDT | 21 | nuclear migration |
| CDC42 | NDT | 21 | establishment and |
| CDC42 | NDT | 21 | actin cytoskeleton organization and biogenesis |
| CDC42 | NDT | 21 | macrophage differentiation |
| CDC42 | NDT | 21 | positive regulation of pseudopodium formation |
| CDC42 | NDT | 21 | negative regulation of protein complex assembly |
| CDC42 | NDT | 21 | positive regulation of phosphoinositide 3-kinase activity |
| CDC42 | NDT | 21 | filopodium formation |
| CDC27 | NDT | 7 | mitotic metaphase |
| CDC27 | NDT | 7 | cell proliferation |
| CDC27 | NDT | 7 | anaphase-promoting complex-dependent proteasomal ubiquitin-dependent protein catabolic process |
| CDC27 | NDT | 7 | negative regulation of ubiquitin-protein ligase activity during mitotic cell cycle |
| CDC27 | NDT | 7 | positive regulation of ubiquitin-protein ligase activity during mitotic cell cycle |
| CDC25C | NDT | 8 | M phase of mitotic cell cycle |
| CDC25C | NDT | 8 | protein amino acid dephosphorylation |
| CDC25C | NDT | 8 | cell division |
| CDC25C | NDT | 8 | regulation of cyclin-dependent protein kinase activity |
| CDC25C | NDT | 8 | DNA replication |
| CDC25C | NDT | 8 | cell cycle |
| CDC25C | NDT | 8 | regulation of mitosis |
| CDC25C | NDT | 8 | traversing start control point of mitotic cell cycle |
| CDC25C | NDT | 8 | cell proliferation |
| CDC25C | NDT | 8 | interspecies interaction between organisms |
| CDC25A | NDT | 6 | regulation of cyclin-dependent protein kinase activity |
| CDC25A | NDT | 6 | DNA replication |
| CDC25A | NDT | 6 | protein amino acid dephosphorylation |
| CDC25A | NDT | 6 | cell cycle |
| CDC25A | NDT | 6 | mitosis |
| CDC25A | NDT | 6 | cell proliferation |
| CDC25A | NDT | 6 | cell division |
| CDC2 | NDT | 24 | protein amino acid phosphorylation |
| CDC2 | NDT | 24 | anti-apoptosis |
| CDC2 | NDT | 24 | cell cycle |
| CDC2 | NDT | 24 | mitosis |
| CDC2 | NDT | 24 | anaphase-promoting complex-dependent proteasomal ubiquitin-dependent protein catabolic process |
| CDC2 | NDT | 24 | cell division |
| CDC2 | NDT | 24 | positive regulation of ubiquitin-protein ligase activity during mitotic cell cycle |
| CD9 | NDT | 6 | cell motility |
| CD9 | NDT | 6 | cell adhesion |
| CD9 | NDT | 6 | fusion of sperm to egg plasma membrane |
| CD9 | NDT | 6 | platelet activation |
| CD9 | NDT | 6 | paranodal junction assembly |
| CD47 | NDT | 6 | cell adhesion |
| CD47 | NDT | 6 | integrin-mediated signaling pathway |
| CD47 | NDT | 6 | positive regulation of cell proliferation |
| CD47 | NDT | 6 | positive regulation of cell-cell adhesion |
| CD47 | NDT | 6 | positive regulation of T cell activation |
| CD44 | NDT | 12 | cell adhesion |
| CD44 | NDT | 12 | cell-matrix adhesion |
| CD44 | NDT | 12 | cell-cell adhesion |
| CD40 | NDT | 8 | apoptosis |
| CD40 | NDT | 8 | immune response |
| CD40 | NDT | 8 | signal transduction |
| CD40 | NDT | 8 | protein complex assembly |
| CD40 | NDT | 8 | inflammatory response |
| CD40 | NDT | 8 | platelet activation |
| CD40 | NDT | 8 | B cell proliferation |
| CD40 | NDT | 8 | positive regulation of I-kappaB kinase |
| CD4 | NDT | 20 | conjugation with cellular fusion |
| CD4 | NDT | 20 | cytokine production |
| CD4 | NDT | 20 | immune response |
| CD4 | NDT | 20 | cell adhesion |
| CD4 | NDT | 20 | transmembrane receptor protein tyrosine kinase signaling pathway |
| CD4 | NDT | 20 | initiation of viral infection |
| CD4 | NDT | 20 | interspecies interaction between organisms |
| CD4 | NDT | 20 | T cell selection |
| CD4 | NDT | 20 | positive regulation of interleukin-2 biosynthetic process |
| CD4 | NDT | 20 | positive regulation of protein kinase activity |
| CD4 | NDT | 20 | positive regulation of peptidyl-tyrosine phosphorylation |
| CD4 | NDT | 20 | positive regulation of calcium-mediated signaling |
| CD4 | NDT | 20 | positive regulation of T cell activation |
| CD3Z | NDT | 13 | cell surface receptor linked signal transduction |
| CD3Z | NDT | 13 | interspecies interaction between organisms |
| CD2 | DT | 11 | membrane raft polarization |
| CD2 | DT | 11 | induction of apoptosis |
| CD2 | DT | 11 | cell surface receptor linked signal transduction |
| CD2 | DT | 11 | cell-cell adhesion |
| CD2 | DT | 11 | natural killer cell activation |
| CD2 | DT | 11 | positive regulation of myeloid dendritic cell activation |
| CD2 | DT | 11 | T cell activation |
| CD2 | DT | 11 | regulation of T cell differentiation |
| CCNK | NDT | 16 | regulation of cyclin-dependent protein kinase activity |
| CCNK | NDT | 16 | transcription |
| CCNK | NDT | 16 | regulation of transcription DNA-dependent |
| CCNK | NDT | 16 | transcription from RNA polymerase II promoter |
| CCNK | NDT | 16 | cell cycle |
| CCNK | NDT | 16 | mitosis |
| CCNK | NDT | 16 | cell division |
| CCND3 | NDT | 14 | positive regulation of protein amino acid phosphorylation |
| CCND3 | NDT | 14 | cell cycle |
| CCND3 | NDT | 14 | positive regulation of cyclin-dependent protein kinase activity |
| CCND3 | NDT | 14 | cell division |
| CCNA1 | NDT | 14 | cell cycle |
| CCNA1 | NDT | 14 | mitosis |
| CCNA1 | NDT | 14 | male meiosis I |
| CCNA1 | NDT | 14 | spermatogenesis |
| CCNA1 | NDT | 14 | cell division |
| CBX5 | NDT | 7 | chromatin assembly or disassembly |
| CBX5 | NDT | 7 | negative regulation of transcription |
| CBLB | NDT | 14 | ubiquitin cycle |
| CBLB | NDT | 14 | NLS-bearing substrate import into nucleus |
| CBLB | NDT | 14 | immune response |
| CBLB | NDT | 14 | cell surface receptor linked signal transduction |
| CAV1 | NDT | 18 | inactivation of MAPK activity |
| CAV1 | NDT | 18 | vasculogenesis |
| CAV1 | NDT | 18 | response to hypoxia |
| CAV1 | NDT | 18 | negative regulation of endothelial cell proliferation |
| CAV1 | NDT | 18 | triacylglycerol metabolic process |
| CAV1 | NDT | 18 | calcium ion transport |
| CAV1 | NDT | 18 | cellular calcium ion homeostasis |
| CAV1 | NDT | 18 | endocytosis |
| CAV1 | NDT | 18 | regulation of smooth muscle contraction |
| CAV1 | NDT | 18 | skeletal muscle development |
| CAV1 | NDT | 18 | protein localization |
| CAV1 | NDT | 18 | vesicle organization and biogenesis |
| CAV1 | NDT | 18 | regulation of fatty acid metabolic process |
| CAV1 | NDT | 18 | sequestering of lipid |
| CAV1 | NDT | 18 | regulation of blood coagulation |
| CAV1 | NDT | 18 | cholesterol transport |
| CAV1 | NDT | 18 | negative regulation of epithelial cell differentiation |
| CAV1 | NDT | 18 | nitric oxide homeostasis |
| CAV1 | NDT | 18 | cholesterol homeostasis |
| CAV1 | NDT | 18 | negative regulation of MAPKKK cascade |
| CAV1 | NDT | 18 | negative regulation of nitric oxide biosynthetic process |
| CAV1 | NDT | 18 | positive regulation of vasoconstriction |
| CAV1 | NDT | 18 | negative regulation of vasodilation |
| CAV1 | NDT | 18 | negative regulation of JAK-STAT cascade |
| CAV1 | NDT | 18 | positive regulation of metalloenzyme activity |
| CAV1 | NDT | 18 | protein homooligomerization |
| CAV1 | NDT | 18 | membrane depolarization |
| CAV1 | NDT | 18 | regulation of peptidase activity |
| CAV1 | NDT | 18 | mammary gland involution |
| CATSPER1 | NDT | 7 | ion transport |
| CATSPER1 | NDT | 7 | calcium ion transport |
| CATSPER1 | NDT | 7 | multicellular organismal development |
| CATSPER1 | NDT | 7 | spermatogenesis |
| CATSPER1 | NDT | 7 | cell differentiation |
| CASP9 | NDT | 7 | proteolysis |
| CASP9 | NDT | 7 | apoptosis |
| CASP9 | NDT | 7 | regulation of apoptosis |
| CASP9 | NDT | 7 | apoptotic program |
| CASP9 | NDT | 7 | caspase activation via cytochrome c |
| CASP7 | DT | 9 | apoptosis |
| CASP7 | DT | 9 | proteolysis |
| CASP7 | DT | 9 | apoptotic program |
| CASP3 | DT | 23 | proteolysis |
| CASP3 | DT | 23 | apoptosis |
| CASP3 | DT | 23 | induction of apoptosis |
| CASP3 | DT | 23 | nuclear fragmentation during apoptosis |
| CASP3 | DT | 23 | negative regulation of apoptosis |
| CASP2 | NDT | 9 | proteolysis |
| CASP2 | NDT | 9 | anti-apoptosis |
| CASP2 | NDT | 9 | regulation of apoptosis |
| CASP2 | NDT | 9 | apoptotic program |
| CAPN1 | NDT | 6 | proteolysis |
| CAPN1 | NDT | 6 | positive regulation of cell proliferation |
| CANX | NDT | 10 | angiogenesis |
| CANX | NDT | 10 | protein folding |
| CANX | NDT | 10 | protein secretion |
| CALR | NDT | 8 | regulation of transcription DNA-dependent |
| CALR | NDT | 8 | protein folding |
| CALR | NDT | 8 | protein export from nucleus |
| CALR | NDT | 8 | cellular calcium ion homeostasis |
| CALR | NDT | 8 | cortical actin cytoskeleton organization and biogenesis |
| CALR | NDT | 8 | regulation of meiosis |
| CALR | NDT | 8 | regulation of apoptosis |
| CALM3 | NDT | 9 | G-protein coupled receptor protein signaling pathway |
| CALM2 | NDT | 11 | G-protein coupled receptor protein signaling pathway |
| CALM1 | DT | 14 | G-protein coupled receptor protein signaling pathway |
| CACYBP | NDT | 6 | ubiquitin cycle |
| BUB1B | NDT | 6 | apoptosis |
| BUB1B | NDT | 6 | spindle organization and biogenesis |
| BUB1B | NDT | 6 | mitosis |
| BUB1B | NDT | 6 | mitotic cell cycle checkpoint |
| BUB1B | NDT | 6 | cell proliferation |
| BUB1B | NDT | 6 | anaphase-promoting complex-dependent proteasomal ubiquitin-dependent protein catabolic process |
| BUB1B | NDT | 6 | negative regulation of cell cycle |
| BUB1B | NDT | 6 | phosphoinositide-mediated signaling |
| BUB1B | NDT | 6 | cell division |
| BUB1B | NDT | 6 | negative regulation of ubiquitin-protein ligase activity during mitotic cell cycle |
| BTRC | NDT | 11 | ubiquitin-dependent protein catabolic process |
| BTRC | NDT | 11 | ubiquitin cycle |
| BTRC | NDT | 11 | signal transduction |
| BTRC | NDT | 11 | Wnt receptor signaling pathway |
| BTRC | NDT | 11 | interspecies interaction between organisms |
| BTRC | NDT | 11 | positive regulation of ubiquitin-protein ligase activity during mitotic cell cycle |
| BMX | NDT | 8 | protein amino acid phosphorylation |
| BMX | NDT | 8 | intracellular signaling cascade |
| BMX | NDT | 8 | mesoderm development |
| BLNK | NDT | 10 | inflammatory response |
| BLNK | NDT | 10 | humoral immune response |
| BLNK | NDT | 10 | intracellular signaling cascade |
| BLNK | NDT | 10 | B cell differentiation |
| BIRC5 | NDT | 6 | G2 |
| BIRC5 | NDT | 6 | cytokinesis |
| BIRC5 | NDT | 6 | apoptosis |
| BIRC5 | NDT | 6 | anti-apoptosis |
| BIRC5 | NDT | 6 | cell cycle |
| BIRC5 | NDT | 6 | mitosis |
| BIRC5 | NDT | 6 | protein complex localization |
| BIRC5 | NDT | 6 | positive regulation of exit from mitosis |
| BIRC5 | NDT | 6 | spindle checkpoint |
| BIRC5 | NDT | 6 | negative regulation of caspase activity |
| BIRC5 | NDT | 6 | positive regulation of mitotic cell cycle |
| BIRC5 | NDT | 6 | establishment of chromosome localization |
| BIRC4 | DT | 9 | ubiquitin cycle |
| BIRC4 | DT | 9 | apoptosis |
| BIRC4 | DT | 9 | anti-apoptosis |
| BIRC4 | DT | 9 | negative regulation of caspase activity |
| BIRC2 | NDT | 8 | response to hypoxia |
| BIRC2 | NDT | 8 | placenta development |
| BIRC2 | NDT | 8 | cell surface receptor linked signal transduction |
| BIRC2 | NDT | 8 | regulation of apoptosis |
| BIRC2 | NDT | 8 | positive regulation of I-kappaB kinase |
| BCL2L1 | NDT | 25 | regulation of apoptosis |
| BCL2L1 | NDT | 25 | release of cytochrome c from mitochondria |
| BCL2L1 | NDT | 25 | anti-apoptosis |
| BCL2L1 | NDT | 25 | negative regulation of survival gene product expression |
| BCL2L1 | NDT | 25 | regulation of mitochondrial membrane permeability |
| BCL2L1 | NDT | 25 | regulation of mitochondrial membrane potential |
| BCL2A1 | NDT | 6 | anti-apoptosis |
| BCL2A1 | NDT | 6 | regulation of apoptosis |
| BAX | NDT | 7 | regulation of apoptosis |
| BAX | NDT | 7 | induction of apoptosis |
| BAX | NDT | 7 | caspase activation |
| BAX | NDT | 7 | regulation of protein homodimerization activity |
| BAX | NDT | 7 | regulation of protein heterodimerization activity |
| BAX | NDT | 7 | positive regulation of neuron apoptosis |
| BAX | NDT | 7 | negative regulation of cell cycle |
| BATF | NDT | 9 | transcription |
| BATF | NDT | 9 | regulation of transcription DNA-dependent |
| BAT8 | NDT | 15 | biological process |
| BAT8 | NDT | 15 | chromatin modification |
| BAT5 | NDT | 7 | defense response |
| BAT5 | NDT | 7 | response to unfolded protein |
| BAT5 | NDT | 7 | response to nutrient |
| BAT3 | NDT | 10 | protein modification process |
| BAK1 | NDT | 6 | regulation of apoptosis |
| BAK1 | NDT | 6 | release of cytochrome c from mitochondria |
| BAK1 | NDT | 6 | induction of apoptosis |
| BAK1 | NDT | 6 | establishment and |
| BAK1 | NDT | 6 | regulation of protein homodimerization activity |
| BAK1 | NDT | 6 | regulation of protein heterodimerization activity |
| BAK1 | NDT | 6 | regulation of mitochondrial membrane permeability |
| BAK1 | NDT | 6 | regulation of mitochondrial membrane potential |
| BAG3 | NDT | 6 | protein folding |
| BAG3 | NDT | 6 | apoptosis |
| BAG3 | NDT | 6 | anti-apoptosis |
| BAG1 | NDT | 11 | protein modification process |
| BAG1 | NDT | 11 | apoptosis |
| BAG1 | NDT | 11 | anti-apoptosis |
| BAG1 | NDT | 11 | cell surface receptor linked signal transduction |
| BAD | NDT | 8 | induction of apoptosis |
| BAD | NDT | 8 | activation of pro-apoptotic gene products |
| B2M | NDT | 9 | antigen processing and presentation of peptide antigen via MHC class I |
| B2M | NDT | 9 | immune response |
| ATF4 | NDT | 16 | gluconeogenesis |
| ATF4 | NDT | 16 | transcription |
| ATF4 | NDT | 16 | regulation of transcription DNA-dependent |
| ATF4 | NDT | 16 | amino acid metabolic process |
| ATF4 | NDT | 16 | response to stress |
| ATF4 | NDT | 16 | positive regulation of transcription from RNA polymerase II promoter |
| ATF3 | NDT | 6 | regulation of transcription DNA-dependent |
| ARRB1 | NDT | 11 | signal transduction |
| ARRB1 | NDT | 11 | sensory perception |
| ARRB1 | NDT | 11 | response to stimulus |
| ARHGAP1 | NDT | 7 | signal transduction |
| ARHGAP1 | NDT | 7 | small GTPase mediated signal transduction |
| ARHGAP1 | NDT | 7 | Rho protein signal transduction |
| ARHGAP1 | NDT | 7 | regulation of GTPase activity |
| ARFIP2 | NDT | 8 | cell motility |
| ARFIP2 | NDT | 8 | small GTPase mediated signal transduction |
| ARFIP2 | NDT | 8 | lamellipodium biogenesis |
| ARFIP2 | NDT | 8 | actin cytoskeleton organization and biogenesis |
| ARFIP2 | NDT | 8 | ruffle organization and biogenesis |
| ARF6 | NDT | 6 | liver development |
| ARF6 | NDT | 6 | apoptosis |
| ARF6 | NDT | 6 | cell motility |
| ARF6 | NDT | 6 | cell adhesion |
| ARF6 | NDT | 6 | small GTPase mediated signal transduction |
| ARF6 | NDT | 6 | protein transport |
| ARF6 | NDT | 6 | vesicle-mediated transport |
| ARF6 | NDT | 6 | positive regulation of actin filament polymerization |
| ARF6 | NDT | 6 | cortical actin cytoskeleton organization and biogenesis |
| ARF6 | NDT | 6 | ruffle organization and biogenesis |
| ARF6 | NDT | 6 | regulation of Rac protein signal transduction |
| ARF6 | NDT | 6 | negative regulation of receptor-mediated endocytosis |
| ARF1 | NDT | 10 | retrograde vesicle-mediated transport Golgi to ER |
| ARF1 | NDT | 10 | small GTPase mediated signal transduction |
| ARF1 | NDT | 10 | protein transport |
| ARF1 | NDT | 10 | membrane organization and biogenesis |
| ARF1 | NDT | 10 | vesicle-mediated transport |
| ARF1 | NDT | 10 | COPI coating of Golgi vesicle |
| ARAF | DT | 8 | intracellular signaling cascade |
| ARAF | DT | 8 | protein modification process |
| ARAF | DT | 8 | protein amino acid phosphorylation |
| AQP1 | NDT | 6 | transport |
| AQP1 | NDT | 6 | water transport |
| AQP1 | NDT | 6 | excretion |
| APPBP2 | NDT | 19 | intracellular protein transport |
| APP | NDT | 21 | cellular copper ion homeostasis |
| APP | NDT | 21 | endocytosis |
| APP | NDT | 21 | apoptosis |
| APP | NDT | 21 | cell adhesion |
| APP | NDT | 21 | Notch signaling pathway |
| APP | NDT | 21 | neuromuscular process |
| APOA1 | NDT | 6 | negative regulation of cytokine secretion during immune response |
| APOA1 | NDT | 6 | lipid metabolic process |
| APOA1 | NDT | 6 | phosphatidylcholine biosynthetic process |
| APOA1 | NDT | 6 | lipid transport |
| APOA1 | NDT | 6 | G-protein coupled receptor protein signaling pathway |
| APOA1 | NDT | 6 | steroid metabolic process |
| APOA1 | NDT | 6 | cholesterol metabolic process |
| APOA1 | NDT | 6 | Cdc42 protein signal transduction |
| APOA1 | NDT | 6 | cholesterol efflux |
| APOA1 | NDT | 6 | phospholipid efflux |
| APOA1 | NDT | 6 | lipoprotein biosynthetic process |
| APOA1 | NDT | 6 | cholesterol homeostasis |
| APOA1 | NDT | 6 | reverse cholesterol transport |
| APOA1 | NDT | 6 | negative regulation of interleukin-1 beta secretion |
| APOA1 | NDT | 6 | protein stabilization |
| APEX2 | NDT | 8 | DNA repair |
| APEX1 | NDT | 12 | base-excision repair |
| APEX1 | NDT | 12 | transcription from RNA polymerase II promoter |
| APEX1 | NDT | 12 | regulation of cell redox homeostasis |
| APEX1 | NDT | 12 | regulation of DNA binding |
| AP2M1 | NDT | 14 | intracellular protein transport |
| AP2M1 | NDT | 14 | vesicle-mediated transport |
| AP2M1 | NDT | 14 | regulation of defense response to virus by virus |
| AP2B1 | NDT | 21 | intracellular protein transport |
| AP2B1 | NDT | 21 | vesicle-mediated transport |
| AP2B1 | NDT | 21 | regulation of defense response to virus by virus |
| AP2A1 | NDT | 6 | intracellular protein transport |
| AP2A1 | NDT | 6 | Golgi to endosome transport |
| AP2A1 | NDT | 6 | endocytosis |
| AP2A1 | NDT | 6 | regulation of defense response to virus by virus |
| AP1M2 | NDT | 7 | protein targeting |
| AP1M2 | NDT | 7 | vesicle targeting |
| AP1M1 | NDT | 14 | intracellular protein transport |
| AP1M1 | NDT | 14 | vesicle-mediated transport |
| ANXA2 | NDT | 11 | skeletal development |
| AKT1 | DT | 13 | protein import into nucleus translocation |
| AKT1 | DT | 13 | blood vessel development |
| AKT1 | DT | 13 | placenta development |
| AKT1 | DT | 13 | carbohydrate metabolic process |
| AKT1 | DT | 13 | glycogen biosynthetic process |
| AKT1 | DT | 13 | glucose metabolic process |
| AKT1 | DT | 13 | regulation of translation |
| AKT1 | DT | 13 | protein modification process |
| AKT1 | DT | 13 | nitric oxide biosynthetic process |
| AKT1 | DT | 13 | activated T cell apoptosis |
| AKT1 | DT | 13 | inflammatory response |
| AKT1 | DT | 13 | G-protein coupled receptor protein signaling pathway |
| AKT1 | DT | 13 | intracellular signaling cascade |
| AKT1 | DT | 13 | germ cell development |
| AKT1 | DT | 13 | insulin receptor signaling pathway |
| AKT1 | DT | 13 | activation of pro-apoptotic gene products |
| AKT1 | DT | 13 | apoptotic mitochondrial changes |
| AKT1 | DT | 13 | carbohydrate transport |
| AKT1 | DT | 13 | response to heat |
| AKT1 | DT | 13 | response to hormone stimulus |
| AKT1 | DT | 13 | glucose transport |
| AKT1 | DT | 13 | protein ubiquitination |
| AKT1 | DT | 13 | peptidyl-serine phosphorylation |
| AKT1 | DT | 13 | protein catabolic process |
| AKT1 | DT | 13 | positive regulation of multicellular organism growth |
| AKT1 | DT | 13 | anagen |
| AKT1 | DT | 13 | protein kinase B signaling cascade |
| AKT1 | DT | 13 | regulation of survival gene product expression |
| AKT1 | DT | 13 | protein amino acid autophosphorylation |
| AKT1 | DT | 13 | insulin-like growth factor receptor signaling pathway |
| ADRBK1 | DT | 9 | regulation of the force of heart contraction |
| ADRBK1 | DT | 9 | desensitization of G-protein coupled receptor protein signaling pathway |
| ADRBK1 | DT | 9 | negative regulation of the force of heart contraction by chemical signal |
| ADRBK1 | DT | 9 | signal transduction |
| ADRBK1 | DT | 9 | acetylcholine receptor signaling muscarinic pathway |
| ADRBK1 | DT | 9 | tachykinin signaling pathway |
| ADRBK1 | DT | 9 | heart development |
| ADRBK1 | DT | 9 | peptidyl-serine phosphorylation |
| ADRBK1 | DT | 9 | positive regulation of catecholamine secretion |
| ADRBK1 | DT | 9 | negative regulation of striated muscle contraction |
| ADRBK1 | DT | 9 | cardiac muscle contraction |
| ADAM15 | NDT | 8 | proteolysis |
| ADAM15 | NDT | 8 | cell adhesion |
| ADAM15 | NDT | 8 | cell-matrix adhesion |
| ACVR1 | DT | 8 | G1 |
| ACVR1 | DT | 8 | patterning of blood vessels |
| ACVR1 | DT | 8 | urogenital system development |
| ACVR1 | DT | 8 | in utero embryonic development |
| ACVR1 | DT | 8 | mesoderm formation |
| ACVR1 | DT | 8 | neural crest cell migration |
| ACVR1 | DT | 8 | acute inflammatory response |
| ACVR1 | DT | 8 | protein amino acid phosphorylation |
| ACVR1 | DT | 8 | transforming growth factor beta receptor signaling pathway |
| ACVR1 | DT | 8 | germ cell development |
| ACVR1 | DT | 8 | determination of left |
| ACVR1 | DT | 8 | gastrulation |
| ACVR1 | DT | 8 | mesoderm development |
| ACVR1 | DT | 8 | heart development |
| ACVR1 | DT | 8 | regulation of ossification |
| ACVR1 | DT | 8 | positive regulation of bone mineralization |
| ACVR1 | DT | 8 | BMP signaling pathway |
| ACVR1 | DT | 8 | negative regulation of activin receptor signaling pathway |
| ACVR1 | DT | 8 | negative regulation of apoptosis |
| ACVR1 | DT | 8 | positive regulation of osteoblast differentiation |
| ACVR1 | DT | 8 | positive regulation of transcription |
| ACVR1 | DT | 8 | regulation of skeletal muscle development |
| ACVR1 | DT | 8 | smooth muscle cell differentiation |
| ACTN4 | NDT | 6 | response to hypoxia |
| ACTN4 | NDT | 6 | positive regulation of sodium hydrogen antiporter activity |
| ACTN4 | NDT | 6 | regulation of apoptosis |
| ACTN4 | NDT | 6 | positive regulation of pinocytosis |
| ACTN4 | NDT | 6 | actin filament bundle formation |
| ACTN4 | NDT | 6 | negative regulation of cell motility |
| ACTN4 | NDT | 6 | positive regulation of cell motility |
| ACTN1 | NDT | 16 | regulation of apoptosis |
| ACTN1 | NDT | 16 | focal adhesion formation |
| ACTN1 | NDT | 16 | actin filament bundle formation |
| ACTN1 | NDT | 16 | negative regulation of cell motility |
| ACTG1 | NDT | 6 | cell motility |
| ACTG1 | NDT | 6 | sensory perception of sound |
| ACTG1 | NDT | 6 | response to calcium ion |
| ACTC | NDT | 12 | apoptosis |
| ACTC | NDT | 12 | actin filament-based movement |
| ACTC | NDT | 12 | muscle thin filament assembly |
| ACTC | NDT | 12 | actomyosin structure organization and biogenesis |
| ACTC | NDT | 12 | cardiac myofibril assembly |
| ACTC | NDT | 12 | cardiac muscle morphogenesis |
| ACTC | NDT | 12 | heart contraction |
| ACTB | NDT | 23 | cell motility |
| ACTB | NDT | 23 | sensory perception of sound |
| ACTB | NDT | 23 | response to calcium ion |
| ACTA1 | NDT | 9 | muscle contraction |
| ACTA1 | NDT | 9 | muscle thin filament assembly |
| ACTA1 | NDT | 9 | skeletal muscle fiber development |
| ABLIM1 | NDT | 10 | cytoskeleton organization and biogenesis |
| ABLIM1 | NDT | 10 | visual perception |
| ABLIM1 | NDT | 10 | organ morphogenesis |
| ABI3 | NDT | 11 | cell motility |
| ABI3 | NDT | 11 | peptidyl-tyrosine phosphorylation |
| ABI3 | NDT | 11 | regulation of cell migration |
| ABI2 | NDT | 11 | cytoskeleton organization and biogenesis |
| ABI2 | NDT | 11 | actin polymerization and |
| ABI2 | NDT | 11 | cell migration |
| ABI2 | NDT | 11 | peptidyl-tyrosine phosphorylation |
| 76P | NDT | 16 | protein complex assembly |
| 76P | NDT | 16 | microtubule nucleation |

a. Open reading frame name.

b. DT and NDT represent that a protein is drug target and non-drug target, respectively.
